# Amino Acid Substitutions in Cold-Adapted Proteins from *Halorubrum lacusprofundi*, an Extremely Halophilic Microbe from Antarctica

**DOI:** 10.1371/journal.pone.0058587

**Published:** 2013-03-11

**Authors:** Shiladitya DasSarma, Melinda D. Capes, Ram Karan, Priya DasSarma

**Affiliations:** Department of Microbiology and Immunology, and Institute of Marine and Environmental Technology, University of Maryland, Baltimore, Maryland, United States of America; University of South Florida College of Medicine, United States of America

## Abstract

The halophilic Archaeon *Halorubrum lacusprofundi*, isolated from the perennially cold and hypersaline Deep Lake in Antarctica, was recently sequenced and compared to 12 Haloarchaea from temperate climates by comparative genomics. Amino acid substitutions for 604 *H. lacusprofundi* proteins belonging to conserved haloarchaeal orthologous groups (cHOGs) were determined and found to occur at 7.85% of positions invariant in proteins from mesophilic Haloarchaea. The following substitutions were observed most frequently: (a) glutamic acid with aspartic acid or alanine; (b) small polar residues with other small polar or non-polar amino acids; (c) small non-polar residues with other small non-polar residues; (d) aromatic residues, especially tryptophan, with other aromatic residues; and (e) some larger polar residues with other similar residues. Amino acid substitutions for a cold-active *H. lacusprofundi* β-galactosidase were then examined in the context of a homology modeled structure at residues invariant in homologous enzymes from mesophilic Haloarchaea. Similar substitutions were observed as in the genome-wide approach, with the surface accessible regions of β-galactosidase displaying reduced acidity and increased hydrophobicity, and internal regions displaying mainly subtle changes among smaller non-polar and polar residues. These findings are consistent with *H. lacusprofundi* proteins displaying amino acid substitutions that increase structural flexibility and protein function at low temperature. We discuss the likely mechanisms of protein adaptation to a cold, hypersaline environment on Earth, with possible relevance to life elsewhere.

## Introduction

The surface of Earth is mostly covered by salty oceans, 90% of which are at temperatures of 5°C or lower. Many perennially cold environments are also hypersaline, with higher NaCl concentrations than seawater, especially in the Arctic and Antarctic regions, due to brine exclusion from sea ice and concentration by evaporitic processes. Extremophilic microbes are abundant in such extreme environments and are of growing interest from the perspective of basic biology and biotechnology [1]–[5]. Evidence for the existence of cold hypersaline brines elsewhere in our solar system has also been provided [6]–[8]. As a result, the mechanisms of survival of extremophiles in freezing conditions and saturating salinity are also garnering attention in the field of astrobiology [9].

*Halorubrum lacusprofundi*, a member of the ancient class of microorganisms in the Domain Archaea, is one of the few cold and salt-adapted species available in pure culture [10]. This microbe was isolated from Deep Lake, Antarctica where temperatures remain below 0°C for 8 months of the year. Due to the extremely high salinity of Deep Lake, reported as ∼ 3.5 M NaCl, the freezing point is greatly depressed and the lake remains liquid throughout the year, even when temperatures reach a minimum of −18°C during the winter. In the laboratory, *H. lacusprofundi* is a psychrotolerant microorganism capable of growth at sub-zero temperatures, while its optimum growth temperature is 30°C, lower than most other related haloarchaeal microorganisms (Table 1) [11], [12].

**Table 1:** Characteristics of sequenced Haloarchaea and the 604 cHOGs included in study.

Haloarchaeon	Genome size (Mbp)	Growth temperature range (^o^C)	Average proteome MW	Average proteome pI		
*Halorubrum lacusprofundi*	3.7	−2–42	32.27		4.39	
*Halobacterium* sp. NRC-1	2.6	15–50	31.06		4.59	
*Halorhabdus utahensis*	3.1	17–55	31.96		4.40	
*Haloferax volcanii*	4.0	20–49	31.63		4.55	
*Halalkalicoccus jeotgali*	3.7	21–50	31.48		4.57	
*Halogeometricum borinquense*	3.9	22–58	32.18		4.50	
*Natronomonas pharaonis*	2.8	23–56	31.58		4.40	
*Haloterrigena turkmenica*	5.4	23–57	32.43		4.33	
*Haloquadratum walsbyi*	3.2	25–55	33.32		4.61	
*Halopiger xanaduensis*	4.4	28–45	32.54		4.30	
*Halomicrobium mukohataei*	3.3	35–52	31.89		4.39	
*Natrialba magadii*	4.4	37–40*	32.95		4.32	
*Haloarcula marismortui*	4.3	40–50*	32.08		4.40	

NOTES: The proteome molecular weight (MW) and isoelectric point (pI) data are averages for the 604 protein families used in amino acid composition analysis. Growth temperature ranges are indicated, except those marked with *,where only the optimal growth temperatures were reported.

Recently, the genome of *H. lacusprofundi* was sequenced (http://www.ncbi.nlm.nih.gov/bioproject/18455), providing an opportunity to better understand its adaptation to a cold, hypersaline environment. The *H. lacusprofundi* genome is organized into chromosome I (2.7 Mbp), chromosome II (526 kbp), and a megaplasmid (431 kbp), and codes for ca. 3,560 proteins. Like most Haloarchaea, this organism has high GC-composition, with chromosome I having the highest (66.7% GC), and the two smaller replicons, lower GC content (57.1 and 54.9%, respectively) [13], [14]. Preliminary analysis of its genome sequence revealed 4 different *csp* genes, which are thought to be involved in the organisms’ cold response [15]. Recent comparative genomic analysis also identified 784 core haloarchaeal orthologous groups of proteins (cHOGs) conserved in *H. lacusprofundi* and 12 other Haloarchaea [16], [17]. These findings showed that the Haloarchaea constitute a well-defined and coherent phylogenetic group for comparative genomic studies.

An interesting region of the genome of *H. lacusprofundi* that has come under scrutiny is a gene cluster for carbohydrate utilization, including the gene for a glycoside hydrolase family 42 β-galactosidase enzyme named *bga* [18]. The *bga* gene was recently introduced into the genetically tractable haloarchaeon *Halobacterium* sp. NRC-1 and overexpressed under the control of a cold shock protein gene promoter [19]. After purification, the enzyme was found to be active over a wide range of temperatures, retaining a substantial fraction of its maximum activity even when temperatures were as low as −5°C. The cold-active β-galactosidase enzyme also exhibited extremely halophilic character, with maximal activity at 4 M concentrations of sodium or potassium chloride [19]. As a result, *H. lacusprofundi* β-galactosidase was found to constitute an excellent model enzyme for studying protein function at cold, hypersaline conditions.

Relatively few studies have thus far addressed the mechanisms of cold-adapted proteins in hypersaline conditions [1], [5], [20]–[22]. Such conditions increase the viscosity of the medium, decrease the solubility and flexibility of proteins, and reduce the speed of enzymatic reactions. Proteins active under hypersaline conditions are known to display high surface negative charges, which enhance binding of water and improve solubility [22]–[24]. Some cold active proteins have also been shown to display negative charges at the surface, and increase activity and improve folding at higher concentrations of salt [25], [26]. Therefore, adaptations in halophilic proteins may also increase conformational flexibility, allowing for “breathing” necessary for promoting catalysis at reduced temperatures. These findings suggest that synergistic mechanisms may be operating in high salt and low temperature conditions.

In order to address the adaptation of halophilic proteins to cold temperatures in more detail, we compared predicted proteins of the cold adapted halophilic species, *H. lacusprofundi*, to orthologous proteins conserved in mesophilic Haloarchaea. To provide structural context and interpret the significance of observed amino acid substitutions in genome-wide analysis, we utilized a model structure of the cold-active β-galactosidase of *H. lacusprofundi*. The bioinformatic results in this investigation reveal some key strategies in the design of *H. lacusprofundi* proteins, likely to be relevant for protein function in cold, hypersaline environments.

## Results

### Selection and Alignment of Orthologous Haloarchaeal Protein Sequences

Thirteen completely sequenced genomes of Haloarchaea available in NCBI were used for comparative genomic analysis, including 12 mesophilic species and the cold-adapted *H. lacusprofundi* (see Materials and Methods). The mesophilic species displayed growth ranges between ca. 20 and 50°C while *H. lacusprofundi* also grew at temperatures below 20°C down to −2°C (Table 1). Previously, we used best reciprocal blast analysis and protein clustering of these haloarchaeal predicted proteomes to establish 784 core haloarchaeal orthologous groups, or cHOGs, conserved in all of the Haloarchaea [17]. We selected 604 of these cHOG protein families that were unique in each of the 13 Haloarchaea for further analysis (Table S1).

**Figure 1:**
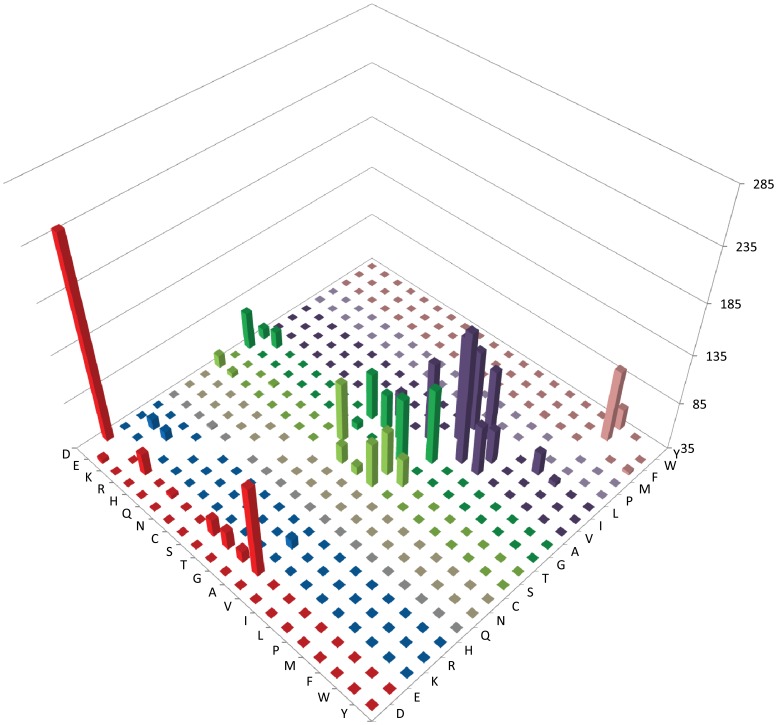
Amino acid substitution matrix of selected core haloarchaeal orthologous proteins for invariant residues in mesophilic versus cold-adapted *H. lacusprofundi*. Amino acids conserved in 604 protein families in 12 mesophilic sequences are indicated on the right-axis, *H. lacusprofundi* amino acids are on left-axis, and the number of amino acid substitutions on the vertical axis, with the floor placed at 35 amino acids, to emphasize higher peaks.

Preliminary analysis of the 604 cHOGs confirmed that the proteins are very similar in molecular weight (MW) and isoelectric point (pI) (Table 1). Protein MW averages for sequenced Haloarchaea ranged from 31.06–33.32, and 32.27 for *H. lacusprofundi*, while the protein pI averages ranged from 4.30–4.61, and 4.39 for *H. lacusprofundi.* In order to identify amino acid residues different in *H. lacusprofundi* proteins compared to the mesophilic orthologs, the 12 mesophilic haloarchaeal protein sequences for each of the 604 selected protein families were first aligned to each other to generate multiple sequence alignments [27]. Profiles of the mesophilic proteins were constructed for each protein family and then aligned with the orthologous *H. lacusprofundi* proteins, and the identity of residues varying in the cold-adapted species that were invariant in the mesophilic sequences (5,541 residues out of 70,589 total) were extracted. The specific amino acid substitutions observed between the mesophilic profiles and the *H. lacusprofundi* orthologs were tallied and plotted, illustrating the differences in amino acid composition in the cold-adapted species (Figure 1, Table 2, and Table S2) [27].

**Table 2:** Selected amino acid substitutions between mesophilic invariant residues in 604 cHOGs and *H. lacusprofundi* orthologs.

Mesophilic preference	*H. lacusprofundi* preference	*Number of substitutions	*Percent of substitutions
D	E	39	0.70
E	D	241	4.35
E	A	131	2.36
E	R	58	1.05
E	T	54	0.97
E	S	532	0.96
E	G	47	0.85
E	Q	39	0.70
R	E	46	0.83
R	A	45	0.81
R	K	45	0.81
Q	R	37	0.67
S	A	82	1.48
S	T	54	0.97
S	D	48	0.87
S	G	44	0.79
S	E	40	0.72
T	S	97	1.75
T	A	82	1.48
T	V	65	1.17
T	R	37	0.67
G	A	104	1.88
G	D	74	1.34
G	S	42	0.76
A	V	114	2.06
A	S	84	1.52
A	G	81	1.46
A	T	73	1.32
A	E	53	0.96
A	D	46	0.83
V	I	171	3.09
V	A	118	2.13
V	L	88	1.59
V	T	63	1.14
I	V	144	2.60
I	L	71	1.28
L	V	119	2.15
L	I	110	1.99
L	M	59	1.06
L	F	40	0.72
P	A	60	1.08
F	Y	38	0.69
W	F	110	1.99
Y	F	57	1.03

* A total of 5,541 invariant residues in mesophiles were substituted in *H. lacusprofundi* and those amino acid substitutions with more than 35 (0.67%) cases are shown.

### Genome-wide Analysis of Amino Acid Substitutions

The great majority of amino acid residues invariant in the selected 604 protein families in 12 mesophilic Haloarchaea were also conserved in *H. lacusprofundi*, ranging from 86.0–97.4% and averaging 92.2% conservation (Table S2). Of the 7.85% amino acid substitutions identified in *H. lacusprofundi* sequences, several amino acids were substituted more frequently than expected based on composition, especially glutamic acid (E), tryptophan (W), serine (S), isoleucine (I), and threonine (T) (Table S2). Some amino acids were substituted less frequently than expected, including aspartic acid (D), cysteine (C), glycine (G), phenylalanine (F), lysine (K), and proline (P). When the ratios of specific amino acid substitutions in *H. lacusprofundi* proteins to proteins of mesophilic Haloarchaea were calculated, increases in D and decreases in E and W were most clearly apparent (data not shown).

Most of the amino acid substitutions observed in *H. lacusprofundi* proteins resulted in subtle changes in size, charge, or hydrophobicity. When amino acid substitutions were displayed using a matrix, 2,706 substitutions out of 5,541 total or 48.8% were found to occur between highly similar amino acid residues (Figure 1 and Table S2). E was frequently replaced by D or alanine (A) (Table 2 and Table S2). Polar residues, T and S, and the small residues, G and A, were substituted with similar polar or non-polar amino acids. Non-polar residues, valine (V), I, and leucine (L), were usually substituted with one of the same three non-polar residues. Aromatic amino acids, especially W, were replaced with other aromatic residues. Several other amino acids were also replaced with other similar amino acids.

### Frequently Observed Amino Acid Substitutions in *H. lacusprofundi* Proteins

**Figure 2:**
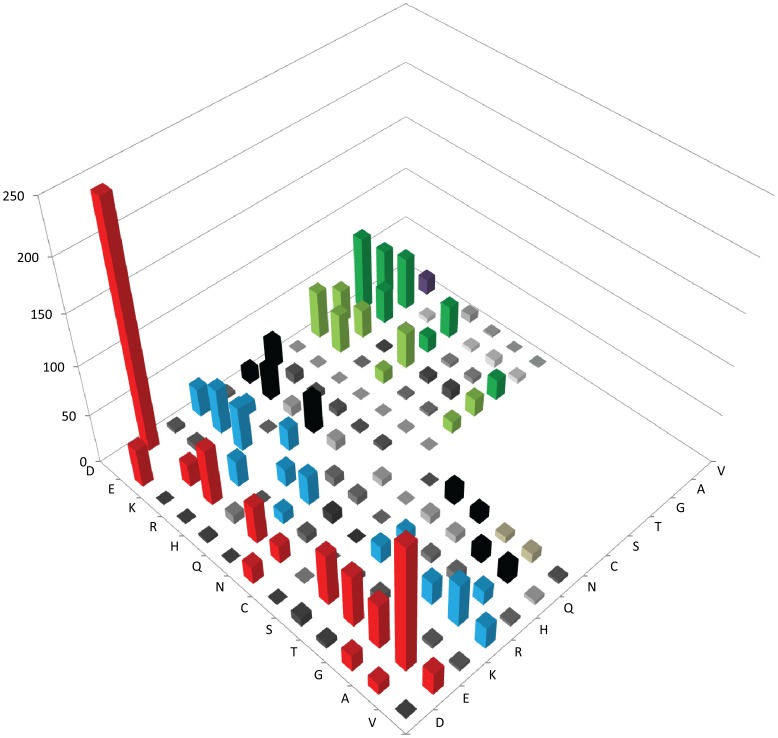
Amino acid substitution matrix of selected core haloarchaeal orthologous proteins for invariant negatively and positively charged and polar and small non-polar residues in mesophilic versus corresponding cold-adapted *H. lacusprofundi* proteins. Amino acids conserved in 604 protein families in 12 mesophilic sequences are indicated on the right-axis, *H. lacusprofundi* amino acids are on left-axis, and the number of amino acid substitutions on the vertical axis, with higher peaks (>10) colored for emphasis.

**Figure 3:**
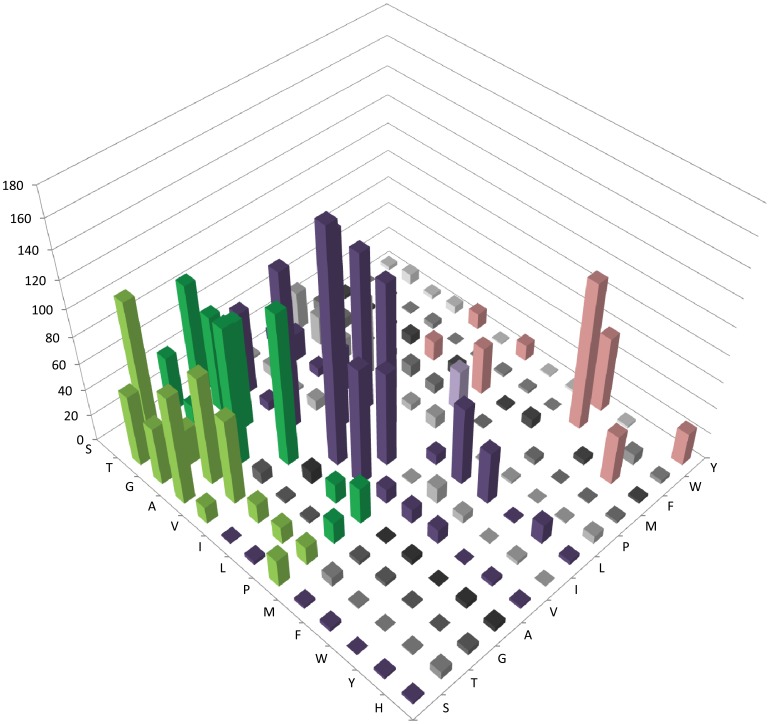
Amino acid substitution matrix of selected core haloarchaeal orthologous proteins for invariant non-polar residues in mesophilic versus cold-adapted *H. lacusprofundi* proteins. Amino acids conserved in 604 protein families in 12 mesophilic sequences are shown on the right-axis, *H. lacusprofundi* amino acids are on left-axis, and the number of amino acid substitutions on the vertical axis, with higher peaks (>10) colored for emphasis.

The amino acid substitutions that were frequently observed in the cold-adapted *H. lacusprofundi* species at invariant positions in the mesophilic proteins were analyzed by the total numbers and percent of substitutions, and percent of substitutions weighted by abundance of specific amino acids (Table 2, Figures 1, 2, 3, and Table S2). The substitution frequencies were very similar using all methods and were classified into the following categories:

#### (i) Substitutions of acidic, basic, and long polar residues

Overall, E was substituted most frequently among all of the amino acids (13.43% of total substitutions) (Figure 2 and Table S2). A large proportion of these were substituted with D (4.35%) and A (2.36%), which differ in presence or absence of the carboxylic acid group in the side chain. At lower frequencies, E was also substituted by the basic amino acid, arginine (R) (1.05%), the long polar amino acid, glutamine (Q) (0.70%), or the small polar amino acids, S, T, and G (0.85–0.97%). D was substituted at a fraction of the rate of E (only 1.89% of total substitutions), most frequently with E (0.70%).

Among basic residues, R substitutions were the most frequently observed, partly due to its high abundance (6.82% of total substitutions), while the less abundant K and histidine (H) residues were less frequently substituted (1.61 and 2.44% of total substitutions, respectively) (Figure 2 and Table S2). R was substituted most frequently with either K, E, or A (∼0.8% each of total substitutions) and less frequently with T, Q, D, or L (0.51–0.61% total substitutions range). Both K and H were substituted most frequently with R (less than 0.5% of total substitutions in both cases). Among amino acids with long polar side chains, asparagine (N) and Q were also less abundant than R, and substitutions were observed less frequently (2.09% and 3.25% of total substitutions, respectively), generally with similar size amino acids (Figure 2 and Table S2). Q was substituted most frequently with R or D (0.67% and 0.60%, respectively), while N was substituted most frequently with D (0.51%).

#### (ii) Substitutions of small polar residues

In most cases, polar residues T and S, and the smallest residues, G and A, were substituted with similar polar or non-polar amino acids, primarily S, T, G, A, or V and occasionally acidic residues (Figure 3 and Table S2). For S and T, which were substituted 6.21% and 8.36% of total frequency, respectively, the most common substitutions were with each other or A (0.97–1.75%), a similar size non-polar residue. Other less frequent substitutions for S were with D, E, or G (0.72–0.87%). For T, most substitutions were with the same three amino acids, R or V (0.51–1.17%).

For the small amino acids, G and A, the frequency of substitutions was considerably higher for the latter (10.40% of total) than the former (6.52% of total), while their abundance was similar. A was most frequently substituted by V, S, G, and T (1.32–2.06%), which are more similar in size and polarity, and less frequently with E, D, R, and P (0.51–0.96%), which are less similar. G was substituted most frequently by A or D (1.34–1.88%) and less frequently with S or E (0.61–0.76%).

#### (iii) Substitutions of small non-polar residues

The three non-polar residues, V, I, and L, were frequently substituted with the same non-polar residues, or in some cases A, methionine (M), or other similar residues (Figure 3 and Table S2). However, V and L were more abundant and more frequently substituted (9.37% and 8.46% of total) compared to I (4.76%). V was most frequently substituted by I, A, L, or T (1.14–3.09%). L was most frequently substituted with V or I (2.15% and 1.99%, respectively), and less frequently with M, F, or A (0.61–1.06%). I was most frequently substituted with V or L (2.60% and 1.28%, respectively).

#### (iv) Substitutions of aromatic and other residues

While W was one of the least abundant amino acids, it was one of the most frequently substituted, particularly with another large aromatic residue, F (1.99% of total and 11.07% of specific substitutions) (Figure 3 and Table S2). The two aromatic residues, F and tyrosine (Y), were somewhat more abundant than W, and were frequently replaced, usually with each other (0.69–1.03%), or with L (0.65% for F).

For the remaining residues, M was most commonly substituted with L (0.56%) and P was substituted most frequently with A, D, or S (0.52–1.08%) (Figure 3 and Table S2). C was the least abundant amino acid and the least likely to be substituted (0.47% of total substitutions), primarily with A or S.

### Analysis of the Cold-active β-galactosidase from *H. lacusprofundi*

The *H. lacusprofundi* genome harbors a gene coding for a glycosyl hydrolase family 42 β-galactosidase with homologs in four mesophilic Haloarchaea (*H. lucentense* SB1, *H. volcanii*, *H. turkmenica*, and *H. xanaduensis*) [18], [19], [28]. The proteins are very similar in MW (74.46–78.06), pI (4.18–4.28), and sequence (>60% identity). We aligned these five β-galactosidase protein sequences to first determine amino acid differences between mesophilic Haloarchaea and *H. lacusprofundi* homologs. The cold-active β-galactosidase from *H. lacusprofundi* deviated at 29 out of 321 invariant amino acid positions (9.03%) in mesophilic Haloarchaea, which is similar to the substitution frequency observed in comparative genomic analysis (7.85%) (Table 3). Of the 29 amino acid substitutions, 25 were distinct (with four substitutions occurring twice) and 18 (72%) occurred at high frequency (≥0.67%) in genome-wide analysis. Conversely, of the 44 highest frequency substitutions observed in genome-wide analysis, 16 substitutions (36%) were observed in β-galactosidase. These results show a good correlation between amino acid substitutions in β-galactosidase compared with those found in the 604 cHOGs analyzed by comparative genomics.

**Table 3:** Amino acid substitutions between invariant residues in family 42 β-galactosidases from mesophilic Haloarchaea and cold-active *H. lacusprofundi* β-galactosidase.

Mesophile amino acid	*H. lacusprofundi* amino acid	Residue number	MW change	Hydrophobicity change*	Surface (%)
D	S	189	−28	50	15
D	S	651	−28	50	20
D	N	251	−1	27	10
D	P	235	−18	9	25
E	Q	284	−1	21	<5(4)
E	S	451	−42	26	25
H	F	404	10	92	<5(3)
Q	A	23	−57	51	5
S	A	263	−16	46	<5(0)
S	C	324	16	54	15
T	R	19	55	−27	45
T	N	180	13	−41	<5(0)
T	A	181	−30	28	45
T	A	460	−30	28	<5(1)
G	R	643	99	−14	40
A	G	328	−14	−41	<5(4)
A	T	383	30	−28	<5(2)
A	S	384	16	−46	10
V	L	348	14	21	<5(2)
V	I	476	14	23	25
V	A	604	−28	−35	5
I	V	443	−14	−23	<5(1)
L	Q	427	15	−107	5
L	I	299	0	2	5
L	V	434	−14	−21	<5(3)
L	V	482	−14	−21	5
L	T	635	−12	−84	<5(1)
L	F	484	34	3	<5(2)
L	F	387	34	3	30

* Hydrophobicity was calculated based on ref. [66].

**Figure 4:**
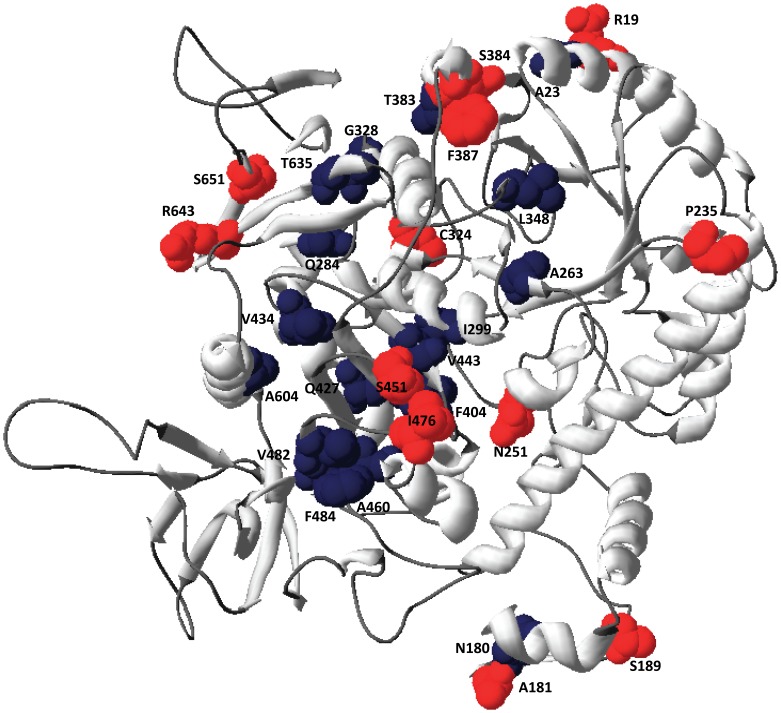
Model of *H. lacusprofundi* β-galactosidase highlighting differences with mesophilic Haloarchaea. The protein backbone is colored gray, substitutions of surface residues are shown colored red, and substitutions of internal residues are shown colored dark blue. The protein structure was illustrated using Swiss-PDBViewer [65].

Amino acid substitutions in the *H. lacusprofundi* β-galactosidase that were at positions invariant in the mesophile enzymes mapped to the model structure of the enzyme using DeepView (Table 3 and Figure 4). Seventeen amino acid residues that were ≤5% solvent-exposed were classified as buried, and twelve residues that were ≥10% solvent-exposed were classified as surface [29]. Five acidic residues (D and E), mapping to the surface of mesophilic proteins in the modeled structure were substituted either with polar residues (S, Q, or N), or P, increasing hydrophobicity and decreasing negative charges at the protein surface (Figure 4). Four other substitutions observed at the protein surface also increased hydrophobicity, specifically S with C, T with A, V with I, and L with F. Only three substitutions resulting in surface residues with decreased hydrophobicity were found, A with S, and T or G with R, the latter two of which mapped near the amino- or carboxy-termini of the protein, respectively.

Amino acid substitutions observed in internal regions of the *H. lacusprofundi* β-galactosidase were primarily in non-polar or small polar residues, usually with very similar residues (Table 3 and Figure 4). L was the most common amino acid to be substituted internally (6 residues), with V (X 2), I, Q, T, or F. A was substituted with G or T, V was substituted with L or A, while I was substituted with V. Q and T were substituted with A or N, and H was substituted with F. A single E residue with intermediate solvent exposure (4%) was substituted with Q. Overall, most substitutions in internal residues resulted in relatively small alterations in molecular weight and/or hydrophobicity.

## Discussion

We have explored the general amino acid sequence characteristics of over 600 core haloarchaeal orthologous protein families (cHOGs) and determined which invariant residues in mesophilic Haloarchaea were substituted in the cold-adapted *H. lacusprofundi* haloarchaeon. Interestingly, of the amino acid residues substituted in *H. lacusprofundi* (5,541/70,589 invariant or 7.85%), about half of the changes (2,706/5,541 or 48.8%) resulted in small alterations in molecular weight and/or polarity, e.g. in acidic amino acids like E, polar ones like T and S, non-polar residues like V and L, and aromatic amino acids, especially W. We also determined amino acid substitutions in the context of the modeled structure of *H. lacusprofundi* β-galactosidase at positions invariant in mesophilic homologs (29/321 or 9.03%). We again found substitutions primarily of non-polar and polar amino acids with similar residues, e.g. L with V, and T with A, in interior regions, and of charged amino acids with residues with increased hydrophobicity, e.g. D and E with S, at the surface. These approaches together confirmed that certain types of amino acid substitutions are commonly found in *H. lacusprofundi* proteins and are likely to be responsible for their adaptation to cold temperatures in a hypersaline environment.

Our findings are generally consistent with previous studies that have addressed amino acid composition in protein adaptation to low temperatures, such as the genome sequence analysis of the cold-growing bacterium *Colwellia psychrerythraea*, which found a lower proportion of charged amino acids in surface composition [30]. Another study that analyzed the sequence of nearly 400 cold-adapted proteins, also reported similar differences at the protein surface, consistent with alterations in amino acid contacts with the solvent [31]. Changes at the surface resulted in greater entropic effects rather than specific effects like diminished numbers of salt bridges. Other reports on temperature-dependent activity of proteins pointed to decreasing numbers of hydrogen bonds, bound ions, and salt bridges at the surface [32]. In our genome-wide analysis, more than a third of the observed substitutions in the *H. lacusprofundi* β-galactosidase were in surface residues, primarily at D and E, and the overwhelming majority of changes decreased charge and/or increased protein hydrophobicity at its surface. The amino acid changes increasing hydrophobicity at the enzyme surface are expected to diminish the network of hydrogen bonds around the protein, increasing structural flexibility necessary for maintaining catalytic activity in colder low-density water [33], [34]. The few observed changes increasing charges at the surface occurred primarily near the amino- and carboxy-termini of β-galactosidase, regions known to usually have greater solvent exposure and less rigid structure [35].

Previous studies have reported increases in small amino acids and decreases in the volume and mass per residue in proteins active in colder temperatures [36], [37]. Such changes may also result in decreasing hydrophobicity within the core of the enzyme [38], [39]. Consistent with these studies, in our comparative genome analysis, many of the substitutions identified in *H. lacusprofundi* proteins also decreased the size of the amino acid side chain, e.g. E, V, or T with A, I with V, and W with F, respectively. Similar results were also obtained for *H. lacusprofundi* β-galactosidase, where about one-half of internal substitutions resulted in smaller amino acid residues, while the other half resulted in larger residues in the cold-adapted protein. Both kinds of substitutions likely produce small perturbations in packing within internal regions of the protein structure, leading to an increase in protein flexibility and greater activity at lower temperatures.

Changes in the overall protein charge and content of acidic and basic amino acids, as well as P, have previously been reported in cold-active proteins [31], [32], [40]. For example, analysis of the complete genome sequence of *Psychrobacter arcticus*, a cold-growing bacterium from Siberian permafrost, showed the reduced use of P and R, in addition to acidic amino acids, in a significant portion of the predicted proteome [41]. In our genome-wide analysis, slight reduction in the content of P and R was also found in *H. lacusprofundi* proteins, while W content was significantly (13.2%) decreased, likely reflecting the trend toward smaller, more hydrophobic amino acids internally (Table S2). For acidic residues in *H. lacusprofundi* proteins, D content increased while E content decreased genome-wide, resulting in no net change in protein charge. The overall pIs and molecular weights for *H. lacusprofundi* proteins were not significantly altered compared to mesophilic Haloarchaea.

For β-galactosidase, as in the genome-wide analysis, a slight increase was observed in the content of D for the *H. lacusprofundi* enzyme, while the content of E was slightly reduced, without any significant change in overall charge. Interestingly, however, four D and one E residues conserved in the mesophilic enzymes were found to be substituted to uncharged residues of smaller size at the surface of the cold-adapted enzyme. These observations indicate that there are two countervailing forces at work in adaptation of this enzyme to cold temperatures at high salt concentrations. While surface negative charges are critical for protein function in high salinity, activity at cold temperatures likel requires limiting the number of negative charges at the surface in order to achieve the desired surface hydrophobicity [5]. As a result, the design of such novel salt and cold adapted proteins must involve careful selection of surface residues, a prediction which may be tested by genetic engineering of *H. lacusprofundi* proteins, and comparing results to investigations on other halophilic and cold-active β-galactosidases (e.g. [28], [42]).

Several mutagenesis studies have explored the importance of specific amino acid residues on protein function in cold temperature or high salinity. For example, when E residues on the protein surface were mutated in a halophilic, relatively salt insensitive TATA-binding protein, it was converted into non-halophilic, salt sensitive variants [43]. The investigation pointed to the importance of surface negative charges for the uptake of cations and discharge of water accompanying macromolecular complex formation at high salinity. When the cold adapted isocitrate lyase from *C. psychrerythraea* was mutagenized, an internal A residue was found to be critical for activity at cold temperatures [44]. In our analysis, surface and internal hydrophobic residues were similarly predicted to be important in the function of the cold and salt adapted β-galactosidase. The availability of the *H. lacusprofundi* enzyme expression system in *Halobacterium* sp. NRC-1 provides an ideal platform for testing the roles of those specific amino acid residues by mutagenesis in the future [19].

The adaptive mechanisms of proteins in cold brine are of significant interest, since most of the habitable environments on Earth are cold and salty. Genome-wide comparisons of proteins from *H. lacusprofundi*, a cold-adapted member of the ancient class of microorganisms in the Domain Archaea, to other mesophilic Haloarchaea growing at moderate temperatures have led to insights into polyextremophilic protein function. This study has provided a detailed catalog of the amino acid changes in proteins from the cold adapted organism in otherwise invariant residues in mesophiles. The cold-adapted proteins from *H. lacusprofundi* have more hydrophobic residues on the surface and subtle changes to many non-polar and polar residues buried in the interior, based on the structural model of a cold-active β-galactosidase enzyme. Amino acid substitutions observed in the cold adapted protein are consistent with small perturbations providing greater flexibility at colder temperatures. The general relevance of these results to cold-active proteins is underscored by similar findings in other microorganisms isolated from perennially cold systems, such as the Arctic and Antarctic oceans, Greenland glaciers, etc. [3], [45]–[47].

Since the Antarctic Deep Lake is one of the coldest and most extreme environments from which microbes have been cultured, the unusual and unique properties of *H. lacusprofundi* are of relevance to astrobiology [9]. Such Antarctic environments may be analogs of regions of Earth’s sister planet, Mars, where the potential for biological activity is of intense interest. Images from the Mars Reconnaissance Orbiter has shown evidence for seasonal emergence of liquid flows down steep rocky cliffs in summer, findings consistent with briny liquid water emerging from underground reservoirs on Mars [6]. Recent photographs from the Curiosity Rover have also suggested that running surface water was once prevalent on ancient Mars [48], although the temperature and salinity characteristics during that time are not yet known. On Europa, the entire surface is presently covered by frozen water-ice, and liquid water beneath the surface may be generated by dissipation of tidal forces [7], [8]. Additional studies of model polyextremophilic enzymes and cultured microbes from extreme environments on Earth are likely to provide further insights into how life may be able to cope with similar challenging conditions on other worlds.

## Materials and Methods

### Selection of Haloarchaeal Proteins

Predicted proteins were selected from the genome sequences of 13 Haloarchaea, 12 of which are mesophiles and 1 is a cold-adapted species, *H. lacusprofundi* (Table 1). We targeted proteins belonging to conserved haloarchaeal orthologous groups, or cHOGs, conserved in all 13 sequenced haloarchaeal genomes. Of the 784 cHOGs, 604 were found as a single copy in each genome, and did not contain any paralogs (Table S1). The sequences of proteins in the 604 non-paralogous clusters were obtained from NCBI for 13 Haloarchaea: *Halobacterium* sp. NRC-1 ATCC 700922 [49], *Haloarcula marismortui* ATCC 43049 [50], *Natronomonas pharaonis* DSM 2160 [51], *Haloquadratum walsbyi* DSM 16790 [52], *Halorubrum lacusprofundi* ATCC 49239 (http://www.ncbi.nlm.nih.gov/bioproject/18455), *Halogeometricum borinquense* DSM 11551 [53], *Halomicrobium mukohataei* DSM 12286 [54], *Halorhabdus utahensis* DSM 12940 [55], *Haloferax volcanii* DS2 ATCC 29605 [56], *Haloterrigena turkmenica* DSM 5511 [57], *Natrialba magadii* ATCC 43099 [58], *Halalkalicoccus jeotgali* B3 [59], and *Halopiger xanaduensis* SH-6 [60].

### Amino Acid Composition Analysis

In-house Perl scripts were used to facilitate bioinformatic analyses using the EMBOSS suite of programs [27]. Each of the 604 non-paralogous cHOG protein sequences were extracted and used to calculate molecular weights and pIs. Amino acid sequences of each cHOG from the 12 mesophilic Haloarchaea were aligned with progressive, pairwise alignments and the Blosum62 matrix [61], [62]. Consensus sequences were created for each position with 100% identity and compared to the protein sequence of the *H. lacusprofundi* ortholog for each cHOG. The resulting pairwise alignments containing the mesophilic identity and *H. lacusprofundi* sequence were used to identify and tabulate (a) positions where residues are conserved in the 12 mesophilic organisms and differ in *H. lacusprofundi*, and (b) positions where residues are conserved in all 13 sequences.

### β-galactosidase Protein Sequence Analysis

The amino acid sequence of cold-active β-galactosidase from *H. lacusprofundi* was aligned to mesophilic β-galactosidases from four Haloarchaea, *Haloferax lucentense* (*H. alicantei*), *H. volcanii*, *H. xanaduensis*, and *H. turkmenica* as described above. Invariant residues in the mesophilic haloarchaeal proteins which varied in *H. lacusprofundi* were tabulated [63]. The structure of *H. lacusprofundi* β-galactosidase was modeled and illustrated using the crystal structure of β-galactosidase from *Thermus thermophilus* A4 with the Swiss-PDBViewer [64], [65]. Solvent accessibility was calculated, and buried and surface residues defined as having accessibilities ≤5% and ≥10%, respectively [29].

## Supporting Information

Table S1 **Table of 604 orthologous proteins from 12 mesophilic Haloarchaea and** ***H. lacusprofundi*** **.**
(DOC)

Table S2 **Genome-wide tally and percent of amino acid substitutions between invariant mesophilic haloarchaeal proteins (vertical) and** ***H. lacusprofundi*** **(horizontal) for 604 selected cHOGs.**
(DOC)
