# Correction: Amino Acid Substitutions in Cold-Adapted Proteins from *Halorubrum lacusprofundi*, an Extremely Halophilic Microbe from Antarctica

**DOI:** 10.1371/journal.pone.0314323

**Published:** 2024-11-19

**Authors:** The PLOS ONE Staff

The initials of the first and fourth authors are indexed incorrectly in PubMed. The correct initials are, respectively: DasSarma S; and DasSarma P.

The correct citation is: DasSarma S, Capes MD, Karan R, DasSarma P (2013) Amino Acid Substitutions in Cold-Adapted Proteins from *Halorubrum lacusprofundi*, an Extremely Halophilic Microbe from Antarctica. PLoS ONE 8(3): e58587. https://doi.org/10.1371/journal.pone.0058587
