# Supplementary material for: Amino Acid Substitutions in Cold-Adapted Proteins from Halorubrum lacusprofundi, an Extremely Halophilic Microbe from Antarctica
Source: PLoS One. 2013 Mar 11;8(3):e58587. doi: 10.1371/journal.pone.0058587 (PMC3594186; doi:10.1371/journal.pone.0058587)
Supplement: Table S1 — Table of 604 orthologous proteins from 12 mesophilic Haloarchaea and H. lacusprofundi . (DOC) [file pone.0058587.s001.doc]

Table S1. Table of 604 orthologous proteins from 12 mesophilic Haloarchaea and *H. lacusprofundi*.

| ***cHOG*** | ***H.NRC-1*** | ***H.marismortui*** | ***H.volcanii*** | ***H.borinquense*** | ***H.mukohataei*** | ***H.utahensis*** | ***N.pharaonis*** | ***H.walsbyi*** | ***H.xanaduensis*** | ***N.magadii*** | ***H.jeotgali*** | ***H.turkmenica*** | | ***H.lacusprofundi*** | |
| --- | --- | --- | --- | --- | --- | --- | --- | --- | --- | --- | --- | --- | --- | --- | --- |
| cHOG0143 | VNG1111 | RRNAC1411 | HVO_0911 | hbor_23160 | hmuk_1945 | huta_0250 | NP4456A | HQ1607A | GI-335339525 | GI-289530414 | GI-299124751 | 2502018063 | HLAC0782 | |  |
| cHOG0144 | VNG0504 | RRNAC0269 | HVO_1608 | hbor_16130 | hmuk_2814 | huta_2275 | NP1668A | HQ2628A | GI-335339607 | GI-289529881 | GI-299125500 | 2502017595 | HLAC0779 | |  |
| cHOG0145 | VNG2595 | RRNAC2374 | HVO_1925 | hbor_05240 | hmuk_0546 | huta_1159 | NP0584A | HQ3140A | GI-335338794 | GI-289530796 | GI-299125711 | 2502019202 | HLAC2297 | |  |
| cHOG0146 | VNG2441 | RRNAC2719 | HVO_0039 | hbor_01810 | hmuk_0823 | huta_1416 | NP1306A | HQ3720A | GI-335336761 | GI-289530992 | GI-299123257 | 2502015927 | HLAC2627 | |  |
| cHOG0147 | VNG2476 | RRNAC2901 | HVO_0099 | hbor_01230 | hmuk_1203 | huta_1013 | NP0898A | HQ3436A | GI-335336925 | GI-289531250 | GI-299123318 | 2502015791 | HLAC2213 | |  |
| cHOG0148 | VNG1500 | RRNAC0946 | HVO_2768 | hbor_12400 | hmuk_2105 | huta_0680 | NP2946A | HQ2931A | GI-335337844 | GI-289530295 | GI-299124873 | 2502018562 | HLAC1696 | |  |
| cHOG0150 | VNG0494 | RRNAC3340 | HVO_1592 | hbor_16230 | hmuk_2845 | huta_0309 | NP0216A | HQ2639A | GI-335339571 | GI-289529892 | GI-299125540 | 2502017639 | HLAC1291 | |  |
| cHOG0152 | VNG2001 | RRNAC3189 | HVO_1847 | hbor_04480 | hmuk_1306 | huta_1161 | NP5040A | HQ1110A | GI-335337230 | GI-289530589 | GI-299123963 | 2502019012 | HLAC2686 | |  |
| cHOG0153 | VNG0782 | RRNAC1443 | HVO_1020 | hbor_21990 | hmuk_1905 | huta_0719 | NP3228A | HQ1584A | GI-335338244 | GI-289531636 | GI-299125206 | 2502017124 | HLAC2402 | |  |
| cHOG0154 | VNG1697 | RRNAC1605 | HVO_2558 | hbor_10290 | hmuk_1834 | huta_2298 | NP4866A | HQ2836A | GI-335336423 | GI-289529457 | GI-299124064 | 2502017854 | HLAC2443 | |  |
| cHOG0155 | VNG0527 | RRNAC1828 | HVO_0861 | hbor_23580 | hmuk_2920 | huta_0378 | NP1500A | HQ1708A | GI-335338249 | GI-289531640 | GI-299125474 | 2502017119 | HLAC0174 | |  |
| cHOG0156 | VNG0491 | RRNAC3339 | HVO_1590 | hbor_16250 | hmuk_2846 | huta_0310 | NP0218A | HQ2640A | GI-335339572 | GI-289529807 | GI-299125542 | 2502017638 | HLAC0682 | |  |
| cHOG0157 | VNG2155 | RRNAC2733 | HVO_0300 | hbor_28710 | hmuk_0936 | huta_1246 | NP5164A | HQ3257A | GI-335337209 | GI-289530611 | GI-299123419 | 2502018984 | HLAC0312 | |  |
| cHOG0158 | VNG2240 | RRNAC2487 | HVO_0145 | hbor_00010 | hmuk_1247 | huta_1957 | NP1068A | HQ3405A | GI-335336643 | GI-289531090 | GI-299123481 | 2502015641 | HLAC2519 | |  |
| cHOG0159 | VNG0475 | RRNAC0717 | HVO_0889 | hbor_23320 | hmuk_3086 | huta_0896 | NP1230A | HQ1537A | GI-335339594 | GI-289529787 | GI-299125923 | 2502017612 | HLAC0929 | |  |
| cHOG0160 | VNG2504 | RRNAC2966 | HVO_2948 | hbor_03090 | hmuk_0664 | huta_1675 | NP0696A | HQ1049A | GI-335336546 | GI-289530921 | GI-299125237 | 2502019274 | HLAC2671 | |  |
| cHOG0161 | VNG0133 | RRNAC2302 | HVO_0519 | hbor_26830 | hmuk_0565 | huta_2247 | NP0314A | HQ1435A | GI-335337172 | GI-289531420 | GI-299125984 | 2502016011 | HLAC0111 | |  |
| cHOG0162 | VNG0324 | RRNAC0546 | HVO_0806 | hbor_24110 | hmuk_2510 | huta_1475 | NP1746A | HQ1573A | GI-335339274 | GI-289531813 | GI-299125671 | 2502016454 | HLAC0562 | |  |
| cHOG0163 | VNG1542 | RRNAC0474 | HVO_2464 | hbor_10970 | hmuk_2713 | huta_0439 | NP4354A | HQ2857A | GI-335336276 | GI-289529970 | GI-299124422 | 2502017686 | HLAC2208 | |  |
| cHOG0167 | VNG1306 | RRNAC1097 | HVO_2808 | hbor_12870 | hmuk_2079 | huta_2832 | NP4264A | HQ2994A | GI-335337953 | GI-289532329 | GI-299124817 | 2502016547 | HLAC1990 | |  |
| cHOG0168 | VNG1014 | RRNAC0540 | HVO_1302 | hbor_19230 | hmuk_2368 | huta_2776 | NP2918A | HQ3359A | GI-335338107 | GI-289529864 | GI-299124888 | 2502017726 | HLAC2466 | |  |
| cHOG0169 | VNG1829 | RRNAC3472 | HVO_2625 | hbor_09490 | hmuk_1394 | huta_0426 | NP1970A | HQ3194A | GI-335339004 | GI-289531923 | GI-299124318 | 2502016371 | HLAC2593 | |  |
| cHOG0170 | VNG2073 | RRNAC2810 | HVO_1918 | hbor_05170 | hmuk_0740 | huta_1319 | NP1688A | HQ3137A | GI-335337138 | GI-289531401 | GI-299123883 | 2502015970 | HLAC0007 | |  |
| cHOG0171 | VNG1503 | RRNAC0963 | HVO_2763 | hbor_12310 | hmuk_2119 | huta_2921 | NP4346A | HQ2927A | GI-335337825 | GI-289530092 | GI-299124869 | 2502018509 | HLAC1860 | |  |
| cHOG0172 | VNG1160 | RRNAC0106 | HVO_2740 | hbor_12090 | hmuk_2624 | huta_0753 | NP3666A | HQ2882A | GI-335336114 | GI-289529691 | GI-299124776 | 2502018161 | HLAC1845 | |  |
| cHOG0175 | VNG2533 | RRNAC2232 | HVO_1816 | hbor_03960 | hmuk_0212 | huta_0970 | NP1706A | HQ1061A | GI-299126013 | GI-335338409 | GI-289532200 | 2502014165 | HLAC2077 | |  |
| cHOG0176 | VNG0159 | RRNAC2550 | HVO_0551 | hbor_26600 | hmuk_0376 | huta_0007 | NP0536A | HQ1456A | GI-335337496 | GI-289530518 | GI-299125919 | 2502018864 | HLAC0629 | |  |
| cHOG0177 | VNG1830 | RRNAC3471 | HVO_2624 | hbor_09500 | hmuk_1393 | huta_0427 | NP1968A | HQ3195A | GI-335339003 | GI-289531922 | GI-299124319 | 2502016370 | HLAC2594 | |  |
| cHOG0179 | VNG2147 | RRNAC3151 | HVO_0309 | hbor_28630 | hmuk_1274 | huta_1430 | NP5034A | HQ3251A | GI-335337214 | GI-289530606 | GI-299123430 | 2502018992 | HLAC0391 | |  |
| cHOG0180 | VNG2398 | RRNAC2440 | HVO_0019 | hbor_02020 | hmuk_1127 | huta_2258 | NP0956A | HQ3733A | GI-335336807 | GI-289531142 | GI-299123180 | 2502019424 | HLAC2736 | |  |
| cHOG0181 | VNG0294 | RRNAC1812 | HVO_0773 | hbor_24390 | hmuk_3206 | huta_2905 | NP1178A | HQ1168A | GI-335339395 | GI-289532034 | GI-299125891 | 2502016921 | HLAC0551 | |  |
| cHOG0182 | VNG0184 | RRNAC2014 | HVO_0574 | hbor_26310 | hmuk_0182 | huta_2403 | NP5122A | HQ1416A | GI-335337639 | GI-289530205 | GI-299125586 | 2502018631 | HLAC0529 | |  |
| cHOG0183 | VNG2154 | RRNAC2735 | HVO_0302 | hbor_28700 | hmuk_0948 | huta_1833 | NP5162A | HQ3256A | GI-335337207 | GI-289530612 | GI-299123420 | 2502018982 | HLAC0314 | |  |
| cHOG0184 | VNG2005 | RRNAC3183 | HVO_1854 | hbor_04550 | hmuk_1302 | huta_1986 | NP5044A | HQ1107A | GI-335338835 | GI-289530747 | GI-299123903 | 2502019135 | HLAC2219 | |  |
| cHOG0185 | VNG1785 | RRNAC1726 | HVO_2324 | hbor_07370 | hmuk_1541 | huta_0201 | NP4534A | HQ3326A | GI-335337498 | GI-289530463 | GI-299123889 | 2502018905 | HLAC2133 | |  |
| cHOG0186 | VNG1572 | RRNAC1101 | HVO_0590 | hbor_26140 | hmuk_2245 | huta_2498 | NP0736A | HQ1408A | GI-335337619 | GI-289530223 | GI-299123216 | 2502018479 | HLAC0230 | |  |
| cHOG0187 | VNG0646 | RRNAC1455 | HVO_0986 | hbor_21390 | hmuk_1925 | huta_0694 | NP2310A | HQ1646A | GI-335339210 | GI-289532446 | GI-299125375 | 2502016723 | HLAC0712 | |  |
| cHOG0188 | VNG2238 | RRNAC2460 | HVO_0140 | hbor_29620 | hmuk_0706 | huta_2183 | NP1264A | HQ3402A | GI-335336638 | GI-289531093 | GI-299123801 | 2502015644 | HLAC2504 | |  |
| cHOG0189 | VNG1235 | RRNAC0512 | HVO_1312 | hbor_19130 | hmuk_2357 | huta_0273 | NP3358A | HQ3352A | GI-335338214 | GI-289531602 | GI-299124657 | 2502017041 | HLAC1779 | |  |
| cHOG0190 | VNG1988 | RRNAC3214 | HVO_1975 | hbor_05710 | hmuk_1321 | huta_2688 | NP2524A | HQ3098A | GI-335338991 | GI-289531913 | GI-299123976 | 2502016355 | HLAC1963 | |  |
| cHOG0192 | VNG1033 | RRNAC0529 | HVO_1295 | hbor_19300 | hmuk_2375 | huta_0367 | NP2140A | HQ3364A | GI-335337884 | GI-289529842 | GI-299124532 | 2502017524 | HLAC1343 | |  |
| cHOG0194 | VNG0821 | RRNAC0542 | HVO_1032 | hbor_22130 | hmuk_2566 | huta_2530 | NP3110A | HQ1601A | GI-335339083 | GI-289532423 | GI-299125252 | 2502016679 | HLAC1030 | |  |
| cHOG0195 | VNG2144 | RRNAC3154 | HVO_0311 | hbor_28610 | hmuk_1276 | huta_1432 | NP1020A | HQ3249A | GI-335337216 | GI-289530604 | GI-299123432 | 2502018994 | HLAC0276 | |  |
| cHOG0196g | VNG1236 | RRNAC0513 | HVO_1313 | hbor_19120 | hmuk_2356 | huta_0272 | NP3360A | HQ3351A | GI-335338215 | GI-289531603 | GI-299124656 | 2502017040 | HLAC1780 | |  |
| cHOG0198 | VNG1370 | RRNAC3374 | HVO_1111 | hbor_22990 | hmuk_2792 | huta_2493 | NP3816A | HQ1627A | GI-335338720 | GI-289532805 | GI-299124686 | 2502017451 | HLAC0881 | |  |
| cHOG0199 | VNG2283 | RRNAC2561 | HVO_0206 | hbor_00600 | hmuk_1124 | huta_1929 | NP0710A | HQ3670A | GI-335336783 | GI-289531128 | GI-299123289 | 2502015623 | HLAC0719 | |  |
| cHOG0200 | VNG0303 | RRNAC1914 | HVO_0783 | hbor_24330 | hmuk_3199 | huta_2897 | NP0910A | HQ1163A | GI-335339391 | GI-289532025 | GI-299125898 | 2502016470 | HLAC1038 | |  |
| cHOG0202 | VNG1716 | RRNAC1591 | HVO_2543 | hbor_10440 | hmuk_1849 | huta_2313 | NP4896A | HQ2821A | GI-335336408 | GI-289529472 | GI-299124079 | 2502017869 | HLAC2428 | |  |
| cHOG0203 | VNG1142 | RRNAC0069 | HVO_2774 | hbor_12470 | hmuk_2598 | huta_2515 | NP2846A | HQ2935A | GI-335336135 | GI-289529666 | GI-299124788 | 2502018182 | HLAC1825 | |  |
| cHOG0204 | VNG2045 | RRNAC2490 | HVO_1895 | hbor_05000 | hmuk_1173 | huta_1954 | NP5070A | HQ1341A | GI-335337481 | GI-289530502 | GI-299123944 | 2502018698 | HLAC2392 | |  |
| cHOG0205 | VNG2249 | RRNAC2837 | HVO_0165 | hbor_00120 | hmuk_0754 | huta_1515 | NP0972A | HQ3413A | GI-335336658 | GI-289531073 | GI-299123472 | 2502015735 | HLAC0061 | |  |
| cHOG0206 | VNG1444 | RRNAC0272 | HVO_2675 | hbor_11320 | hmuk_2450 | huta_0450 | NP2876A | HQ2720A | GI-335338761 | GI-289529272 | GI-299124464 | 2502017489 | HLAC2478 | |  |
| cHOG0207 | VNG1148 | RRNAC0078 | HVO_2762 | hbor_12300 | hmuk_2605 | huta_2046 | NP2852A | HQ2926A | GI-335336130 | GI-289529677 | GI-299124785 | 2502018177 | HLAC1830 | |  |
| cHOG0208 | VNG2276 | RRNAC2568 | HVO_0199 | hbor_00530 | hmuk_1140 | huta_0077 | NP4676A | HQ3673A | GI-335336844 | GI-289531175 | GI-299123279 | 2502019402 | HLAC2655 | |  |
| cHOG0209 | VNG1165 | RRNAC0257 | HVO_2746 | hbor_12140 | hmuk_2671 | huta_0621 | NP3678A | HQ2878A | GI-335339436 | GI-289530327 | GI-299124769 | 2502018025 | HLAC2104 | |  |
| cHOG0210 | VNG1844 | RRNAC3442 | HVO_2511 | hbor_09030 | hmuk_1652 | huta_3003 | NP2492A | HQ2776A | GI-335337062 | GI-289532174 | GI-299124338 | 2502016193 | HLAC2140 | |  |
| cHOG0211 | VNG0876 | RRNAC0438 | HVO_1557 | hbor_16720 | hmuk_2746 | huta_0433 | NP3940A | HQ2665A | GI-335338599 | GI-289532692 | GI-299125200 | 2502017244 | HLAC0862 | |  |
| cHOG0212 | VNG2646 | RRNAC2087 | HVO_0390 | hbor_28020 | hmuk_0427 | huta_0141 | NP0340A | HQ3178A | GI-335337441 | GI-289530707 | GI-299126066 | 2502018742 | HLAC0035 | |  |
| cHOG0213 | VNG1232 | RRNAC0127 | HVO_1308 | hbor_19170 | hmuk_2641 | huta_0495 | NP3078A | HQ3358A | GI-335338205 | GI-289531599 | GI-299124664 | 2502017046 | HLAC0687 | |  |
| cHOG0214 | VNG2419 | RRNAC0651 | HVO_1239 | hbor_19920 | hmuk_1882 | huta_1046 | NP0888A | HQ2231A | GI-335336631 | GI-289531101 | GI-299123235 | 2502015652 | HLAC1778 | |  |
| cHOG0215 | VNG2267 | RRNAC2857 | HVO_0185 | hbor_00390 | hmuk_0997 | huta_1033 | NP1350A | HQ3684A | GI-335336943 | GI-289531269 | GI-299123452 | 2502015757 | HLAC2608 | |  |
| cHOG0216 | VNG1163 | RRNAC0256 | HVO_2744 | hbor_12120 | hmuk_2670 | huta_0620 | NP3674A | HQ2879A | GI-335339434 | GI-289530325 | GI-299124771 | 2502018027 | HLAC2106 | |  |
| cHOG0217 | VNG2608 | RRNAC2106 | HVO_0431 | hbor_27640 | hmuk_0305 | huta_0328 | NP0770A | HQ1376A | GI-335338807 | GI-289530780 | GI-299125721 | 2502019145 | HLAC0335 | |  |
| cHOG0218 | VNG1103 | RRNAC1418 | HVO_2755 | hbor_12230 | hmuk_2184 | huta_0256 | NP4448A | HQ2901A | GI-335338622 | GI-289532719 | GI-299124758 | 2502017136 | HLAC2533 | |  |
| cHOG0219 | VNG1253 | RRNAC1135 | HVO_1337 | hbor_18980 | hmuk_2419 | huta_0545 | NP3962A | HQ2404A | GI-335338066 | GI-289532284 | GI-299124928 | 2502014192 | HLAC0624 | |  |
| cHOG0220 | VNG1369 | RRNAC3375 | HVO_1110 | hbor_22980 | hmuk_2791 | huta_2494 | NP3814A | HQ1628A | GI-335338719 | GI-289531060 | GI-299124687 | 2502017450 | HLAC0880 | |  |
| cHOG0222 | VNG1647 | RRNAC1518 | HVO_2454 | hbor_10770 | hmuk_1769 | huta_1283 | NP3342A | HQ3167A | GI-335336481 | GI-289529310 | GI-299123822 | 2502017767 | HLAC1941 | |  |
| cHOG0223 | VNG2269 | RRNAC2858 | HVO_0186 | hbor_00400 | hmuk_0998 | huta_1032 | NP1348A | HQ3683A | GI-335336944 | GI-289531270 | GI-299123451 | 2502015758 | HLAC2609 | |  |
| cHOG0224 | VNG0096 | RRNAC2261 | HVO_0477 | hbor_26980 | hmuk_0528 | huta_2557 | NP0014A | HQ1359A | GI-335337038 | GI-289531360 | GI-299125949 | 2502015935 | HLAC1671 | |  |
| cHOG0225 | VNG2332 | RRNAC3088 | HVO_0076 | hbor_01450 | hmuk_1103 | huta_0928 | NP1330A | HQ3452A | GI-335336905 | GI-289531231 | GI-299123162 | 2502019329 | HLAC2254 | |  |
| cHOG0226 | VNG2270 | RRNAC2861 | HVO_0191 | hbor_00420 | hmuk_1006 | huta_1024 | NP0586A | HQ3681A | GI-335336968 | GI-289531282 | GI-299123269 | 2502019283 | HLAC2604 | |  |
| cHOG0227 | VNG2520 | RRNAC2220 | HVO_1823 | hbor_03840 | hmuk_0224 | huta_0965 | NP1430A | HQ1066A | GI-335338875 | GI-289530535 | GI-299126018 | 2502019073 | HLAC2164 | |  |
| cHOG0228 | VNG1074 | RRNAC0844 | HVO_1461 | hbor_17700 | hmuk_2249 | huta_0393 | NP2736A | HQ1876A | GI-335338664 | GI-289532755 | GI-299124710 | 2502017395 | HLAC1174 | |  |
| cHOG0229 | VNG0748 | RRNAC1068 | HVO_2848 | hbor_13590 | hmuk_2232 | huta_0411 | NP3446A | HQ3002A | GI-335338333 | GI-289532567 | GI-299124829 | 2502017369 | HLAC0031 | |  |
| cHOG0230 | VNG1104 | RRNAC1417 | HVO_2756 | hbor_12240 | hmuk_2185 | huta_0255 | NP4450A | HQ2902A | GI-335338621 | GI-289532718 | GI-299124757 | 2502017135 | HLAC2534 | |  |
| cHOG0232 | VNG2436 | RRNAC2681 | HVO_0048 | hbor_01710 | hmuk_0770 | huta_1504 | NP5254A | HQ3712A | GI-335336770 | GI-289530986 | GI-299123254 | 2502015922 | HLAC2618 | |  |
| cHOG0233 | VNG2141 | RRNAC3157 | HVO_0314 | hbor_28580 | hmuk_1279 | huta_1435 | NP1026A | HQ3246A | GI-335337219 | GI-289530601 | GI-299123435 | 2502018997 | HLAC0279 | |  |
| cHOG0234 | VNG2411 | RRNAC2711 | HVO_0001 | hbor_02110 | hmuk_0815 | huta_1613 | NP0596A | HQ1001A | GI-335336813 | GI-289531150 | GI-299123187 | 2502015591 | HLAC0001 | |  |
| cHOG0235 | VNG1073 | RRNAC0843 | HVO_1375 | hbor_18630 | hmuk_2248 | huta_0392 | NP2738A | HQ2402A | GI-335338663 | GI-289532754 | GI-299124709 | 2502017394 | HLAC1497 | |  |
| cHOG0236 | VNG2521 | RRNAC2221 | HVO_1822 | hbor_03850 | hmuk_0223 | huta_0966 | NP1718A | HQ1065A | GI-335338874 | GI-289530533 | GI-299126017 | 2502019074 | HLAC1930 | |  |
| cHOG0237 | VNG0283 | RRNAC1867 | HVO_0766 | hbor_24480 | hmuk_3021 | huta_1192 | NP5318A | HQ1177A | GI-335339404 | GI-289531971 | GI-299125879 | 2502016909 | HLAC0637 | |  |
| cHOG0238 | VNG1874 | RRNAC3414 | HVO_2584 | hbor_09950 | hmuk_1502 | huta_0279 | NP2428A | HQ3214A | GI-335337244 | GI-289532133 | GI-299124366 | 2502016148 | HLAC2306 | |  |
| cHOG0239 | VNG1939 | RRNAC3275 | HVO_2193 | hbor_06310 | hmuk_1420 | huta_2216 | NP4996A | HQ3128A | GI-335337088 | GI-289531848 | GI-299124031 | 2502016257 | HLAC1700 | |  |
| cHOG0241 | VNG1690 | RRNAC1610 | HVO_2563 | hbor_10240 | hmuk_1829 | huta_2293 | NP4856A | HQ2841A | GI-335336428 | GI-289529452 | GI-299124059 | 2502017849 | HLAC2448 | |  |
| cHOG0242 | VNG1150 | RRNAC0081 | HVO_2725 | hbor_11930 | hmuk_2608 | huta_2049 | NP3696A | HQ2889A | GI-335336127 | GI-289529680 | GI-299124783 | 2502018175 | HLAC2048 | |  |
| cHOG0243 | VNG2410 | RRNAC2710 | HVO_3014 | hbor_02100 | hmuk_0814 | huta_1615 | NP0594A | HQ3738A | GI-335336812 | GI-289531149 | GI-299123186 | 2502019431 | HLAC2746 | |  |
| cHOG0244 | VNG2271 | RRNAC2862 | HVO_0194 | hbor_00440 | hmuk_1003 | huta_1055 | NP0588A | HQ3680A | GI-335336973 | GI-289531284 | GI-299123271 | 2502019286 | HLAC2631 | |  |
| cHOG0245 | VNG2280 | RRNAC2565 | HVO_0203 | hbor_00570 | hmuk_1126 | huta_1927 | NP0900A | HQ3671A | GI-335336785 | GI-289531130 | GI-299123284 | 2502015617 | HLAC0384 | |  |
| cHOG0246 | VNG2062 | RRNAC2818 | HVO_1907 | hbor_05130 | hmuk_0735 | huta_2215 | NP2252A | HQ3104A | GI-335338871 | GI-289530530 | GI-299123853 | 2502019077 | HLAC1958 | |  |
| cHOG0247 | VNG2452 | RRNAC3145 | HVO_0128 | hbor_00950 | hmuk_1265 | huta_2091 | NP0870A | HQ3047A | GI-335336727 | GI-289531022 | GI-299123345 | 2502015856 | HLAC1857 | |  |
| cHOG0248 | VNG2473 | RRNAC2910 | HVO_0104 | hbor_01160 | hmuk_1185 | huta_1273 | NP0878A | HQ3429A | GI-335336672 | GI-289531054 | GI-299123324 | 2502015801 | HLAC2624 | |  |
| cHOG0249 | VNG0081 | RRNAC1186 | HVO_1864 | hbor_04650 | hmuk_0919 | huta_0039 | NP2274A | HQ1099A | GI-335338843 | GI-289530737 | GI-299123911 | 2502019127 | HLAC2386 | |  |
| cHOG0250 | VNG1097 | RRNAC1292 | HVO_1125 | hbor_21180 | hmuk_2181 | huta_0052 | NP4440A | HQ1654A | GI-335338715 | GI-289532799 | GI-299124737 | 2502017445 | HLAC0291 | |  |
| cHOG0252 | VNG2031 | RRNAC2486 | HVO_1878 | hbor_04860 | hmuk_1161 | huta_1352 | NP3828A | HQ1091A | GI-335337047 | GI-289532048 | GI-299123925 | 2502016078 | HLAC0090 | |  |
| cHOG0253 | VNG2006 | RRNAC3181 | HVO_1856 | hbor_04580 | hmuk_1301 | huta_1983 | NP4418A | HQ1106A | GI-335338836 | GI-289530743 | GI-299123905 | 2502019133 | HLAC2217 | |  |
| cHOG0255 | VNG2636 | RRNAC2116 | HVO_0393 | hbor_27990 | hmuk_0318 | huta_0314 | NP0504A | HQ3072A | GI-335337474 | GI-289530492 | GI-299125758 | 2502018737 | HLAC2607 | |  |
| cHOG0256 | VNG0549 | RRNAC3512 | HVO_0699 | hbor_25180 | hmuk_2880 | huta_2790 | NP1618A | HQ1255A | GI-335339295 | GI-289531711 | GI-299125459 | 2502016480 | HLAC1009 | |  |
| cHOG0257 | VNG0784 | RRNAC0788 | HVO_1143 | hbor_20980 | hmuk_2392 | huta_0720 | NP3230A | HQ2356A | GI-335338243 | GI-289531635 | GI-299125207 | 2502017125 | HLAC0607 | |  |
| cHOG0258 | VNG0905 | RRNAC0444 | HVO_1402 | hbor_18320 | hmuk_2740 | huta_1384 | NP3514A | HQ1522A | GI-335338143 | GI-289529748 | GI-299125254 | 2502018094 | HLAC1418 | |  |
| cHOG0259 | VNG2406 | RRNAC2708 | HVO_3012 | hbor_02080 | hmuk_0812 | huta_1617 | NP0590A | HQ3736A | GI-335336810 | GI-289531147 | GI-299123184 | 2502019429 | HLAC2744 | |  |
| cHOG0260 | VNG2462 | RRNAC3118 | HVO_0120 | hbor_01030 | hmuk_1229 | huta_1328 | NP0182A | HQ3419A | GI-335336695 | GI-289531038 | GI-299123339 | 2502015829 | HLAC0821 | |  |
| cHOG0261 | VNG1873 | RRNAC3419 | HVO_2588 | hbor_09930 | hmuk_1506 | huta_0281 | NP2430A | HQ3212A | GI-335337240 | GI-289532140 | GI-299124362 | 2502016156 | HLAC2330 | |  |
| cHOG0262 | VNG1984 | RRNAC3216 | HVO_1978 | hbor_05750 | hmuk_1323 | huta_0985 | NP2392A | HQ3096A | GI-335338989 | GI-289531910 | GI-299123978 | 2502016351 | HLAC1966 | |  |
| cHOG0263 | VNG0361 | RRNAC0675 | HVO_0727 | hbor_24880 | hmuk_2965 | huta_2451 | NP1156A | HQ1233A | GI-335339473 | GI-289530361 | GI-299125554 | 2502018271 | HLAC0599 | |  |
| cHOG0264 | VNG1511 | RRNAC0979 | HVO_2889 | hbor_14030 | hmuk_2195 | huta_2931 | NP2874A | HQ1797A | GI-335337799 | GI-289530109 | GI-299124859 | 2502018360 | HLAC1616 | |  |
| cHOG0265 | VNG2366 | RRNAC2705 | HVO_3008 | hbor_02290 | hmuk_0802 | huta_1099 | NP0338A | HQ3458A | GI-335336872 | GI-289531197 | GI-299123208 | 2502019378 | HLAC2246 | |  |
| cHOG0266 | VNG1774 | RRNAC1708 | HVO_2311 | hbor_07260 | hmuk_1612 | huta_2825 | NP4502A | HQ3336A | GI-335337511 | GI-289530450 | GI-299124274 | 2502018892 | HLAC2132 | |  |
| cHOG0268 | VNG0163 | RRNAC2532 | HVO_0552 | hbor_26580 | hmuk_0366 | huta_0493 | NP0538A | HQ1460A | GI-335337488 | GI-289530513 | GI-299125918 | 2502018870 | HLAC0119 | |  |
| cHOG0269 | VNG2356 | RRNAC2704 | HVO_3010 | hbor_02260 | hmuk_0800 | huta_1486 | NP0336A | HQ1020A | GI-335336874 | GI-289531200 | GI-299123213 | 2502019375 | HLAC2546 | |  |
| cHOG0270 | VNG1471 | RRNAC0293 | HVO_2698 | hbor_11520 | hmuk_2483 | huta_2752 | NP3992A | HQ2718A | GI-335336161 | GI-289529646 | GI-299124519 | 2502018204 | HLAC1797 | |  |
| cHOG0272 | VNG2338 | RRNAC2691 | HVO_0065 | hbor_01600 | hmuk_0763 | huta_1619 | NP0476A | HQ3461A | GI-335336893 | GI-289531215 | GI-299123173 | 2502019342 | HLAC0150 | |  |
| cHOG0273 | VNG2420 | RRNAC3064 | HVO_2998 | hbor_02450 | hmuk_1097 | huta_2620 | NP0282A | HQ3700A | GI-335336566 | GI-289531105 | GI-299123237 | 2502015657 | HLAC2550 | |  |
| cHOG0274 | VNG1703 | RRNAC1600 | HVO_2552 | hbor_10350 | hmuk_1840 | huta_2304 | NP4878A | HQ2830A | GI-335336417 | GI-289529463 | GI-299124070 | 2502017860 | HLAC2437 | |  |
| cHOG0275 | VNG0507 | RRNAC0234 | HVO_1610 | hbor_16110 | hmuk_2812 | huta_1484 | NP1670A | HQ2627A | GI-335339673 | GI-289529718 | GI-299125490 | 2502018120 | HLAC1784 | |  |
| cHOG0276g | VNG2524 | RRNAC2224 | HVO_1819 | hbor_03880 | hmuk_0220 | huta_0969 | NP1708A | HQ1062A | GI-335337147 | GI-289531333 | GI-299126014 | 2502015984 | HLAC1909 | |  |
| cHOG0277 | VNG1711 | RRNAC1595 | HVO_2547 | hbor_10400 | hmuk_1845 | huta_2309 | NP4888A | HQ2825A | GI-335336412 | GI-289529468 | GI-299124075 | 2502017865 | HLAC2432 | |  |
| cHOG0279 | VNG1365 | RRNAC0039 | HVO_1107 | hbor_22950 | hmuk_2788 | huta_2507 | NP3808A | HQ1505A | GI-335338716 | GI-289532801 | GI-299124690 | 2502017447 | HLAC0688 | |  |
| cHOG0280 | VNG0125 | RRNAC2308 | HVO_0508 | hbor_27260 | hmuk_0579 | huta_1830 | NP0412A | HQ1137A | GI-335337192 | GI-289531459 | GI-299125974 | 2502016036 | HLAC0081 | |  |
| cHOG0281 | VNG1083 | RRNAC0837 | HVO_1470 | hbor_17630 | hmuk_0041 | huta_0814 | NP2724A | HQ1872A | GI-335338673 | GI-289532769 | GI-299124716 | 2502017404 | HLAC1487 | |  |
| cHOG0282 | VNG1287 | RRNAC1178 | HVO_1249 | hbor_19800 | hmuk_2396 | huta_0525 | NP3528A | HQ1808A | GI-335339655 | GI-289529823 | GI-299124642 | 2502017558 | HLAC0947 | |  |
| cHOG0283 | VNG2454 | RRNAC3144 | HVO_0127 | hbor_00960 | hmuk_1264 | huta_2090 | NP0868A | HQ3046A | GI-335336728 | GI-289531023 | GI-299123344 | 2502015855 | HLAC1856 | |  |
| cHOG0284 | VNG0838 | RRNAC0375 | HVO_1620 | hbor_16030 | hmuk_2505 | huta_0154 | NP3640A | HQ2614A | GI-335338699 | GI-289532791 | GI-299124985 | 2502017428 | HLAC1216 | |  |
| cHOG0285 | VNG0394 | RRNAC1820 | HVO_0880 | hbor_23410 | hmuk_3079 | huta_2705 | NP4058A | HQ1530A | GI-335339279 | GI-289531821 | GI-299125583 | 2502016965 | HLAC0452 | |  |
| cHOG0286 | VNG2381 | RRNAC3041 | HVO_3006 | hbor_02350 | hmuk_1072 | huta_2640 | NP1086A | HQ3694A | GI-335336984 | GI-289531296 | GI-299123220 | 2502019291 | HLAC2725 | |  |
| cHOG0289 | VNG2190 | RRNAC2634 | HVO_1547 | hbor_16810 | hmuk_1055 | huta_1825 | NP0610A | HQ2671A | GI-335336613 | GI-289530967 | GI-299123108 | 2502015679 | HLAC0887 | |  |
| cHOG0290 | VNG2019 | RRNAC3170 | HVO_1870 | hbor_04720 | hmuk_1292 | huta_1973 | NP2268A | HQ1095A | GI-335338847 | GI-289530733 | GI-299123915 | 2502019123 | HLAC1683 | |  |
| cHOG0291 | VNG0378 | RRNAC0704 | HVO_0716 | hbor_25010 | hmuk_2934 | huta_2476 | NP0808A | HQ1244A | GI-335339331 | GI-289531985 | GI-299125574 | 2502016988 | HLAC0902 | |  |
| cHOG0292 | VNG0090 | RRNAC1298 | HVO_2304 | hbor_07190 | hmuk_2501 | huta_0037 | NP1440A | HQ3345A | GI-335337520 | GI-289530444 | GI-299124268 | 2502018745 | HLAC2122 | |  |
| cHOG0293 | VNG2139 | RRNAC3159 | HVO_0316 | hbor_28560 | hmuk_1281 | huta_1437 | NP1030A | HQ3244A | GI-335337221 | GI-289530599 | GI-299123437 | 2502018999 | HLAC0281 | |  |
| cHOG0294 | VNG1470 | RRNAC0292 | HVO_2697 | hbor_11510 | hmuk_2482 | huta_2753 | NP3990A | HQ2719A | GI-335336160 | GI-289529647 | GI-299124518 | 2502018203 | HLAC1798 | |  |
| cHOG0295 | VNG0332 | RRNAC1794 | HVO_0696 | hbor_25220 | hmuk_2998 | huta_2742 | NP1202A | HQ1259A | GI-335339265 | GI-289531802 | GI-299125665 | 2502016947 | HLAC0905 | |  |
| cHOG0299g | VNG2315 | RRNAC2604 | HVO_0219 | hbor_00710 | hmuk_0934 | huta_1545 | NP1392A | HQ3663A | GI-335336564 | GI-289530933 | GI-299123139 | 2502015633 | HLAC0497 | |  |
| cHOG0301 | VNG2310 | RRNAC2599 | HVO_0251 | hbor_29390 | hmuk_0886 | huta_2107 | NP1416A | HQ3706A | GI-335336592 | GI-289530937 | GI-299123871 | 2502015638 | HLAC0406 | |  |
| cHOG0302 | VNG2142 | RRNAC3156 | HVO_0313 | hbor_28590 | hmuk_1278 | huta_1434 | NP1024A | HQ3247A | GI-335337218 | GI-289530602 | GI-299123434 | 2502018996 | HLAC0278 | |  |
| cHOG0303 | VNG0137 | RRNAC2298 | HVO_0521 | hbor_26800 | hmuk_0562 | huta_2250 | NP0320A | HQ1438A | GI-335337175 | GI-289531424 | GI-299125988 | 2502016014 | HLAC0336 | |  |
| cHOG0304 | VNG0387 | RRNAC0711 | HVO_0708 | hbor_25080 | hmuk_2928 | huta_2483 | NP0798A | HQ1247A | GI-335339339 | GI-289531997 | GI-299123450 | 2502016980 | HLAC0461 | |  |
| cHOG0306 | VNG2072 | RRNAC2808 | HVO_1921 | hbor_05200 | hmuk_0744 | huta_1764 | NP1692A | HQ3138A | GI-335337131 | GI-289531394 | GI-299123884 | 2502015961 | HLAC2073 | |  |
| cHOG0307 | VNG2207 | RRNAC2523 | HVO_A0498 | hbor_02540 | hmuk_0853 | huta_1782 | NP5156A | HQ1023A | GI-335336604 | GI-289531116 | GI-299123103 | 2502015670 | HLAC2181 | |  |
| cHOG0308 | VNG2647 | RRNAC2072 | HVO_0388 | hbor_28040 | hmuk_0272 | huta_0142 | NP0874A | HQ3180A | GI-335337445 | GI-289530524 | GI-299126069 | 2502018740 | HLAC0359 | |  |
| cHOG0309 | VNG1352 | RRNAC0023 | HVO_2902 | hbor_14270 | hmuk_2776 | huta_2658 | NP3774A | HQ2468A | GI-335338723 | GI-289532807 | GI-299124562 | 2502017457 | HLAC1472 | |  |
| cHOG0310 | VNG1548 | RRNAC3501 | HVO_0815 | hbor_24020 | hmuk_2060 | huta_2797 | NP1628A | HQ1568A | GI-335339292 | GI-289531715 | GI-299123219 | 2502016924 | HLAC0023 | |  |
| cHOG0311 | VNG1997 | RRNAC3203 | HVO_1963 | hbor_05590 | hmuk_1312 | huta_2679 | NP4982A | HQ3074A | GI-335338998 | GI-289531920 | GI-299123968 | 2502016365 | HLAC2060 | |  |
| cHOG0312 | VNG2468 | RRNAC3108 | HVO_0112 | hbor_01120 | hmuk_1240 | huta_1003 | NP0994A | HQ3425A | GI-335336684 | GI-289531049 | GI-299123332 | 2502015816 | HLAC2688 | |  |
| cHOG0313g | VNG2157 | RRNAC2740 | HVO_0298 | hbor_28750 | hmuk_1021 | huta_1622 | NP1298A | HQ3259A | GI-335337202 | GI-289530618 | GI-299123418 | 2502018976 | HLAC2542 | |  |
| cHOG0314 | VNG1255 | RRNAC1133 | HVO_1338 | hbor_18970 | hmuk_2418 | huta_0544 | NP3960A | HQ2405A | GI-335338065 | GI-289532283 | GI-299124929 | 2502014193 | HLAC0623 | |  |
| cHOG0316 | VNG1648 | RRNAC1519 | HVO_2455 | hbor_10780 | hmuk_1770 | huta_1282 | NP3340A | HQ3168A | GI-335336480 | GI-289529311 | GI-299123823 | 2502017768 | HLAC1940 | |  |
| cHOG0317 | VNG2204 | RRNAC2629 | HVO_0234 | hbor_29560 | hmuk_0912 | huta_1543 | NP5160A | HQ3649A | GI-335336608 | GI-289530964 | GI-299123106 | 2502015675 | HLAC1670 | |  |
| cHOG0318 | VNG2459 | RRNAC3124 | HVO_0123 | hbor_01000 | hmuk_1226 | huta_1261 | NP0096A | HQ3418A | GI-335336730 | GI-289531025 | GI-299123343 | 2502015850 | HLAC0496 | |  |
| cHOG0319 | VNG2222 | RRNAC2950 | HVO_0449 | hbor_27460 | hmuk_0983 | huta_1264 | NP0106A | HQ1385A | GI-335336555 | GI-289530927 | GI-299123083 | 2502019325 | HLAC0264 | |  |
| cHOG0320 | VNG2096 | RRNAC2939 | HVO_0455 | hbor_27400 | hmuk_0971 | huta_2121 | NP0570A | HQ1387A | GI-335337428 | GI-289530696 | GI-299123095 | 2502018669 | HLAC0416 | |  |
| cHOG0321 | VNG2423 | RRNAC2717 | HVO_2965 | hbor_02910 | hmuk_0821 | huta_0222 | NP0274A | HQ1044A | GI-335336598 | GI-289531108 | GI-299123239 | 2502015660 | HLAC2730 | |  |
| cHOG0322 | VNG1935 | RRNAC3282 | HVO_2202 | hbor_06400 | hmuk_1414 | huta_1302 | NP2400A | HQ3149A | GI-335337286 | GI-289531839 | GI-299124040 | 2502016249 | HLAC1893 | |  |
| cHOG0323 | VNG2210 | RRNAC2964 | HVO_2952 | hbor_03050 | hmuk_0666 | huta_1669 | NP0444A | HQ1047A | GI-335336537 | GI-289530912 | GI-299123072 | 2502019267 | HLAC2070 | |  |
| cHOG0324 | VNG1797 | RRNAC3348 | HVO_2338 | hbor_07480 | hmuk_1644 | huta_0058 | NP4524A | HQ3322A | GI-335337463 | GI-289530482 | GI-299124293 | 2502018956 | HLAC2302 | |  |
| cHOG0325 | VNG1843 | RRNAC3447 | HVO_2514 | hbor_09010 | hmuk_1374 | huta_3005 | NP2490A | HQ2779A | GI-335337059 | GI-289532178 | GI-299124336 | 2502016197 | HLAC1852 | |  |
| cHOG0326 | VNG2331 | RRNAC3087 | HVO_0077 | hbor_01440 | hmuk_1104 | huta_1761 | NP1328A | HQ3451A | GI-335336906 | GI-289531232 | GI-299123161 | 2502019328 | HLAC2253 | |  |
| cHOG0327 | VNG0251 | RRNAC1672 | HVO_0675 | hbor_25420 | hmuk_3219 | huta_2722 | NP0844A | HQ1271A | GI-335337866 | GI-289530306 | GI-299125641 | 2502018319 | HLAC0495 | |  |
| cHOG0328 | VNG1174 | RRNAC0864 | HVO_1670 | hbor_15430 | hmuk_2237 | huta_0407 | NP2064A | HQ2568A | GI-335336306 | GI-289529927 | GI-299124604 | 2502017712 | HLAC1041 | |  |
| cHOG0329 | VNG1162 | RRNAC0255 | HVO_2743 | hbor_12110 | hmuk_2668 | huta_0079 | NP3672A | HQ2880A | GI-335339432 | GI-289530323 | GI-299124772 | 2502018028 | HLAC2107 | |  |
| cHOG0330 | VNG0572 | RRNAC3539 | HVO_1062 | hbor_22470 | hmuk_2856 | huta_0567 | NP1374A | HQ1683A | GI-335339309 | GI-289531691 | GI-299125431 | 2502016497 | HLAC0837 | |  |
| cHOG0331 | VNG1301 | RRNAC1236 | HVO_1654 | hbor_15620 | hmuk_2310 | huta_0509 | NP3116A | HQ2556A | GI-335338477 | GI-289532196 | GI-299124627 | 2502017294 | HLAC1358 | |  |
| cHOG0333 | VNG1245 | RRNAC0753 | HVO_1323 | hbor_19080 | hmuk_2348 | huta_3025 | NP3982A | HQ2762A | GI-335338362 | GI-289532595 | GI-299124920 | 2502014130 | HLAC1876 | |  |
| cHOG0334 | VNG1027 | RRNAC0537 | HVO_1300 | hbor_19250 | hmuk_2370 | huta_0358 | NP2182A | HQ3360A | GI-335337888 | GI-289529838 | GI-299124527 | 2502017528 | HLAC0683 | |  |
| cHOG0335 | VNG2087 | RRNAC2794 | HVO_0448 | hbor_27470 | hmuk_0716 | huta_1775 | NP0082A | HQ1384A | GI-335337409 | GI-289530680 | GI-299123784 | 2502018799 | HLAC0375 | |  |
| cHOG0336 | VNG1766 | RRNAC1923 | HVO_2298 | hbor_07120 | hmuk_1695 | huta_2576 | NP4756A | HQ2853A | GI-335337530 | GI-289530434 | GI-299124260 | 2502018752 | HLAC2161 | |  |
| cHOG0337 | VNG0632 | RRNAC1431 | HVO_0976 | hbor_21490 | hmuk_1914 | huta_0708 | NP2286A | HQ1635A | GI-335339200 | GI-289532456 | GI-299125385 | 2502016733 | HLAC0700 | |  |
| cHOG0338 | VNG1136 | RRNAC0062 | HVO_2781 | hbor_12540 | hmuk_2592 | huta_2521 | NP2834A | HQ2941A | GI-335336141 | GI-289529660 | GI-299124794 | 2502018189 | HLAC1819 | |  |
| cHOG0339 | VNG1793 | RRNAC1732 | HVO_2336 | hbor_07460 | hmuk_1649 | huta_0056 | NP4528A | HQ3323A | GI-335337459 | GI-289530479 | GI-299124290 | 2502018959 | HLAC2223 | |  |
| cHOG0340 | VNG2227 | RRNAC2479 | HVO_0134 | hbor_00890 | hmuk_0700 | huta_1272 | NP1006A | HQ3051A | GI-335336998 | GI-289531312 | GI-299123795 | 2502019307 | HLAC2661 | |  |
| cHOG0341 | VNG1246 | RRNAC0805 | HVO_1324 | hbor_19060 | hmuk_2347 | huta_3026 | NP4180A | HQ2760A | GI-335338361 | GI-289532594 | GI-299124921 | 2502014129 | HLAC1880 | |  |
| cHOG0342 | VNG0889 | RRNAC0455 | HVO_1573 | hbor_16520 | hmuk_2730 | huta_0133 | NP3500A | HQ2651A | GI-335336318 | GI-289529545 | GI-299125419 | 2502017901 | HLAC0994 | |  |
| cHOG0343 | VNG0921 | RRNAC0908 | HVO_1707 | hbor_15040 | hmuk_2275 | huta_1874 | NP3648A | HQ2543A | GI-335339079 | GI-289532421 | GI-299124974 | 2502016675 | HLAC0472 | |  |
| cHOG0344 | VNG2226 | RRNAC2891 | HVO_0133 | hbor_00920 | hmuk_0699 | huta_1270 | NP1008A | HQ3049A | GI-335336999 | GI-289531313 | GI-299123794 | 2502019311 | HLAC2662 | |  |
| cHOG0345 | VNG0243 | RRNAC1758 | HVO_0658 | hbor_25520 | hmuk_0017 | huta_2665 | NP5228A | HQ1281A | GI-335338228 | GI-289531616 | GI-299125622 | 2502017091 | HLAC0572 | |  |
| cHOG0346 | VNG1842 | RRNAC3452 | HVO_2640 | hbor_09350 | hmuk_1377 | huta_3008 | NP2484A | HQ3027A | GI-335337056 | GI-289531504 | GI-299124333 | 2502016200 | HLAC1602 | |  |
| cHOG0347 | VNG1743 | RRNAC0313 | HVO_2632 | hbor_09430 | hmuk_1356 | huta_2365 | NP4938A | HQ3190A | GI-335338104 | GI-289529863 | GI-299124225 | 2502017731 | HLAC1998 | |  |
| cHOG0348 | VNG1259 | RRNAC1129 | HVO_1123 | hbor_21210 | hmuk_2414 | huta_2076 | NP2680A | HQ1652A | GI-335339549 | GI-289530017 | GI-299124895 | 2502018071 | HLAC0323 | |  |
| cHOG0349 | VNG0711 | RRNAC1254 | HVO_1250 | hbor_19790 | hmuk_2152 | huta_2855 | NP4154A | HQ1809A | GI-335338628 | GI-289532725 | GI-299124543 | 2502017147 | HLAC1351 | |  |
| cHOG0350 | VNG1417 | RRNAC0994 | HVO_2867 | hbor_13780 | hmuk_2212 | huta_2942 | NP2996A | HQ2408A | GI-335338498 | GI-289531511 | GI-299124847 | 2502017316 | HLAC1527 | |  |
| cHOG0351 | VNG2615 | RRNAC2098 | HVO_0421 | hbor_27720 | hmuk_0301 | huta_0503 | NP0198A | HQ1369A | GI-335338818 | GI-289530768 | GI-299125731 | 2502019160 | HLAC0722 | |  |
| cHOG0352 | VNG1510 | RRNAC0974 | HVO_2888 | hbor_14000 | hmuk_2192 | huta_2930 | NP2872A | HQ1798A | GI-335337808 | GI-289530105 | GI-299124860 | 2502018346 | HLAC1480 | |  |
| cHOG0353 | VNG1402 | RRNAC1037 | HVO_2853 | hbor_13640 | hmuk_2225 | huta_2840 | NP2662A | HQ3006A | GI-335336295 | GI-289529918 | GI-299124834 | 2502017933 | HLAC2598 | |  |
| cHOG0354 | VNG2153 | RRNAC3146 | HVO_0303 | hbor_28690 | hmuk_1268 | huta_1426 | NP0604A | HQ3255A | GI-335337206 | GI-289530613 | GI-299123422 | 2502018981 | HLAC0241 | |  |
| cHOG0355 | VNG1649 | RRNAC1520 | HVO_2456 | hbor_10790 | hmuk_1771 | huta_1281 | NP3338A | HQ3169A | GI-335336479 | GI-289529312 | GI-299123824 | 2502017769 | HLAC1939 | |  |
| cHOG0356f | VNG0789 | RRNAC1427 | HVO_1147 | hbor_20940 | hmuk_2133 | huta_0724 | NP3206A | HQ2360A | GI-335338224 | GI-289531613 | GI-299125211 | 2502017016 | HLAC0616 | |  |
| cHOG0357 | VNG2110 | RRNAC2518 | HVO_0339 | hbor_28320 | hmuk_0845 | huta_1790 | NP1318A | HQ3056A | GI-335339521 | GI-289530411 | GI-299123352 | 2502018060 | HLAC0272 | |  |
| cHOG0358 | VNG1385 | RRNAC1106 | HVO_1658 | hbor_15550 | hmuk_2317 | huta_0299 | NP4198A | HQ2561A | GI-335336323 | GI-289529540 | GI-299124614 | 2502017895 | HLAC1294 | |  |
| cHOG0359 | VNG2148 | RRNAC3150 | HVO_0307 | hbor_28650 | hmuk_1272 | huta_1428 | NP5030A | HQ3253A | GI-335337212 | GI-289530608 | GI-299123428 | 2502018987 | HLAC0389 | |  |
| cHOG0360 | VNG1251 | RRNAC1139 | HVO_1333 | hbor_19010 | hmuk_2425 | huta_0549 | NP4002A | HQ2752A | GI-335338351 | GI-289532587 | GI-299124926 | 2502014228 | HLAC0265 | |  |
| cHOG0361 | VNG1038 | RRNAC0300 | HVO_1290 | hbor_19360 | hmuk_2759 | huta_2750 | NP2132A | HQ3368A | GI-335337797 | GI-289530111 | GI-299124534 | 2502018364 | HLAC1346 | |  |
| cHOG0362 | VNG1149 | RRNAC0080 | HVO_2724 | hbor_11920 | hmuk_2607 | huta_2048 | NP3698A | HQ2890A | GI-335336128 | GI-289529679 | GI-299124784 | 2502018176 | HLAC2047 | |  |
| cHOG0363 | VNG0309 | RRNAC1881 | HVO_0790 | hbor_24270 | hmuk_3036 | huta_1178 | NP3160A | HQ1156A | GI-335339379 | GI-289532018 | GI-299125904 | 2502016463 | HLAC1274 | |  |
| cHOG0364 | VNG0650 | RRNAC1272 | HVO_0990 | hbor_21350 | hmuk_2163 | huta_2844 | NP2316A | HQ1649A | GI-335339213 | GI-289532443 | GI-299125372 | 2502016720 | HLAC0324 | |  |
| cHOG0365 | VNG2512 | RRNAC2133 | HVO_1797 | hbor_04230 | hmuk_0378 | huta_1893 | NP0670A | HQ1121A | GI-335338916 | GI-289530541 | GI-299126024 | 2502019048 | HLAC1796 | |  |
| cHOG0366g | VNG2211 | RRNAC2963 | HVO_2953 | hbor_03040 | hmuk_0667 | huta_1668 | NP0446A | HQ1045A | GI-335336536 | GI-289530911 | GI-299123073 | 2502019266 | HLAC2506 | |  |
| cHOG0367 | VNG2263 | RRNAC2854 | HVO_0180 | hbor_00350 | hmuk_1019 | huta_1792 | NP1238A | HQ3685A | GI-335336941 | GI-289531267 | GI-299123456 | 2502015755 | HLAC0434 | |  |
| cHOG0368 | VNG2239 | RRNAC2500 | HVO_0144 | hbor_29660 | hmuk_1067 | huta_2187 | NP2254A | HQ3403A | GI-335336641 | GI-289531092 | GI-299123802 | 2502015643 | HLAC0076 | |  |
| cHOG0369 | VNG1729 | RRNAC3342 | HVO_2493 | hbor_09230 | hmuk_1733 | huta_2322 | NP4916A | HQ2764A | GI-335336327 | GI-289529512 | GI-299124201 | 2502017886 | HLAC1812 | |  |
| cHOG0371 | VNG1294 | RRNAC1230 | HVO_1637 | hbor_15820 | hmuk_2304 | huta_0515 | NP3090A | HQ2586A | GI-335338565 | GI-289532662 | GI-299124633 | 2502017287 | HLAC0806 | |  |
| cHOG0372 | VNG2294 | RRNAC2529 | HVO_2988 | hbor_02580 | hmuk_0859 | huta_2122 | NP1268A | HQ1025A | GI-335336630 | GI-289530956 | GI-299123098 | 2502015695 | HLAC2699 | |  |
| cHOG0373 | VNG2374 | RRNAC2663 | HVO_0008 | hbor_02150 | hmuk_0758 | huta_1243 | NP0550A | HQ1005A | GI-335336821 | GI-289531155 | GI-299123190 | 2502015606 | HLAC2743 | |  |
| cHOG0374 | VNG1646 | RRNAC1517 | HVO_2453 | hbor_10760 | hmuk_1768 | huta_1284 | NP3344A | HQ3166A | GI-335336482 | GI-289529309 | GI-299123821 | 2502017766 | HLAC1942 | |  |
| cHOG0375 | VNG0391 | RRNAC0632 | HVO_1077 | hbor_22660 | hmuk_2825 | huta_2709 | NP0382A | HQ1669A | GI-335339276 | GI-289531818 | GI-299125580 | 2502016969 | HLAC0801 | |  |
| cHOG0376 | VNG2213 | RRNAC2961 | HVO_2955 | hbor_03020 | hmuk_0671 | huta_1659 | NP0564A | HQ1046A | GI-335336522 | GI-289530898 | GI-299123075 | 2502019256 | HLAC2508 | |  |
| cHOG0377 | VNG0296 | RRNAC1813 | HVO_0774 | hbor_24380 | hmuk_3205 | huta_2904 | NP1180A | HQ1167A | GI-335339394 | GI-289532033 | GI-299125892 | 2502016922 | HLAC0548 | |  |
| cHOG0378 | VNG1118 | RRNAC1406 | HVO_0916 | hbor_23080 | hmuk_1953 | huta_0243 | NP1884A | HQ1603A | GI-335337782 | GI-289530130 | GI-299124746 | 2502018393 | HLAC0892 | |  |
| cHOG0379 | VNG1132 | RRNAC0058 | HVO_2784 | hbor_12570 | hmuk_2589 | huta_2524 | NP2828A | HQ2944A | GI-335336144 | GI-289529657 | GI-299124797 | 2502018192 | HLAC1816 | |  |
| cHOG0380 | VNG1982 | RRNAC3217 | HVO_1979 | hbor_05770 | hmuk_1624 | huta_0986 | NP2390A | HQ3095A | GI-335338987 | GI-289531908 | GI-299123979 | 2502016350 | HLAC1937 | |  |
| cHOG0381 | VNG0647 | RRNAC1456 | HVO_0987 | hbor_21380 | hmuk_1926 | huta_0693 | NP2312A | HQ1647A | GI-335339211 | GI-289532445 | GI-299125374 | 2502016722 | HLAC0713 | |  |
| cHOG0382 | VNG2021 | RRNAC3100 | HVO_1871 | hbor_04740 | hmuk_1250 | huta_1222 | NP2262A | HQ1094A | GI-335338849 | GI-289530731 | GI-299123918 | 2502019121 | HLAC2193 | |  |
| cHOG0383 | VNG1260 | RRNAC1128 | HVO_1124 | hbor_21190 | hmuk_2413 | huta_0553 | NP4324A | HQ1653A | GI-335339551 | GI-289530016 | GI-299124896 | 2502018073 | HLAC0959 | |  |
| cHOG0384 | VNG0098 | RRNAC2360 | HVO_0483 | hbor_27040 | hmuk_0657 | huta_2545 | NP0008A | HQ1392A | GI-335337032 | GI-289531357 | GI-299125957 | 2502015877 | HLAC2369 | |  |
| cHOG0385 | VNG1541 | RRNAC0472 | HVO_2465 | hbor_10980 | hmuk_2714 | huta_0438 | NP4356A | HQ2858A | GI-335336277 | GI-289529971 | GI-299124423 | 2502017687 | HLAC2207 | |  |
| cHOG0386 | VNG1256 | RRNAC1131 | HVO_1341 | hbor_18920 | hmuk_2416 | huta_0543 | NP2672A | HQ2510A | GI-335338179 | GI-289532279 | GI-299124930 | 2502014197 | HLAC0451 | |  |
| cHOG0387 | VNG2251 | RRNAC2840 | HVO_0167 | hbor_00130 | hmuk_0756 | huta_1513 | NP0968A | HQ3414A | GI-335336660 | GI-289531071 | GI-299123467 | 2502015737 | HLAC0059 | |  |
| cHOG0388 | VNG2056 | RRNAC2827 | HVO_1901 | hbor_05060 | hmuk_0730 | huta_2209 | NP5084A | HQ1335A | GI-335338865 | GI-289530711 | GI-299123835 | 2502019098 | HLAC2398 | |  |
| cHOG0389 | VNG2493 | RRNAC2920 | HVO_0091 | hbor_01310 | hmuk_1115 | huta_1933 | NP0722A | HQ3441A | GI-335336919 | GI-289531244 | GI-299123298 | 2502015784 | HLAC0013 | |  |
| cHOG0390 | VNG0305 | RRNAC1886 | HVO_0787 | hbor_24300 | hmuk_3039 | huta_1169 | NP3166A | HQ1159A | GI-335339382 | GI-289532021 | GI-299125901 | 2502016466 | HLAC1271 | |  |
| cHOG0391 | VNG0758 | RRNAC0862 | HVO_1175 | hbor_20660 | hmuk_2331 | huta_0286 | NP4104A | HQ2490A | GI-335337928 | GI-289532357 | GI-299125308 | 2502016576 | HLAC1709 | |  |
| cHOG0392 | VNG0983 | RRNAC0744 | HVO_1690 | hbor_15210 | hmuk_2387 | huta_0397 | NP3708A | HQ2581A | GI-335338586 | GI-289532672 | GI-299124946 | 2502017260 | HLAC1773 | |  |
| cHOG0393 | VNG1344 | RRNAC0246 | HVO_2908 | hbor_14350 | hmuk_2771 | huta_2646 | NP3770A | HQ2465A | GI-335338731 | GI-289529251 | GI-299124566 | 2502017464 | HLAC1387 | |  |
| cHOG0394 | VNG2011 | RRNAC3177 | HVO_1861 | hbor_04620 | hmuk_1297 | huta_1979 | NP2280A | HQ1102A | GI-335338839 | GI-289530740 | GI-299123908 | 2502019130 | HLAC2151 | |  |
| cHOG0395 | VNG2015 | RRNAC3174 | HVO_1866 | hbor_04660 | hmuk_1294 | huta_1975 | NP2272A | HQ1098A | GI-335338845 | GI-289530735 | GI-299123912 | 2502019125 | HLAC2387 | |  |
| cHOG0396 | VNG1133 | RRNAC0059 | HVO_2783 | hbor_12560 | hmuk_2590 | huta_2523 | NP2830A | HQ2943A | GI-335336143 | GI-289529658 | GI-299124796 | 2502018191 | HLAC1817 | |  |
| cHOG0397 | VNG0340 | RRNAC1638 | HVO_0690 | hbor_25300 | hmuk_3001 | huta_2737 | NP1214A | HQ1263A | GI-335339254 | GI-289531796 | GI-299125657 | 2502016956 | HLAC0528 | |  |
| cHOG0398 | VNG1215 | RRNAC0113 | HVO_2804 | hbor_12750 | hmuk_2631 | huta_0774 | NP4128A | HQ2961A | GI-335338522 | GI-289532525 | GI-299124814 | 2502017219 | HLAC1559 | |  |
| cHOG0399 | VNG2575 | RRNAC2157 | HVO_1954 | hbor_05480 | hmuk_0599 | huta_1406 | NP0402A | HQ3118A | GI-335338768 | GI-289530821 | GI-299125694 | 2502019231 | HLAC2685 | |  |
| cHOG0400 | VNG1360 | RRNAC0034 | HVO_2874 | hbor_13860 | hmuk_2781 | huta_2511 | NP3788A | HQ2417A | GI-335339089 | GI-289532429 | GI-299124695 | 2502016693 | HLAC1952 | |  |
| cHOG0401 | VNG2104 | RRNAC2512 | HVO_0467 | hbor_26880 | hmuk_0838 | huta_1522 | NP0220A | HQ1350A | GI-335337133 | GI-289531395 | GI-299125816 | 2502015962 | HLAC1691 | |  |
| cHOG0402 | VNG2247 | RRNAC2835 | HVO_0161 | hbor_00100 | hmuk_0752 | huta_1520 | NP1062A | HQ3412A | GI-335336651 | GI-289531079 | GI-299123473 | 2502015725 | HLAC2637 | |  |
| cHOG0403 | VNG0234 | RRNAC1680 | HVO_0651 | hbor_25580 | hmuk_0008 | huta_2676 | NP0612A | HQ1286A | GI-335338263 | GI-289531658 | GI-299125612 | 2502017103 | HLAC0567 | |  |
| cHOG0404 | VNG1128 | RRNAC1267 | HVO_1305 | hbor_19200 | hmuk_2160 | huta_2848 | NP4046A | HQ3356A | GI-335338199 | GI-289531593 | GI-299124668 | 2502017059 | HLAC0891 | |  |
| cHOG0405 | VNG1574 | RRNAC1936 | HVO_A0488 | hbor_26080 | hmuk_0139 | huta_2501 | NP4974A | HQ1405A | GI-335338552 | GI-289532274 | GI-299125603 | 2502014204 | HLAC0237 | |  |
| cHOG0406 | VNG0376 | RRNAC0703 | HVO_0717 | hbor_25000 | hmuk_2935 | huta_0895 | NP0810A | HQ1243A | GI-335339330 | GI-289531984 | GI-299125573 | 2502016989 | HLAC0901 | |  |
| cHOG0407 | VNG2053 | RRNAC2829 | HVO_1899 | hbor_05040 | hmuk_0728 | huta_2207 | NP5080A | HQ1337A | GI-335338863 | GI-289530713 | GI-299123833 | 2502019100 | HLAC2396 | |  |
| cHOG0409 | VNG2322 | RRNAC2610 | HVO_0087 | hbor_01350 | hmuk_0925 | huta_1362 | NP0920A | HQ3443A | GI-335336913 | GI-289531238 | GI-299123148 | 2502015778 | HLAC0015 | |  |
| cHOG0410 | VNG2054 | RRNAC2828 | HVO_1900 | hbor_05050 | hmuk_0729 | huta_2208 | NP5082A | HQ1336A | GI-335338864 | GI-289530712 | GI-299123834 | 2502019099 | HLAC2397 | |  |
| cHOG0411 | VNG1945 | RRNAC3268 | HVO_2188 | hbor_06280 | hmuk_1426 | huta_2232 | NP5010A | HQ3131A | GI-335337097 | GI-289531859 | GI-299124028 | 2502016265 | HLAC1249 | |  |
| cHOG0412 | VNG2666 | RRNAC2430 | HVO_0347 | hbor_28240 | hmuk_0515 | huta_0784 | NP0110A | HQ3396A | GI-335337122 | GI-289531368 | GI-299125941 | 2502015945 | HLAC0107 | |  |
| cHOG0413 | VNG0374 | RRNAC0698 | HVO_0719 | hbor_24980 | hmuk_2938 | huta_0893 | NP0814A | HQ1241A | GI-335339480 | GI-289530372 | GI-299125569 | 2502018280 | HLAC0899 | |  |
| cHOG0414 | VNG1702 | RRNAC1601 | HVO_2553 | hbor_10340 | hmuk_1839 | huta_2303 | NP4876A | HQ2831A | GI-335336418 | GI-289529462 | GI-299124069 | 2502017859 | HLAC2438 | |  |
| cHOG0415 | VNG1709 | RRNAC1596 | HVO_2548 | hbor_10390 | hmuk_1844 | huta_2308 | NP4886A | HQ2826A | GI-335336413 | GI-289529467 | GI-299124074 | 2502017864 | HLAC2433 | |  |
| cHOG0416 | VNG1962 | RRNAC3256 | HVO_1996 | hbor_05970 | hmuk_1444 | huta_1242 | NP3054A | HQ3084A | GI-335338956 | GI-289531879 | GI-299123983 | 2502016331 | HLAC2467 | |  |
| cHOG0417 | VNG1818 | RRNAC3484 | HVO_2506 | hbor_09080 | hmuk_1568 | huta_0419 | NP4826A | HQ2772A | GI-335339010 | GI-289531931 | GI-299124310 | 2502016384 | HLAC1959 | |  |
| cHOG0418 | VNG1388 | RRNAC1105 | HVO_1659 | hbor_15530 | hmuk_2247 | huta_0300 | NP4196A | HQ2562A | GI-335338151 | GI-289529741 | GI-299124613 | 2502018099 | HLAC1441 | |  |
| cHOG0419 | VNG2285 | RRNAC2525 | HVO_2993 | hbor_02510 | hmuk_0855 | huta_1780 | NP1276A | HQ1021A | GI-335336618 | GI-289530975 | GI-299123100 | 2502015688 | HLAC2136 | |  |
| cHOG0420 | VNG0089 | RRNAC2256 | HVO_0474 | hbor_26940 | hmuk_0523 | huta_2560 | NP0020A | HQ1357A | GI-335337044 | GI-289531364 | GI-299125945 | 2502015939 | HLAC0342 | |  |
| cHOG0421 | VNG1343 | RRNAC0238 | HVO_2909 | hbor_14360 | hmuk_2770 | huta_2645 | NP3768A | HQ2464A | GI-335338732 | GI-289529252 | GI-299124567 | 2502017465 | HLAC1388 | |  |
| cHOG0422 | VNG1992 | RRNAC3210 | HVO_1967 | hbor_05630 | hmuk_1318 | huta_2126 | NP4992A | HQ3101A | GI-335338994 | GI-289531916 | GI-299123972 | 2502016361 | HLAC2287 | |  |
| cHOG0423 | VNG1864 | RRNAC3423 | HVO_2601 | hbor_09750 | hmuk_1511 | huta_0074 | NP3194A | HQ3207A | GI-335337235 | GI-289532145 | GI-299124356 | 2502016160 | HLAC2053 | |  |
| cHOG0424 | VNG1117 | RRNAC1405 | HVO_0929 | hbor_23040 | hmuk_1954 | huta_0242 | NP1886A | HQ1624A | GI-335337785 | GI-289530128 | GI-299124745 | 2502018390 | HLAC0733 | |  |
| cHOG0425 | VNG1816 | RRNAC3365 | HVO_2507 | hbor_09070 | hmuk_1554 | huta_0418 | NP4828A | HQ2773A | GI-335339013 | GI-289531934 | GI-299124307 | 2502016388 | HLAC2326 | |  |
| cHOG0426 | VNG0320 | RRNAC1805 | HVO_0800 | hbor_24170 | hmuk_2989 | huta_2910 | NP1128A | HQ1491A | GI-335339375 | GI-289532004 | GI-299125675 | 2502016450 | HLAC0605 | |  |
| cHOG0428 | VNG2465 | RRNAC3116 | HVO_0119 | hbor_01040 | hmuk_1230 | huta_1329 | NP0184A | HQ3420A | GI-335336694 | GI-289531039 | GI-299123338 | 2502015828 | HLAC0822 | |  |
| cHOG0430 | VNG1508 | RRNAC0969 | HVO_2883 | hbor_13950 | hmuk_2189 | huta_2928 | NP2070A | HQ1801A | GI-335337816 | GI-289530099 | GI-299124864 | 2502018496 | HLAC1479 | |  |
| cHOG0431 | VNG0651 | RRNAC1274 | HVO_0991 | hbor_21340 | hmuk_2164 | huta_2843 | NP2318A | HQ1650A | GI-335339215 | GI-289532442 | GI-299125371 | 2502016719 | HLAC1039 | |  |
| cHOG0432 | VNG1576 | RRNAC1938 | HVO_A0553 | hbor_26070 | hmuk_1854 | huta_2504 | NP5126A | HQ1404A | GI-335338557 | GI-289532269 | GI-299125604 | 2502014209 | HLAC0238 | |  |
| cHOG0433 | VNG1042 | RRNAC0347 | HVO_1492 | hbor_17400 | hmuk_2664 | huta_0337 | NP2188A | HQ2410A | GI-335337792 | GI-289530114 | GI-299124539 | 2502018371 | HLAC1172 | |  |
| cHOG0434 | VNG2627 | RRNAC2113 | HVO_0406 | hbor_27850 | hmuk_0315 | huta_0324 | NP0750A | HQ1398A | GI-335337378 | GI-289530636 | GI-299125751 | 2502018918 | HLAC0040 | |  |
| cHOG0435 | VNG2505 | RRNAC2967 | HVO_2947 | hbor_03100 | hmuk_0663 | huta_1676 | NP0694A | HQ1050A | GI-335336547 | GI-289530922 | GI-299125238 | 2502019275 | HLAC2670 | |  |
| cHOG0436 | VNG1798 | RRNAC3352 | HVO_2343 | hbor_07530 | hmuk_1640 | huta_0063 | NP4518A | HQ3320A | GI-335337465 | GI-289530485 | GI-299124294 | 2502018950 | HLAC1949 | |  |
| cHOG0437 | VNG0749 | RRNAC1067 | HVO_2849 | hbor_13600 | hmuk_2231 | huta_0412 | NP3444A | HQ3003A | GI-335338332 | GI-289532566 | GI-299124830 | 2502017370 | HLAC0032 | |  |
| cHOG0438 | VNG1718 | RRNAC1590 | HVO_2542 | hbor_10450 | hmuk_1850 | huta_2314 | NP4898A | HQ2820A | GI-335336407 | GI-289529473 | GI-299124080 | 2502017870 | HLAC2427 | |  |
| cHOG0439 | VNG1866 | RRNAC3421 | HVO_2600 | hbor_09760 | hmuk_1509 | huta_0071 | NP3190A | HQ3208A | GI-335337237 | GI-289532143 | GI-299124358 | 2502016158 | HLAC2052 | |  |
| cHOG0440 | VNG1233 | RRNAC0322 | HVO_1309 | hbor_19160 | hmuk_2642 | huta_0500 | NP3076A | HQ3353A | GI-335338208 | GI-289531601 | GI-299124658 | 2502017044 | HLAC1328 | |  |
| cHOG0441 | VNG1859 | RRNAC3429 | HVO_2610 | hbor_09700 | hmuk_1524 | huta_1388 | NP3200A | HQ3205A | GI-335337072 | GI-289532168 | GI-299124351 | 2502016181 | HLAC2141 | |  |
| cHOG0442 | VNG0349 | RRNAC1645 | HVO_0681 | hbor_25370 | hmuk_3010 | huta_2731 | NP1222A | HQ1267A | GI-335339052 | GI-289531670 | GI-299125649 | 2502017075 | HLAC0259 | |  |
| cHOG0443g | VNG0195 | RRNAC2001 | HVO_0583 | hbor_26230 | hmuk_0167 | huta_2408 | NP5328A | HQ1413A | GI-335337627 | GI-289530215 | GI-299125592 | 2502018467 | HLAC0525 | |  |
| cHOG0444 | VNG1727 | RRNAC3343 | HVO_2494 | hbor_09220 | hmuk_1734 | huta_2321 | NP4914A | HQ2765A | GI-335336328 | GI-289529511 | GI-299124200 | 2502017885 | HLAC1813 | |  |
| cHOG0445 | VNG1665 | RRNAC1532 | HVO_2383 | hbor_08160 | hmuk_1786 | huta_0907 | NP1788A | HQ3173A | GI-335336471 | GI-289529325 | GI-299123947 | 2502017786 | HLAC1629 | |  |
| cHOG0447 | VNG2186 | RRNAC2638 | HVO_0230 | hbor_00840 | hmuk_1050 | huta_2169 | NP5090A | HQ3651A | GI-335336616 | GI-289530972 | GI-299123110 | 2502015686 | HLAC0075 | |  |
| cHOG0448 | VNG0322 | RRNAC1802 | HVO_0802 | hbor_24150 | hmuk_2991 | huta_2912 | NP1188A | HQ1575A | GI-335339377 | GI-289532002 | GI-299125673 | 2502016452 | HLAC0560 | |  |
| cHOG0449 | VNG0406 | RRNAC0175 | HVO_0822 | hbor_23950 | hmuk_3139 | huta_0604 | NP4492A | HQ1560A | GI-335339126 | GI-289531744 | GI-299125785 | 2502018837 | HLAC0655 | |  |
| cHOG0450 | VNG1037 | RRNAC0511 | HVO_1291 | hbor_19350 | hmuk_2383 | huta_0274 | NP2134A | HQ3367A | GI-335339552 | GI-289530015 | GI-299124533 | 2502017920 | HLAC1344 | |  |
| cHOG0451 | VNG0094 | RRNAC2259 | HVO_0476 | hbor_26970 | hmuk_0526 | huta_2558 | NP0016A | HQ1358A | GI-335337041 | GI-289531362 | GI-299125948 | 2502015937 | HLAC2177 | |  |
| cHOG0452 | VNG1815 | RRNAC3363 | HVO_2508 | hbor_09060 | hmuk_1553 | huta_0417 | NP4830A | HQ2774A | GI-335339014 | GI-289531935 | GI-299124306 | 2502016389 | HLAC2325 | |  |
| cHOG0453 | VNG0640 | RRNAC1451 | HVO_0982 | hbor_21430 | hmuk_1921 | huta_0702 | NP2300A | HQ1641A | GI-335339206 | GI-289532450 | GI-299125379 | 2502016727 | HLAC0708 | |  |
| cHOG0454 | VNG2311 | RRNAC2601 | HVO_0252 | hbor_29380 | hmuk_0887 | huta_2108 | NP1418A | HQ3707A | GI-335336593 | GI-289530936 | GI-299123872 | 2502015637 | HLAC0405 | |  |
| cHOG0455 | VNG0893 | RRNAC0448 | HVO_1579 | hbor_16440 | hmuk_2738 | huta_1385 | NP3510A | HQ2647A | GI-335338150 | GI-289529743 | GI-299125256 | 2502018098 | HLAC0938 | |  |
| cHOG0456 | VNG2163 | RRNAC2748 | HVO_0290 | hbor_28830 | hmuk_1029 | huta_1632 | NP5152A | HQ3265A | GI-335337182 | GI-289531444 | GI-299123411 | 2502016055 | HLAC0138 | |  |
| cHOG0457 | VNG1920 | RRNAC3291 | HVO_2213 | hbor_06510 | hmuk_1600 | huta_1278 | NP3172A | HQ3142A | GI-335337293 | GI-289531474 | GI-299124054 | 2502016240 | HLAC1886 | |  |
| cHOG0458 | VNG0489 | RRNAC3334 | HVO_1589 | hbor_16260 | hmuk_2850 | huta_0290 | NP0346A | HQ2641A | GI-335339574 | GI-289529806 | GI-299125547 | 2502017636 | HLAC0681 | |  |
| cHOG0459 | VNG1906 | RRNAC3314 | HVO_2351 | hbor_07640 | hmuk_1580 | huta_2261 | NP1962A | HQ3315A | GI-335337272 | GI-289532058 | GI-299123486 | 2502016095 | HLAC1365 | |  |
| cHOG0460 | VNG1303 | RRNAC1111 | HVO_1656 | hbor_15590 | hmuk_2314 | huta_0507 | NP3966A | HQ2559A | GI-335338480 | GI-289532192 | GI-299124624 | 2502017297 | HLAC1296 | |  |
| cHOG0461 | VNG0597 | RRNAC0849 | HVO_1170 | hbor_20710 | hmuk_2320 | huta_0399 | NP4404A | HQ2497A | GI-335338264 | GI-289532404 | GI-299125295 | 2502016660 | HLAC2361 | |  |
| cHOG0462 | VNG0228 | RRNAC1694 | HVO_0647 | hbor_25620 | hmuk_0029 | huta_2424 | NP0620A | HQ1289A | GI-335338261 | GI-289531653 | GI-299125610 | 2502017106 | HLAC0571 | |  |
| cHOG0463 | VNG1937 | RRNAC3279 | HVO_2201 | hbor_06390 | hmuk_1415 | huta_1304 | NP2398A | HQ3125A | GI-335337284 | GI-289531841 | GI-299124039 | 2502016251 | HLAC1894 | |  |
| cHOG0464 | VNG1938 | RRNAC3278 | HVO_2198 | hbor_06350 | hmuk_1418 | huta_1308 | NP2394A | HQ3127A | GI-335337086 | GI-289531845 | GI-299124037 | 2502016254 | HLAC2018 | |  |
| cHOG0465 | VNG1611 | RRNAC1735 | HVO_2417 | hbor_08500 | hmuk_1737 | huta_2763 | NP4844A | HQ2867A | GI-335336168 | GI-289529639 | GI-299124398 | 2502017671 | HLAC1933 | |  |
| cHOG0466 | VNG2643 | RRNAC2082 | HVO_0400 | hbor_27910 | hmuk_0615 | huta_0145 | NP0430A | HQ3109A | GI-335338851 | GI-289530729 | GI-299125752 | 2502019118 | HLAC0045 | |  |
| cHOG0467 | VNG1389 | RRNAC1103 | HVO_1660 | hbor_15520 | hmuk_2246 | huta_0301 | NP4194A | HQ2563A | GI-335338152 | GI-289529740 | GI-299124612 | 2502018100 | HLAC1442 | |  |
| cHOG0468 | VNG2352 | RRNAC2701 | HVO_0054 | hbor_01650 | hmuk_0796 | huta_1488 | NP0328A | HQ3709A | GI-335336881 | GI-289531209 | GI-299123177 | 2502019362 | HLAC0293 | |  |
| cHOG0469 | VNG0482 | RRNAC0639 | HVO_1584 | hbor_16400 | hmuk_2893 | huta_2471 | NP1474A | HQ2643A | GI-335339589 | GI-289529796 | GI-299125859 | 2502017619 | HLAC0941 | |  |
| cHOG0470 | VNG2044 | RRNAC2492 | HVO_1894 | hbor_04990 | hmuk_1175 | huta_1509 | NP5046A | HQ1342A | GI-335337483 | GI-289530504 | GI-299123943 | 2502018695 | HLAC2391 | |  |
| cHOG0471f | VNG0759 | RRNAC0863 | HVO_1176 | hbor_20650 | hmuk_2332 | huta_0287 | NP4102A | HQ2489A | GI-335337929 | GI-289532356 | GI-299125309 | 2502016575 | HLAC1710 | |  |
| cHOG0472 | VNG1479 | RRNAC0876 | HVO_2705 | hbor_11600 | hmuk_2084 | huta_0189 | NP4336A | HQ2714A | GI-335336193 | GI-289529942 | GI-299124525 | 2502018213 | HLAC2502 | |  |
| cHOG0473 | VNG2523 | RRNAC2223 | HVO_1820 | hbor_03870 | hmuk_0221 | huta_0968 | NP1710A | HQ1063A | GI-335338872 | GI-289530531 | GI-299126015 | 2502019076 | HLAC1910 | |  |
| cHOG0475 | VNG0240 | RRNAC1671 | HVO_0656 | hbor_25540 | hmuk_0016 | huta_2671 | NP5230A | HQ1282A | GI-335339057 | GI-289531664 | GI-299125617 | 2502017096 | HLAC0658 | |  |
| cHOG0476 | VNG0410 | RRNAC0179 | HVO_0824 | hbor_23930 | hmuk_3144 | huta_0603 | NP1476A | HQ1558A | GI-335339111 | GI-289531750 | GI-299125789 | 2502018832 | HLAC0582 | |  |
| cHOG0477 | VNG1305 | RRNAC1109 | HVO_1657 | hbor_15560 | hmuk_2316 | huta_0506 | NP3972A | HQ2560A | GI-335338490 | GI-289532181 | GI-299124616 | 2502017308 | HLAC1295 | |  |
| cHOG0478 | VNG1692 | RRNAC1608 | HVO_2561 | hbor_10260 | hmuk_1831 | huta_2295 | NP4860A | HQ2839A | GI-335336426 | GI-289529454 | GI-299124061 | 2502017851 | HLAC2446 | |  |
| cHOG0479 | VNG1145 | RRNAC0077 | HVO_2761 | hbor_12290 | hmuk_2604 | huta_2045 | NP2850A | HQ2925A | GI-335336131 | GI-289529676 | GI-299124786 | 2502018178 | HLAC1829 | |  |
| cHOG0480 | VNG2131 | RRNAC3164 | HVO_0321 | hbor_28500 | hmuk_1289 | huta_2010 | NP0270A | HQ3241A | GI-335337389 | GI-289530645 | GI-299123443 | 2502018820 | HLAC0170 | |  |
| cHOG0481 | VNG2577 | RRNAC2155 | HVO_1949 | hbor_05440 | hmuk_0601 | huta_1404 | NP0394A | HQ3164A | GI-335338770 | GI-289530819 | GI-299125696 | 2502019229 | HLAC2400 | |  |
| cHOG0482 | VNG2106 | RRNAC2514 | HVO_0469 | hbor_26910 | hmuk_0840 | huta_1255 | NP1316A | HQ1352A | GI-335337396 | GI-289530653 | GI-299123359 | 2502018814 | HLAC0298 | |  |
| cHOG0483 | VNG1264 | RRNAC1165 | HVO_1680 | hbor_15340 | hmuk_2408 | huta_0822 | NP4318A | HQ2576A | GI-335338123 | GI-289529760 | GI-299124902 | 2502018077 | HLAC1516 | |  |
| cHOG0484 | VNG0326 | RRNAC1800 | HVO_0809 | hbor_24090 | hmuk_2993 | huta_2914 | NP1192A | HQ1572A | GI-335339271 | GI-289531809 | GI-299125669 | 2502016418 | HLAC0447 | |  |
| cHOG0485 | VNG1105 | RRNAC1415 | HVO_2757 | hbor_12250 | hmuk_2186 | huta_0254 | NP4452A | HQ2903A | GI-335338620 | GI-289532717 | GI-299124756 | 2502017134 | HLAC2535 | |  |
| cHOG0487 | VNG2208 | RRNAC2965 | HVO_2951 | hbor_03060 | hmuk_0665 | huta_1670 | NP0442A | HQ1048A | GI-335336544 | GI-289530919 | GI-299123071 | 2502019272 | HLAC1863 | |  |
| cHOG0488 | VNG2661 | RRNAC2426 | HVO_0351 | hbor_28200 | hmuk_0511 | huta_0780 | NP0118A | HQ3392A | GI-335337126 | GI-289531372 | GI-299125937 | 2502015949 | HLAC0103 | |  |
| cHOG0489 | VNG1852 | RRNAC3430 | HVO_2611 | hbor_09690 | hmuk_1525 | huta_2997 | NP3188A | HQ3204A | GI-335337074 | GI-289532166 | GI-299124350 | 2502016179 | HLAC1931 | |  |
| cHOG0490 | VNG1682 | RRNAC0320 | HVO_2527 | hbor_10660 | hmuk_1361 | huta_2374 | NP4766A | HQ2862A | GI-335337607 | GI-289530235 | GI-299124229 | 2502018343 | HLAC1898 | |  |
| cHOG0491 | VNG2570 | RRNAC2159 | HVO_1956 | hbor_05510 | hmuk_0596 | huta_1801 | NP0954A | HQ3116A | GI-335338931 | GI-289530565 | GI-299125687 | 2502019030 | HLAC0097 | |  |
| cHOG0492 | VNG1169 | RRNAC0259 | HVO_2748 | hbor_12160 | hmuk_2673 | huta_0623 | NP3682A | HQ2895A | GI-335339439 | GI-289530330 | GI-299124767 | 2502018022 | HLAC2278 | |  |
| cHOG0493 | VNG0542 | RRNAC3504 | HVO_0817 | hbor_24000 | hmuk_2885 | huta_2794 | NP1630A | HQ1565A | GI-335339234 | GI-289531782 | GI-299125464 | 2502016928 | HLAC0732 | |  |
| cHOG0494 | VNG2638 | RRNAC2126 | HVO_0396 | hbor_27950 | hmuk_0269 | huta_0005 | NP0512A | HQ3113A | GI-335338859 | GI-289530721 | GI-299125756 | 2502019106 | HLAC1298 | |  |
| cHOG0495 | VNG1198 | RRNAC0359 | HVO_2650 | hbor_11230 | hmuk_2558 | huta_0773 | NP3024A | HQ2910A | GI-335336508 | GI-289529276 | GI-299124461 | 2502017506 | HLAC1638 | |  |
| cHOG0496 | VNG0648 | RRNAC1458 | HVO_0988 | hbor_21370 | hmuk_1927 | huta_0692 | NP2314A | HQ1648A | GI-335339212 | GI-289532444 | GI-299125373 | 2502016721 | HLAC0714 | |  |
| cHOG0497 | VNG1081 | RRNAC0838 | HVO_1469 | hbor_17640 | hmuk_2535 | huta_0815 | NP2726A | HQ1873A | GI-335338672 | GI-289532768 | GI-299124715 | 2502017403 | HLAC1290 | |  |
| cHOG0498 | VNG0593 | RRNAC1489 | HVO_1412 | hbor_18190 | hmuk_2128 | huta_0691 | NP1580A | HQ1525A | GI-335339223 | GI-289532434 | GI-299125275 | 2502016701 | HLAC1434 | |  |
| cHOG0499 | VNG0307 | RRNAC1885 | HVO_0788 | hbor_24290 | hmuk_3038 | huta_1170 | NP3164A | HQ1158A | GI-335339381 | GI-289532020 | GI-299125902 | 2502016465 | HLAC1272 | |  |
| cHOG0500 | VNG1506 | RRNAC0965 | HVO_2880 | hbor_13920 | hmuk_2121 | huta_2924 | NP4344A | HQ1802A | GI-335337820 | GI-289530096 | GI-299124867 | 2502018504 | HLAC2030 | |  |
| cHOG0501 | VNG0414 | RRNAC0189 | HVO_1085 | hbor_22740 | hmuk_3155 | huta_0599 | NP1662A | HQ1661A | GI-335339020 | GI-289531940 | GI-299125797 | 2502016395 | HLAC0925 | |  |
| cHOG0502 | VNG1667 | RRNAC1527 | HVO_2380 | hbor_08130 | hmuk_1780 | huta_2618 | NP3072A | HQ3177A | GI-335336463 | GI-289529417 | GI-299123951 | 2502017806 | HLAC1716 | |  |
| cHOG0503 | VNG0633 | RRNAC1446 | HVO_0977 | hbor_21480 | hmuk_1916 | huta_0707 | NP2290A | HQ1636A | GI-335339201 | GI-289532455 | GI-299125384 | 2502016732 | HLAC0703 | |  |
| cHOG0504 | VNG0284 | RRNAC1872 | HVO_0768 | hbor_24450 | hmuk_3028 | huta_1190 | NP5312A | HQ1173A | GI-335339402 | GI-289531973 | GI-299125876 | 2502016913 | HLAC0639 | |  |
| cHOG0505 | VNG0183 | RRNAC2015 | HVO_0573 | hbor_26330 | hmuk_0185 | huta_2402 | NP5118A | HQ1417A | GI-335337640 | GI-289530202 | GI-299125585 | 2502018632 | HLAC0531 | |  |
| cHOG0506 | VNG0351 | RRNAC3318 | HVO_0734 | hbor_24810 | hmuk_3192 | huta_1972 | NP5182A | HQ1226A | GI-335339450 | GI-289530341 | GI-299125853 | 2502018245 | HLAC0310 | |  |
| cHOG0507 | VNG2665 | RRNAC2429 | HVO_0348 | hbor_28230 | hmuk_0514 | huta_0783 | NP0112A | HQ3395A | GI-335337123 | GI-289531369 | GI-299125940 | 2502015946 | HLAC0106 | |  |
| cHOG0508 | VNG0086 | RRNAC1299 | HVO_2305 | hbor_07200 | hmuk_2502 | huta_0036 | NP1442A | HQ3344A | GI-335337516 | GI-289530445 | GI-299124270 | 2502018883 | HLAC2123 | |  |
| cHOG0509 | VNG2616 | RRNAC2092 | HVO_0417 | hbor_27750 | hmuk_0281 | huta_0152 | NP0944A | HQ1367A | GI-335338823 | GI-289530763 | GI-299125736 | 2502019181 | HLAC1280 | |  |
| cHOG0510 | VNG1029 | RRNAC0535 | HVO_1299 | hbor_19270 | hmuk_2372 | huta_0360 | NP2146A | HQ3361A | GI-335337887 | GI-289529839 | GI-299124528 | 2502017527 | HLAC0686 | |  |
| cHOG0511 | VNG1266 | RRNAC1166 | HVO_1681 | hbor_15330 | hmuk_2407 | huta_0823 | NP3754A | HQ2577A | GI-335338124 | GI-289529759 | GI-299124903 | 2502018078 | HLAC1517 | |  |
| cHOG0512 | VNG1713 | RRNAC1594 | HVO_2546 | hbor_10410 | hmuk_1846 | huta_2310 | NP4890A | HQ2824A | GI-335336411 | GI-289529469 | GI-299124076 | 2502017866 | HLAC2431 | |  |
| cHOG0513 | VNG2060 | RRNAC2820 | HVO_1905 | hbor_05110 | hmuk_0734 | huta_2213 | NP2250A | HQ3105A | GI-335338868 | GI-289530527 | GI-299123852 | 2502019080 | HLAC2544 | |  |
| cHOG0514 | VNG1153 | RRNAC0085 | HVO_2726 | hbor_11940 | hmuk_2609 | huta_2071 | NP3694A | HQ2888A | GI-335336125 | GI-289529682 | GI-299124782 | 2502018174 | HLAC2322 | |  |
| cHOG0515 | VNG6309 | RRNAC1225 | HVO_1454 | hbor_17830 | hmuk_2301 | huta_0520 | NP3518A | HQ1879A | GI-335338567 | GI-289532664 | GI-299124635 | 2502017284 | HLAC1285 | |  |
| cHOG0516 | VNG1409 | RRNAC1003 | HVO_2859 | hbor_13710 | hmuk_2221 | huta_2950 | NP4190A | HQ2794A | GI-335336299 | GI-289529922 | GI-299124836 | 2502017701 | HLAC1178 | |  |
| cHOG0519 | VNG1030 | RRNAC0532 | HVO_1297 | hbor_19280 | hmuk_2373 | huta_0365 | NP2144A | HQ3362A | GI-335337886 | GI-289529840 | GI-299124530 | 2502017526 | HLAC1341 | |  |
| cHOG0520 | VNG2474 | RRNAC2903 | HVO_0100 | hbor_01210 | hmuk_1198 | huta_1010 | NP1358A | HQ3435A | GI-335336928 | GI-289531252 | GI-299123320 | 2502015793 | HLAC2215 | |  |
| cHOG0521 | VNG1497 | RRNAC0801 | HVO_2770 | hbor_12440 | hmuk_2102 | huta_0679 | NP4302A | HQ2933A | GI-335337848 | GI-289530298 | GI-299124876 | 2502018565 | HLAC2234 | |  |
| cHOG0522 | VNG0134 | RRNAC2301 | HVO_0520 | hbor_26820 | hmuk_0564 | huta_2248 | NP0316A | HQ1436A | GI-335337173 | GI-289531421 | GI-299125986 | 2502016012 | HLAC0110 | |  |
| cHOG0523 | VNG0250 | RRNAC1793 | HVO_0674 | hbor_25430 | hmuk_3221 | huta_2721 | NP0848A | HQ1272A | GI-335337868 | GI-289530308 | GI-299125640 | 2502018315 | HLAC1180 | |  |
| cHOG0524 | VNG2252 | RRNAC2847 | HVO_0170 | hbor_00150 | hmuk_0891 | huta_1653 | NP0680A | HQ3690A | GI-335336666 | GI-289531067 | GI-299123465 | 2502015742 | HLAC2659 | |  |
| cHOG0525 | VNG1676 | RRNAC1619 | HVO_2575 | hbor_10090 | hmuk_1813 | huta_1155 | NP1740A | HQ2800A | GI-335336437 | GI-289529441 | GI-299123958 | 2502017834 | HLAC0070 | |  |
| cHOG0526 | VNG1779 | RRNAC1716 | HVO_2315 | hbor_07310 | hmuk_1548 | huta_2829 | NP4550A | HQ3332A | GI-335337507 | GI-289530455 | GI-299124279 | 2502018896 | HLAC2063 | |  |
| cHOG0527 | VNG2140 | RRNAC3158 | HVO_0315 | hbor_28570 | hmuk_1280 | huta_1436 | NP1028A | HQ3245A | GI-335337220 | GI-289530600 | GI-299123436 | 2502018998 | HLAC0280 | |  |
| cHOG0528 | VNG1114 | RRNAC1409 | HVO_0914 | hbor_23110 | hmuk_1948 | huta_0247 | NP4460A | HQ1605A | GI-335339527 | GI-289530416 | GI-299124749 | 2502018066 | HLAC1002 | |  |
| cHOG0529 | VNG0604 | RRNAC1344 | HVO_1440 | hbor_18010 | hmuk_2003 | huta_0094 | NP4474A | HQ1882A | GI-335338277 | GI-289532395 | GI-299125288 | 2502016624 | HLAC1293 | |  |
| cHOG0530 | VNG2430 | RRNAC2667 | HVO_2969 | hbor_02880 | hmuk_0782 | huta_1493 | NP5280A | HQ1042A | GI-335336778 | GI-289530978 | GI-299123244 | 2502015911 | HLAC2719 | |  |
| cHOG0531 | VNG2100 | RRNAC2508 | HVO_0464 | hbor_27320 | hmuk_0834 | huta_1526 | NP1076A | HQ1348A | GI-335337364 | GI-289530700 | GI-299123363 | 2502018679 | HLAC0349 | |  |
| cHOG0532 | VNG2330 | RRNAC3086 | HVO_0078 | hbor_01430 | hmuk_1105 | huta_0927 | NP1326A | HQ3450A | GI-335336907 | GI-289531233 | GI-299123160 | 2502019327 | HLAC2252 | |  |
| cHOG0533 | VNG2574 | RRNAC2158 | HVO_1955 | hbor_05490 | hmuk_0597 | huta_1803 | NP0404A | HQ3117A | GI-335338930 | GI-289530564 | GI-299125688 | 2502019029 | HLAC0098 | |  |
| cHOG0534 | VNG1357 | RRNAC1307 | HVO_2899 | hbor_14200 | hmuk_2050 | huta_0204 | NP3780A | HQ2473A | GI-335338710 | GI-289532797 | GI-299124699 | 2502017442 | HLAC2203 | |  |
| cHOG0535 | VNG1410 | RRNAC1002 | HVO_2860 | hbor_13720 | hmuk_2219 | huta_2949 | NP2602A | HQ2793A | GI-335339649 | GI-289529890 | GI-299124837 | 2502017563 | HLAC2036 | |  |
| cHOG0536 | VNG2017 | RRNAC3173 | HVO_1867 | hbor_04670 | hmuk_1293 | huta_1974 | NP2270A | HQ1097A | GI-335338846 | GI-289530734 | GI-299123913 | 2502019124 | HLAC2388 | |  |
| cHOG0537 | VNG1932 | RRNAC3285 | HVO_2205 | hbor_06430 | hmuk_1410 | huta_1299 | NP2406A | HQ3146A | GI-335337288 | GI-289531837 | GI-299124042 | 2502016247 | HLAC1891 | |  |
| cHOG0538 | VNG0426 | RRNAC0218 | HVO_1385 | hbor_18510 | hmuk_3187 | huta_0231 | NP0364A | HQ2396A | GI-335339037 | GI-289531957 | GI-299125817 | 2502016959 | HLAC0970 | |  |
| cHOG0539 | VNG0787 | RRNAC1429 | HVO_1145 | hbor_20960 | hmuk_2131 | huta_0722 | NP3210A | HQ2358A | GI-335338226 | GI-289531615 | GI-299125209 | 2502017014 | HLAC0618 | |  |
| cHOG0540 | VNG1583 | RRNAC1935 | HVO_0592 | hbor_26120 | hmuk_0140 | huta_2500 | NP5300A | HQ1406A | GI-335337617 | GI-289530225 | GI-299125602 | 2502018481 | HLAC0236 | |  |
| cHOG0541 | VNG0592 | RRNAC0739 | HVO_0848 | hbor_23710 | hmuk_3103 | huta_0584 | NP4496A | HQ1545A | GI-335339222 | GI-289532436 | GI-299125273 | 2502016703 | HLAC0954 | |  |
| cHOG0542 | VNG0865 | RRNAC1361 | HVO_1049 | hbor_22310 | hmuk_1986 | huta_0733 | NP2980A | HQ1692A | GI-335338641 | GI-289532737 | GI-299124698 | 2502017373 | HLAC0612 | |  |
| cHOG0543 | VNG0177 | RRNAC2065 | HVO_0561 | hbor_26510 | hmuk_0277 | huta_2385 | NP0246A | HQ1424A | GI-335337650 | GI-289530193 | GI-299125763 | 2502018436 | HLAC0413 | |  |
| cHOG0545 | VNG1071 | RRNAC0842 | HVO_1090 | hbor_22780 | hmuk_2319 | huta_0387 | NP2762A | HQ1517A | GI-335338661 | GI-289532751 | GI-299124707 | 2502017391 | HLAC0795 | |  |
| cHOG0546 | VNG2126 | RRNAC3074 | HVO_0326 | hbor_28460 | hmuk_1112 | huta_2018 | NP0072A | HQ3236A | GI-335337670 | GI-289530174 | GI-299123448 | 2502018626 | HLAC0355 | |  |
| cHOG0547 | VNG0862 | RRNAC1394 | HVO_1044 | hbor_22260 | hmuk_1964 | huta_0630 | NP4158A | HQ1695A | GI-335338680 | GI-289532774 | GI-299125023 | 2502017411 | HLAC0547 | |  |
| cHOG0548 | VNG2507 | RRNAC2969 | HVO_2943 | hbor_03140 | hmuk_0662 | huta_1678 | NP0428A | HQ1052A | GI-335338407 | GI-289532657 | GI-299123070 | 2502014163 | HLAC0019 | |  |
| cHOG0549 | VNG1431 | RRNAC0052 | HVO_2478 | hbor_11110 | hmuk_2582 | huta_0763 | NP2026A | HQ3013A | GI-335338087 | GI-289529849 | GI-299124452 | 2502017517 | HLAC2335 | |  |
| cHOG0550 | VNG0755 | RRNAC0857 | HVO_1173 | hbor_20680 | hmuk_2326 | huta_0284 | NP4110A | HQ2492A | GI-335337922 | GI-289532361 | GI-299125305 | 2502016581 | HLAC1701 | |  |
| cHOG0551 | VNG1999 | RRNAC3199 | HVO_1958 | hbor_05540 | hmuk_1309 | huta_2601 | NP4484A | HQ3114A | GI-335337228 | GI-289530591 | GI-299123965 | 2502019007 | HLAC2010 | |  |
| cHOG0552 | VNG1547 | RRNAC1490 | HVO_2395 | hbor_08280 | hmuk_1904 | huta_3022 | NP3008A | HQ2299A | GI-335336179 | GI-289529629 | GI-299124414 | 2502017948 | HLAC2349 | |  |
| cHOG0553 | VNG0488 | RRNAC3332 | HVO_1587 | hbor_16330 | hmuk_2851 | huta_2951 | NP0602A | HQ2642A | GI-335339577 | GI-289529805 | GI-299125548 | 2502017633 | HLAC0679 | |  |
| cHOG0554 | VNG1929 | RRNAC3286 | HVO_2207 | hbor_06440 | hmuk_1411 | huta_1298 | NP2478A | HQ3145A | GI-335337289 | GI-289531835 | GI-299124043 | 2502016246 | HLAC1888 | |  |
| cHOG0555 | VNG2333 | RRNAC3094 | HVO_0073 | hbor_01460 | hmuk_1059 | huta_0931 | NP0202A | HQ3702A | GI-335336904 | GI-289531228 | GI-299123165 | 2502019331 | HLAC2552 | |  |
| cHOG0556 | VNG1347 | RRNAC0018 | HVO_2906 | hbor_14320 | hmuk_2774 | huta_2656 | NP3772A | HQ2467A | GI-335338729 | GI-289532808 | GI-299124564 | 2502017462 | HLAC1382 | |  |
| cHOG0557 | VNG0870 | RRNAC1356 | HVO_1053 | hbor_22350 | hmuk_1992 | huta_0084 | NP1454A | HQ1688A | GI-335338658 | GI-289532748 | GI-299124704 | 2502017388 | HLAC0834 | |  |
| cHOG0558 | VNG0266 | RRNAC1854 | HVO_0754 | hbor_24590 | hmuk_3051 | huta_1199 | NP5216A | HQ1184A | GI-335339414 | GI-289531823 | GI-299125873 | 2502016963 | HLAC0189 | |  |
| cHOG0559g | VNG0272 | RRNAC1861 | HVO_0761 | hbor_24520 | hmuk_3017 | huta_1194 | NP1300A | HQ1179A | GI-335339408 | GI-289531829 | GI-299125882 | 2502016904 | HLAC1457 | |  |
| cHOG0560 | VNG1705 | RRNAC1598 | HVO_2551 | hbor_10360 | hmuk_1841 | huta_2305 | NP4880A | HQ2829A | GI-335336416 | GI-289529464 | GI-299124071 | 2502017861 | HLAC2436 | |  |
| cHOG0561 | VNG1771 | RRNAC1703 | HVO_2303 | hbor_07180 | hmuk_1607 | huta_2583 | NP1744A | HQ3346A | GI-335337522 | GI-289530443 | GI-299124266 | 2502018746 | HLAC2382 | |  |
| cHOG0562 | VNG1914 | RRNAC3303 | HVO_2222 | hbor_06580 | hmuk_1591 | huta_1852 | NP3056A | HQ3123A | GI-335337263 | GI-289532114 | GI-299123775 | 2502016126 | HLAC1668 | |  |
| cHOG0563 | VNG2102 | RRNAC2511 | HVO_0466 | hbor_27300 | hmuk_0837 | huta_1523 | NP1314A | HQ1349A | GI-335337360 | GI-289530702 | GI-299123361 | 2502018681 | HLAC0723 | |  |
| cHOG0564 | VNG1693 | RRNAC1607 | HVO_2560 | hbor_10270 | hmuk_1832 | huta_2296 | NP4862A | HQ2838A | GI-335336425 | GI-289529455 | GI-299124062 | 2502017852 | HLAC2445 | |  |
| cHOG0565 | VNG1946 | RRNAC3272 | HVO_2191 | hbor_06300 | hmuk_1421 | huta_2219 | NP5006A | HQ3129A | GI-335337093 | GI-289531852 | GI-299124030 | 2502016263 | HLAC1251 | |  |
| cHOG0566 | VNG0884 | RRNAC0459 | HVO_1570 | hbor_16550 | hmuk_2727 | huta_0129 | NP3480A | HQ2654A | GI-335338116 | GI-289529874 | GI-299125415 | 2502017916 | HLAC1315 | |  |
| cHOG0567 | VNG1397 | RRNAC0866 | HVO_1668 | hbor_15450 | hmuk_2239 | huta_0405 | NP3372A | HQ2566A | GI-335336310 | GI-289529937 | GI-299124606 | 2502017716 | HLAC0952 | |  |
| cHOG0568 | VNG1125 | RRNAC1268 | HVO_1304 | hbor_19210 | hmuk_2161 | huta_2847 | NP4044A | HQ3355A | GI-335338198 | GI-289531592 | GI-299124669 | 2502017060 | HLAC0890 | |  |
| cHOG0570 | VNG0745 | RRNAC1070 | HVO_2846 | hbor_13570 | hmuk_2234 | huta_0409 | NP3450A | HQ3000A | GI-335338335 | GI-289532569 | GI-299124827 | 2502017367 | HLAC0029 | |  |
| cHOG0571 | VNG0389 | RRNAC0715 | HVO_1075 | hbor_22640 | hmuk_3085 | huta_2661 | NP0386A | HQ1671A | GI-335339344 | GI-289532000 | GI-299125578 | 2502016974 | HLAC0957 | |  |
| cHOG0572 | VNG1137 | RRNAC0064 | HVO_2779 | hbor_12520 | hmuk_2593 | huta_2520 | NP2836A | HQ2940A | GI-335336140 | GI-289529661 | GI-299124793 | 2502018187 | HLAC1820 | |  |
| cHOG0573 | VNG0370 | RRNAC0696 | HVO_0721 | hbor_24960 | hmuk_2957 | huta_2469 | NP0818A | HQ1240A | GI-335339478 | GI-289530370 | GI-299125567 | 2502018276 | HLAC0932 | |  |
| cHOG0574 | VNG1615 | RRNAC1740 | HVO_2419 | hbor_08530 | hmuk_1739 | huta_2766 | NP4836A | HQ2868A | GI-335336170 | GI-289529638 | GI-299124397 | 2502017942 | HLAC2313 | |  |
| cHOG0575g | VNG0498 | RRNAC0627 | HVO_1595 | hbor_16200 | hmuk_2831 | huta_0340 | NP4070A | HQ2636A | GI-335339562 | GI-289529899 | GI-299125533 | 2502017657 | HLAC1484 | |  |
| cHOG0576 | VNG0524 | RRNAC1830 | HVO_0859 | hbor_23600 | hmuk_2918 | huta_0376 | NP1504A | HQ1706A | GI-335338251 | GI-289531642 | GI-299125476 | 2502017117 | HLAC0176 | |  |
| cHOG0577 | VNG0438 | RRNAC0656 | HVO_1482 | hbor_17470 | hmuk_3126 | huta_0882 | NP4568A | HQ2414A | GI-335339327 | GI-289531983 | GI-299125831 | 2502016992 | HLAC1430 | |  |
| cHOG0578 | VNG2370 | RRNAC2875 | HVO_0015 | hbor_02240 | hmuk_0688 | huta_2128 | NP0494A | HQ1016A | GI-335336868 | GI-289531190 | GI-299123205 | 2502019383 | HLAC0016 | |  |
| cHOG0579 | VNG1689 | RRNAC1611 | HVO_2564 | hbor_10230 | hmuk_1828 | huta_2292 | NP4854A | HQ2842A | GI-335336429 | GI-289529451 | GI-299124058 | 2502017848 | HLAC2449 | |  |
| cHOG0580 | VNG0255 | RRNAC3320 | HVO_0732 | hbor_24830 | hmuk_3066 | huta_1207 | NP5188A | HQ1228A | GI-335336187 | GI-289529549 | GI-299125867 | 2502018226 | HLAC0307 | |  |
| cHOG0581 | VNG1581 | RRNAC1932 | HVO_0589 | hbor_26150 | hmuk_0142 | huta_2497 | NP5304A | HQ1409A | GI-335337620 | GI-289530222 | GI-299125600 | 2502018478 | HLAC0234 | |  |
| cHOG0582 | VNG1887 | RRNAC3403 | HVO_2516 | hbor_08990 | hmuk_1492 | huta_1587 | NP1964A | HQ2781A | GI-335337300 | GI-289531480 | GI-299124374 | 2502016231 | HLAC1851 | |  |
| cHOG0583 | VNG1994 | RRNAC3206 | HVO_1965 | hbor_05610 | hmuk_1315 | huta_2682 | NP4986A | HQ3076A | GI-335338996 | GI-289531918 | GI-299123970 | 2502016363 | HLAC2062 | |  |
| cHOG0584 | VNG1875 | RRNAC3412 | HVO_2583 | hbor_10010 | hmuk_1499 | huta_0276 | NP2422A | HQ3215A | GI-335337245 | GI-289532131 | GI-299124372 | 2502016146 | HLAC2019 | |  |
| cHOG0585 | VNG0363 | RRNAC0676 | HVO_0726 | hbor_24890 | hmuk_2966 | huta_2452 | NP1158A | HQ1234A | GI-335339472 | GI-289530360 | GI-299125555 | 2502018270 | HLAC0598 | |  |
| cHOG0586 | VNG1608 | RRNAC1502 | HVO_2415 | hbor_08480 | hmuk_1879 | huta_2748 | NP4776A | HQ2865A | GI-335336198 | GI-289529947 | GI-299124400 | 2502017675 | HLAC1242 | |  |
| cHOG0587f | VNG1300 | RRNAC1235 | HVO_1653 | hbor_15650 | hmuk_2308 | huta_0511 | NP3102A | HQ2583A | GI-335338475 | GI-289532198 | GI-299124628 | 2502017292 | HLAC1395 | |  |
| cHOG0588 | VNG1686 | RRNAC1616 | HVO_2573 | hbor_10140 | hmuk_1816 | huta_1145 | NP2634A | HQ2798A | GI-335336435 | GI-289529446 | GI-299123956 | 2502017838 | HLAC1693 | |  |
| cHOG0589 | VNG1276 | RRNAC1173 | HVO_1344 | hbor_18900 | hmuk_2402 | huta_2806 | NP3740A | HQ2517A | GI-335338509 | GI-289532513 | GI-299124912 | 2502017328 | HLAC0849 | |  |
| cHOG0590 | VNG0419 | RRNAC0208 | HVO_1102 | hbor_22910 | hmuk_3179 | huta_0224 | NP4076A | HQ1506A | GI-335339031 | GI-289531951 | GI-299125807 | 2502016406 | HLAC0673 | |  |
| cHOG0591 | VNG2598 | RRNAC2369 | HVO_0436 | hbor_27580 | hmuk_0551 | huta_1409 | NP0464A | HQ1301A | GI-335338799 | GI-289530790 | GI-299125716 | 2502019189 | HLAC0370 | |  |
| cHOG0592 | VNG0718 | RRNAC1620 | HVO_1536 | hbor_16930 | hmuk_1809 | huta_1156 | NP1738A | HQ2676A | GI-335337681 | GI-289530171 | GI-299125185 | 2502018611 | HLAC1119 | |  |
| cHOG0593 | VNG1724 | RRNAC3346 | HVO_2496 | hbor_09180 | hmuk_1736 | huta_2319 | NP4910A | HQ2767A | GI-335336332 | GI-289529508 | GI-299124196 | 2502017882 | HLAC1912 | |  |
| cHOG0594 | VNG1775 | RRNAC1709 | HVO_2312 | hbor_07270 | hmuk_1613 | huta_1755 | NP4500A | HQ3335A | GI-335337510 | GI-289530451 | GI-299124275 | 2502018893 | HLAC2131 | |  |
| cHOG0595 | VNG1493 | RRNAC0797 | HVO_2721 | hbor_11880 | hmuk_2095 | huta_0672 | NP4312A | HQ2893A | GI-335339501 | GI-289530393 | GI-299124881 | 2502018298 | HLAC1477 | |  |
| cHOG0596 | VNG0233 | RRNAC1681 | HVO_0650 | hbor_25590 | hmuk_0007 | huta_2677 | NP0614A | HQ1287A | GI-335338262 | GI-289531657 | GI-299125611 | 2502017104 | HLAC0568 | |  |
| cHOG0597 | VNG0345 | RRNAC1642 | HVO_0684 | hbor_25340 | hmuk_3005 | huta_2734 | NP1220A | HQ1266A | GI-335339244 | GI-289531787 | GI-299125654 | 2502016939 | HLAC0431 | |  |
| cHOG0598 | VNG2654 | RRNAC2413 | HVO_0356 | hbor_28140 | hmuk_0504 | huta_2874 | NP0130A | HQ3388A | GI-335337130 | GI-289531384 | GI-299125929 | 2502015960 | HLAC0152 | |  |
| cHOG0599 | VNG0642 | RRNAC1453 | HVO_0984 | hbor_21410 | hmuk_1923 | huta_0696 | NP2306A | HQ1644A | GI-335339208 | GI-289532448 | GI-299125377 | 2502016725 | HLAC0710 | |  |
| cHOG0600 | VNG2135 | RRNAC3162 | HVO_0319 | hbor_28540 | hmuk_1286 | huta_1441 | NP0264A | HQ3242A | GI-335337225 | GI-289530594 | GI-299123441 | 2502019004 | HLAC0283 | |  |
| cHOG0601 | VNG0259 | RRNAC3323 | HVO_0729 | hbor_24860 | hmuk_3063 | huta_1204 | NP5192A | HQ1231A | GI-335336262 | GI-289529552 | GI-299125870 | 2502018229 | HLAC0304 | |  |
| cHOG0602 | VNG2444 | RRNAC2722 | HVO_0245 | hbor_29440 | hmuk_0825 | huta_1836 | NP0462A | HQ3640A | GI-335336758 | GI-289531016 | GI-299123259 | 2502015930 | HLAC0329 | |  |
| cHOG0603 | VNG2339 | RRNAC2689 | HVO_0064 | hbor_01610 | hmuk_0764 | huta_1618 | NP0478A | HQ3460A | GI-335336892 | GI-289531214 | GI-299123174 | 2502019343 | HLAC0149 | |  |
| cHOG0604 | VNG1901 | RRNAC3309 | HVO_2348 | hbor_07600 | hmuk_1583 | huta_2260 | NP2514A | HQ3317A | GI-335337275 | GI-289532055 | GI-299123803 | 2502016091 | HLAC2276 | |  |
| cHOG0605 | VNG0778 | RRNAC1439 | HVO_1016 | hbor_21950 | hmuk_1908 | huta_0716 | NP3222A | HQ1580A | GI-335338094 | GI-289529855 | GI-299125203 | 2502017743 | HLAC2405 | |  |
| cHOG0606 | VNG1980 | RRNAC3253 | HVO_1989 | hbor_05920 | hmuk_1447 | huta_1107 | NP2324A | HQ3089A | GI-335338981 | GI-289531902 | GI-299123980 | 2502016345 | HLAC1588 | |  |
| cHOG0607 | VNG1366 | RRNAC0040 | HVO_1108 | hbor_22960 | hmuk_2789 | huta_2506 | NP3810A | HQ1504A | GI-335338717 | GI-289532802 | GI-299124689 | 2502017448 | HLAC0716 | |  |
| cHOG0608 | VNG2115 | RRNAC2520 | HVO_0337 | hbor_28340 | hmuk_0847 | huta_1787 | NP1250A | HQ3059A | GI-335339518 | GI-289530408 | GI-299123350 | 2502018057 | HLAC0256 | |  |
| cHOG0609 | VNG0124 | PNG7317 | HVO_0507 | hbor_27250 | hmuk_0580 | huta_1831 | NP0414A | HQ1759A | GI-335337191 | GI-289531458 | GI-299125973 | 2502016037 | HLAC0080 | |  |
| cHOG0610 | VNG1275 | RRNAC1169 | HVO_1346 | hbor_18880 | hmuk_2404 | huta_0828 | NP3742A | HQ2339A | GI-335338511 | GI-289532517 | GI-299124910 | 2502017330 | HLAC1303 | |  |
| cHOG0611 | VNG2587 | RRNAC2378 | HVO_1931 | hbor_05280 | hmuk_0625 | huta_1102 | NP0576A | HQ3150A | GI-335338791 | GI-289530799 | GI-299125707 | 2502019206 | HLAC1378 | |  |
| cHOG0612 | VNG1673 | RRNAC1515 | HVO_2577 | hbor_10060 | hmuk_1801 | huta_0923 | NP1734A | HQ3219A | GI-335336439 | GI-289529438 | GI-299123901 | 2502017831 | HLAC1586 | |  |
| cHOG0613 | VNG2173 | RRNAC2756 | HVO_0283 | hbor_28920 | hmuk_1035 | huta_1952 | NP5144A | HQ3065A | GI-335337011 | GI-289531407 | GI-299123404 | 2502015899 | HLAC0056 | |  |
| cHOG0614 | VNG1359 | RRNAC0032 | HVO_2873 | hbor_13850 | hmuk_2779 | huta_2512 | NP3784A | HQ2418A | GI-335339092 | GI-289532430 | GI-299124700 | 2502016695 | HLAC1613 | |  |
| cHOG0615 | VNG2371 | RRNAC2659 | HVO_0011 | hbor_02200 | hmuk_0907 | huta_1038 | NP0732A | HQ1006A | GI-335336854 | GI-289531179 | GI-299123192 | 2502019398 | HLAC2704 | |  |
| cHOG0616 | VNG2138 | RRNAC3160 | HVO_0317 | hbor_28550 | hmuk_1282 | huta_1438 | NP1032A | HQ3243A | GI-335337222 | GI-289530598 | GI-299123438 | 2502019000 | HLAC0282 | |  |
| cHOG0618 | VNG1173 | RRNAC0261 | HVO_2752 | hbor_12200 | hmuk_2675 | huta_0625 | NP3686A | HQ2898A | GI-335339448 | GI-289530338 | GI-299124763 | 2502018243 | HLAC2341 | |  |
| cHOG0619 | VNG2118 | RRNAC2521 | HVO_0333 | hbor_28380 | hmuk_0849 | huta_1784 | NP1256A | HQ3229A | GI-335337660 | GI-289530186 | GI-299123347 | 2502018424 | HLAC0584 | |  |
| cHOG0620 | VNG2306 | RRNAC2582 | HVO_2972 | hbor_02800 | hmuk_0882 | huta_1550 | NP1406A | HQ1040A | GI-335336584 | GI-289530944 | GI-299123134 | 2502015711 | HLAC2707 | |  |
| cHOG0621 | VNG0306 | RRNAC1916 | HVO_0786 | hbor_24310 | hmuk_3040 | huta_2893 | NP0918A | HQ1160A | GI-335339383 | GI-289532022 | GI-299125900 | 2502016467 | HLAC1270 | |  |
| cHOG0622 | VNG0847 | RRNAC0397 | HVO_1623 | hbor_15940 | hmuk_2498 | huta_1348 | NP3148A | HQ2592A | GI-335338696 | GI-289532790 | GI-299125008 | 2502017427 | HLAC1218 | |  |
| cHOG0623 | VNG2233 | RRNAC2476 | HVO_0135 | hbor_00880 | hmuk_0701 | huta_1605 | NP1312A | HQ3398A | GI-335336994 | GI-289531305 | GI-299123798 | 2502019302 | HLAC2660 | |  |
| cHOG0624 | VNG0536 | RRNAC0496 | HVO_0819 | hbor_23980 | hmuk_2694 | huta_2026 | NP2878A | HQ1563A | GI-335336320 | GI-289529542 | GI-299126002 | 2502017898 | HLAC0657 | |  |
| cHOG0625 | VNG2312 | RRNAC2602 | HVO_0253 | hbor_29370 | hmuk_0888 | huta_1547 | NP1420A | HQ3708A | GI-335336594 | GI-289530935 | GI-299123137 | 2502015636 | HLAC0404 | |  |
| cHOG0626 | VNG1227 | RRNAC0120 | HVO_2793 | hbor_12650 | hmuk_2637 | huta_0185 | NP3086A | HQ2966A | GI-335338536 | GI-289532539 | GI-299124809 | 2502017197 | HLAC1960 | |  |
| cHOG0627 | VNG2326 | RRNAC2628 | HVO_0081 | hbor_01390 | hmuk_0914 | huta_1970 | NP1246A | HQ3447A | GI-335336911 | GI-289531236 | GI-299123154 | 2502015775 | HLAC2622 | |  |
| cHOG0628 | VNG0804 | RRNAC0521 | HVO_1164 | hbor_20760 | hmuk_2568 | huta_0268 | NP2902A | HQ2504A | GI-335337949 | GI-289532333 | GI-299125243 | 2502016551 | HLAC0666 | |  |
| cHOG0629 | VNG2519 | RRNAC2217 | HVO_1824 | hbor_03830 | hmuk_0225 | huta_0964 | NP1428A | HQ1067A | GI-335338876 | GI-289530537 | GI-299126019 | 2502019072 | HLAC2163 | |  |
| cHOG0631 | VNG0274 | RRNAC1863 | HVO_0762 | hbor_24510 | hmuk_3018 | huta_1193 | NP1302A | HQ1178A | GI-335339407 | GI-289531830 | GI-299125881 | 2502016905 | HLAC0778 | |  |
| cHOG0632 | VNG2112 | RRNAC2519 | HVO_0338 | hbor_28330 | hmuk_0846 | huta_1789 | NP1320A | HQ3058A | GI-335339520 | GI-289530410 | GI-299123351 | 2502018059 | HLAC0273 | |  |
| cHOG0633 | VNG0779 | RRNAC1441 | HVO_1018 | hbor_21970 | hmuk_1907 | huta_0717 | NP3224A | HQ1581A | GI-335338091 | GI-289529854 | GI-299125204 | 2502017745 | HLAC2404 | |  |
| cHOG0634 | VNG1168 | RRNAC0258 | HVO_2747 | hbor_12150 | hmuk_2672 | huta_0622 | NP3680A | HQ2894A | GI-335339438 | GI-289530329 | GI-299124768 | 2502018023 | HLAC2103 | |  |
| cHOG0635 | VNG2305 | RRNAC2577 | HVO_2981 | hbor_02710 | hmuk_0877 | huta_1551 | NP1408A | HQ1029A | GI-335336582 | GI-289530945 | GI-299123121 | 2502015709 | HLAC2717 | |  |
| cHOG0636 | VNG1075 | RRNAC0845 | HVO_1462 | hbor_17690 | hmuk_2250 | huta_0394 | NP2734A | HQ1875A | GI-335338665 | GI-289532756 | GI-299124711 | 2502017396 | HLAC1175 | |  |
| cHOG0637 | VNG0335 | RRNAC1757 | HVO_0694 | hbor_25240 | hmuk_0850 | huta_2740 | NP1204A | HQ1260A | GI-335339260 | GI-289531800 | GI-299125663 | 2502016950 | HLAC0430 | |  |
| cHOG0638 | VNG1180 | RRNAC2024 | HVO_A0230 | hbor_37270 | hmuk_0190 | huta_2075 | NP0038A | HQ1843A | GI-335338178 | GI-289532278 | GI-299125280 | 2502014199 | HLAC0830 | |  |
| cHOG0639 | VNG2408 | RRNAC2709 | HVO_3013 | hbor_02090 | hmuk_0813 | huta_1616 | NP0592A | HQ3737A | GI-335336811 | GI-289531148 | GI-299123185 | 2502019430 | HLAC2745 | |  |
| cHOG0640 | VNG0431 | RRNAC0661 | HVO_1391 | hbor_18440 | hmuk_3070 | huta_0878 | NP4092A | HQ2391A | GI-335339322 | GI-289531963 | GI-299125824 | 2502016998 | HLAC1402 | |  |
| cHOG0641 | VNG2003 | RRNAC3188 | HVO_1852 | hbor_04540 | hmuk_1305 | huta_2002 | NP5042A | HQ1108A | GI-335338952 | GI-289530586 | GI-299123961 | 2502019017 | HLAC1665 | |  |
| cHOG0642 | VNG0368 | RRNAC0693 | HVO_0723 | hbor_24930 | hmuk_2959 | huta_2467 | NP0822A | HQ1238A | GI-335339460 | GI-289530351 | GI-299125564 | 2502018256 | HLAC1563 | |  |
| cHOG0643 | VNG1356 | RRNAC0028 | HVO_2900 | hbor_14250 | hmuk_2778 | huta_2660 | NP3778A | HQ2471A | GI-335338702 | GI-289532794 | GI-299124558 | 2502017432 | HLAC2205 | |  |
| cHOG0644 | VNG1435 | RRNAC0365 | HVO_2487 | hbor_11210 | hmuk_2563 | huta_0768 | NP1988A | HQ2908A | GI-335336504 | GI-289529280 | GI-299124456 | 2502017510 | HLAC1745 | |  |
| cHOG0645 | VNG2656 | RRNAC2415 | HVO_0355 | hbor_28150 | hmuk_0505 | huta_2873 | NP0128A | HQ3389A | GI-335337129 | GI-289531383 | GI-299125930 | 2502015959 | HLAC0408 | |  |
| cHOG0646 | VNG0289 | RRNAC1809 | HVO_0770 | hbor_24430 | hmuk_3209 | huta_2907 | NP1134A | HQ1171A | GI-335339398 | GI-289531976 | GI-299125887 | 2502016918 | HLAC0557 | |  |
| cHOG0647 | VNG0450 | RRNAC1308 | HVO_1081 | hbor_22700 | hmuk_2051 | huta_0203 | NP2066A | HQ1665A | GI-335336291 | GI-289529914 | GI-299125848 | 2502017937 | HLAC0799 | |  |
| cHOG0648 | VNG1329 | RRNAC1115 | HVO_2915 | hbor_14420 | hmuk_2543 | huta_2882 | NP4036A | HQ2458A | GI-335336109 | GI-289529695 | GI-299124582 | 2502018155 | HLAC1649 | |  |
| cHOG0649 | VNG0300 | RRNAC1911 | HVO_0781 | hbor_18120 | hmuk_3201 | huta_2899 | NP0906A | HQ1165A | GI-335339389 | GI-289532028 | GI-299125896 | 2502016472 | HLAC1036 | |  |
| cHOG0650 | VNG0854 | RRNAC0405 | HVO_1631 | hbor_15900 | hmuk_2492 | huta_2799 | NP3154A | HQ2590A | GI-335338692 | GI-289532787 | GI-299125015 | 2502017423 | HLAC0813 | |  |
| cHOG0651 | VNG0396 | RRNAC1816 | HVO_0879 | hbor_23420 | hmuk_3076 | huta_2703 | NP4060A | HQ1529A | GI-335336267 | GI-289529555 | GI-299125767 | 2502018232 | HLAC0781 | |  |
| cHOG0652g | VNG0596 | RRNAC0853 | HVO_1171 | hbor_20700 | hmuk_2324 | huta_0400 | NP4116A | HQ2496A | GI-335339066 | GI-289532409 | GI-299125296 | 2502016663 | HLAC1287 | |  |
| cHOG0655 | VNG2657 | RRNAC2423 | HVO_0354 | hbor_28160 | hmuk_0509 | huta_2384 | NP0124A | HQ3390A | GI-335337128 | GI-289531376 | GI-299125934 | 2502015953 | HLAC0100 | |  |
| cHOG0656 | VNG1827 | RRNAC3473 | HVO_2626 | hbor_09480 | hmuk_1395 | huta_0425 | NP1972A | HQ3193A | GI-335339005 | GI-289531924 | GI-299124317 | 2502016372 | HLAC2592 | |  |
| cHOG0657 | VNG2119 | RRNAC3084 | HVO_0332 | hbor_28390 | hmuk_1106 | huta_0926 | NP0990A | HQ3230A | GI-335337662 | GI-289530183 | GI-299123265 | 2502018587 | HLAC0350 | |  |
| cHOG0658 | VNG1086 | RRNAC0835 | HVO_1473 | hbor_17600 | hmuk_2537 | huta_2877 | NP2722A | HQ1870A | GI-335339077 | GI-289532420 | GI-299124720 | 2502016673 | HLAC1490 | |  |
| cHOG0659 | VNG2008 | RRNAC3180 | HVO_1857 | hbor_04590 | hmuk_1300 | huta_1982 | NP4416A | HQ1105A | GI-335338837 | GI-289530742 | GI-299123906 | 2502019132 | HLAC2216 | |  |
| cHOG0660 | VNG1707 | RRNAC1597 | HVO_2549 | hbor_10380 | hmuk_1843 | huta_2307 | NP4884A | HQ2827A | GI-335336414 | GI-289529466 | GI-299124073 | 2502017863 | HLAC2434 | |  |
| cHOG0661 | VNG2082 | RRNAC2796 | HVO_0444 | hbor_27510 | hmuk_0717 | huta_1768 | NP0084A | HQ1380A | GI-335337407 | GI-289530675 | GI-299123783 | 2502018800 | HLAC0379 | |  |
| cHOG0662 | VNG2576 | RRNAC2156 | HVO_1950 | hbor_05450 | hmuk_0600 | huta_1405 | NP0396A | HQ3165A | GI-335338769 | GI-289530820 | GI-299125695 | 2502019230 | HLAC2399 | |  |
| cHOG0663 | VNG1616 | RRNAC1742 | HVO_2421 | hbor_08540 | hmuk_1744 | huta_2767 | NP4796A | HQ2869A | GI-335336204 | GI-289529955 | GI-299124394 | 2502017681 | HLAC1971 | |  |
| cHOG0664 | VNG2584 | RRNAC2445 | HVO_1946 | hbor_05410 | hmuk_0627 | huta_2534 | NP0388A | HQ3162A | GI-335338773 | GI-289530814 | GI-299125699 | 2502019226 | HLAC2078 | |  |
| cHOG0665 | VNG1681 | RRNAC0318 | HVO_2526 | hbor_10670 | hmuk_1362 | huta_2375 | NP4768A | HQ2861A | GI-335337606 | GI-289530236 | GI-299124238 | 2502018342 | HLAC1899 | |  |
| cHOG0666 | VNG1093 | RRNAC1289 | HVO_1128 | hbor_21160 | hmuk_2179 | huta_1760 | NP1588A | HQ1656A | GI-335339070 | GI-289532411 | GI-299124735 | 2502016666 | HLAC0262 | |  |
| cHOG0667 | VNG1895 | RRNAC3398 | HVO_2368 | hbor_07990 | hmuk_1656 | huta_1376 | NP3214A | HQ3069A | GI-335337306 | GI-289531488 | GI-299124378 | 2502016222 | HLAC1978 | |  |
| cHOG0669g | VNG2613 | RRNAC2102 | HVO_0423 | hbor_27700 | hmuk_0310 | huta_0334 | NP0676A | HQ1371A | GI-335338813 | GI-289530773 | GI-299125729 | 2502019150 | HLAC0396 | |  |
| cHOG0670 | VNG1623 | RRNAC1751 | HVO_2433 | hbor_08720 | hmuk_1756 | huta_3013 | NP1770A | HQ2733A | GI-335338112 | GI-289529870 | GI-299124386 | 2502017719 | HLAC1803 | |  |
| cHOG0671 | VNG1079 | RRNAC0841 | HVO_1465 | hbor_17670 | hmuk_2252 | huta_0395 | NP2730A | HQ1874A | GI-335338668 | GI-289532760 | GI-299124714 | 2502017400 | HLAC2150 | |  |
| cHOG0672 | VNG0403 | RRNAC0166 | HVO_0870 | hbor_23500 | hmuk_3132 | huta_2695 | NP1796A | HQ1715A | GI-335339172 | GI-289531737 | GI-299125777 | 2502018845 | HLAC0777 | |  |
| cHOG0673 | VNG1429 | RRNAC0051 | HVO_2479 | hbor_11120 | hmuk_2581 | huta_0762 | NP2028A | HQ3012A | GI-335338086 | GI-289529848 | GI-299124451 | 2502017518 | HLAC2334 | |  |
| cHOG0674 | VNG1308 | RRNAC1093 | HVO_2809 | hbor_12880 | hmuk_2078 | huta_2833 | NP4266A | HQ2995A | GI-335337954 | GI-289532328 | GI-299124818 | 2502016546 | HLAC1989 | |  |
| cHOG0675 | VNG2514 | RRNAC2212 | HVO_1827 | hbor_03790 | hmuk_0255 | huta_0937 | NP1368A | HQ1070A | GI-335338910 | GI-289530546 | GI-299126023 | 2502019053 | HLAC2128 | |  |
| cHOG0676 | VNG1230 | RRNAC0124 | HVO_1306 | hbor_19190 | hmuk_2640 | huta_0181 | NP3082A | HQ3357A | GI-335338202 | GI-289531597 | GI-299124665 | 2502017051 | HLAC1114 | |  |
| cHOG0677 | VNG1279 | RRNAC1175 | HVO_1092 | hbor_22810 | hmuk_2400 | huta_2801 | NP3736A | HQ1515A | GI-335338507 | GI-289532511 | GI-299124914 | 2502017326 | HLAC0962 | |  |
| cHOG0678 | VNG0097 | RRNAC2361 | HVO_0482 | hbor_27030 | hmuk_0658 | huta_2547 | NP0010A | HQ1393A | GI-335337033 | GI-289531358 | GI-299125954 | 2502015876 | HLAC2370 | |  |
| cHOG0679 | VNG2051 | RRNAC2830 | HVO_1898 | hbor_05030 | hmuk_0727 | huta_2206 | NP5078A | HQ1338A | GI-335338862 | GI-289530714 | GI-299123832 | 2502019101 | HLAC2395 | |  |
| cHOG0680 | VNG2648 | RRNAC2405 | HVO_0360 | hbor_28100 | hmuk_0496 | huta_0810 | NP0306A | HQ3384A | GI-335339189 | GI-289531726 | GI-299126074 | 2502018711 | HLAC0157 | |  |
| cHOG0681 | VNG0433 | RRNAC0660 | HVO_1392 | hbor_18430 | hmuk_3072 | huta_0879 | NP4090A | HQ2743A | GI-335339323 | GI-289531964 | GI-299125826 | 2502016997 | HLAC1403 | |  |
| cHOG0682 | VNG1783 | RRNAC1724 | HVO_2323 | hbor_07360 | hmuk_1542 | huta_0200 | NP4536A | HQ3327A | GI-335337499 | GI-289530462 | GI-299123888 | 2502018904 | HLAC2134 | |  |
| cHOG0683 | VNG1390 | RRNAC0869 | HVO_1664 | hbor_15480 | hmuk_2242 | huta_0402 | NP3718A | HQ2564A | GI-335337900 | GI-289529738 | GI-299124610 | 2502018104 | HLAC1043 | |  |
| cHOG0684 | VNG1916 | RRNAC3300 | HVO_2218 | hbor_06570 | hmuk_1593 | huta_1763 | NP3176A | HQ3124A | GI-335337259 | GI-289532118 | GI-299123777 | 2502016130 | HLAC1882 | |  |
| cHOG0685 | VNG0500 | RRNAC0629 | HVO_1593 | hbor_16220 | hmuk_2829 | huta_0342 | NP4074A | HQ2638A | GI-335339559 | GI-289529901 | GI-299125529 | 2502017659 | HLAC1486 | |  |
| cHOG0686 | VNG1310 | RRNAC1091 | HVO_2811 | hbor_12900 | hmuk_2076 | huta_2835 | NP4270A | HQ2997A | GI-335337956 | GI-289532326 | GI-299124820 | 2502016544 | HLAC1987 | |  |
| cHOG0687 | VNG1289 | RRNAC1179 | HVO_1248 | hbor_19810 | hmuk_2395 | huta_0524 | NP3526A | HQ1807A | GI-335339656 | GI-289529824 | GI-299124641 | 2502017557 | HLAC0946 | |  |
| cHOG0688 | VNG0938 | RRNAC3110 | HVO_1003 | hbor_21230 | hmuk_1236 | huta_1001 | NP4402A | HQ2238A | GI-335338392 | GI-299125546 | GI-289530103 | 2502018723 | HLAC0130 | |  |
| cHOG0689g | VNG2400 | RRNAC2575 | HVO_0017 | hbor_02040 | hmuk_1130 | huta_2252 | NP1236A | HQ3734A | GI-335336808 | GI-289531145 | GI-299123181 | 2502019425 | HLAC2740 | |  |
| cHOG0690 | VNG2467 | RRNAC3113 | HVO_0116 | hbor_01070 | hmuk_1233 | huta_0999 | NP1014A | HQ3423A | GI-335336689 | GI-289531042 | GI-299123336 | 2502015824 | HLAC0827 | |  |
| cHOG0691 | VNG1176 | RRNAC0865 | HVO_1669 | hbor_15440 | hmuk_2238 | huta_0406 | NP2062A | HQ2567A | GI-335336307 | GI-289529928 | GI-299124605 | 2502017713 | HLAC1042 | |  |
| cHOG0692 | VNG1157 | RRNAC0103 | HVO_2737 | hbor_12060 | hmuk_2621 | huta_0750 | NP3660A | HQ2885A | GI-335336117 | GI-289529688 | GI-299124779 | 2502018164 | HLAC1842 | |  |
| cHOG0693 | VNG2043 | RRNAC2493 | HVO_1893 | hbor_04980 | hmuk_1176 | huta_1510 | NP4426A | HQ1343A | GI-335337485 | GI-289530506 | GI-299123942 | 2502018693 | HLAC2200 | |  |
| cHOG0694 | VNG2088 | RRNAC2788 | HVO_0236 | hbor_29540 | hmuk_0713 | huta_1529 | NP0194A | HQ3646A | GI-335337413 | GI-289530683 | GI-299123788 | 2502018794 | HLAC2358 | |  |
| cHOG0695 | VNG2612 | RRNAC2103 | HVO_0424 | hbor_27690 | hmuk_0309 | huta_0333 | NP0678A | HQ1372A | GI-335338812 | GI-289530774 | GI-299125728 | 2502019149 | HLAC0397 | |  |
| cHOG0696 | VNG1989 | RRNAC3212 | HVO_1973 | hbor_05680 | hmuk_1320 | huta_2686 | NP4994A | HQ3099A | GI-335338993 | GI-289531915 | GI-299123974 | 2502016357 | HLAC1961 | |  |
| cHOG0697 | VNG1944 | RRNAC3269 | HVO_2189 | hbor_06290 | hmuk_1425 | huta_2231 | NP5008A | HQ3130A | GI-335337096 | GI-289531858 | GI-299124029 | 2502016264 | HLAC1250 | |  |
| cHOG0698 | VNG2472 | RRNAC3102 | HVO_0107 | hbor_01150 | hmuk_1252 | huta_1226 | NP0940A | HQ3428A | GI-335336674 | GI-289531056 | GI-299123326 | 2502015809 | HLAC2247 | |  |
| cHOG0699 | VNG6311 | RRNAC1224 | HVO_1455 | hbor_17820 | hmuk_2302 | huta_0521 | NP3520A | HQ1878A | GI-335338568 | GI-289532665 | GI-299124636 | 2502017283 | HLAC1284 | |  |
| cHOG0700 | VNG1170 | RRNAC0260 | HVO_2749 | hbor_12170 | hmuk_2674 | huta_0624 | NP3684A | HQ2896A | GI-335339440 | GI-289530331 | GI-299124766 | 2502018021 | HLAC2277 | |  |
| cHOG0701 | VNG1670 | RRNAC1512 | HVO_2372 | hbor_08030 | hmuk_1798 | huta_0919 | NP1728A | HQ3310A | GI-335336453 | GI-289529427 | GI-299123898 | 2502017811 | HLAC2292 | |  |
| cHOG0702 | VNG1688 | RRNAC1612 | HVO_2565 | hbor_10220 | hmuk_1827 | huta_2291 | NP4852A | HQ2843A | GI-335336430 | GI-289529450 | GI-299124057 | 2502017847 | HLAC2450 | |  |
| cHOG0703 | VNG1714 | RRNAC1593 | HVO_2545 | hbor_10420 | hmuk_1847 | huta_2311 | NP4892A | HQ2823A | GI-335336410 | GI-289529470 | GI-299124077 | 2502017867 | HLAC2430 | |  |
| cHOG0704 | VNG0879 | RRNAC0441 | HVO_1560 | hbor_16680 | hmuk_2742 | huta_2774 | NP3468A | HQ2663A | GI-335338549 | GI-289532552 | GI-299125409 | 2502017183 | HLAC0869 | |  |
| cHOG0705 | VNG2599 | RRNAC2111 | HVO_0437 | hbor_27570 | hmuk_0313 | huta_0325 | NP1308A | HQ1302A | GI-335338800 | GI-289530789 | GI-299125717 | 2502019187 | HLAC0371 | |  |
| cHOG0706 | VNG2586 | RRNAC2436 | HVO_1936 | hbor_05320 | hmuk_0342 | huta_1396 | NP1054A | HQ3153A | GI-335338782 | GI-289530806 | GI-299125702 | 2502019217 | HLAC1679 | |  |
| cHOG0707 | VNG2669 | RRNAC1407 | HVO_0915 | hbor_23090 | hmuk_1952 | huta_0244 | NP1872A | HQ1604A | GI-335339232 | GI-289531778 | GI-299125962 | 2502016925 | HLAC0898 | |  |
| cHOG0708 | VNG2600 | RRNAC2110 | HVO_0438 | hbor_27560 | hmuk_0312 | huta_0326 | NP1310A | HQ1323A | GI-335338801 | GI-289530788 | GI-299125718 | 2502019186 | HLAC0372 | |  |
| cHOG0709 | VNG2010 | RRNAC3179 | HVO_1858 | hbor_04600 | hmuk_1299 | huta_1981 | NP2282A | HQ1104A | GI-335338838 | GI-289530741 | GI-299123907 | 2502019131 | HLAC2312 | |  |
| cHOG0710 | VNG0293 | RRNAC1811 | HVO_0772 | hbor_24410 | hmuk_3207 | huta_2906 | NP1176A | HQ1169A | GI-335339396 | GI-289532035 | GI-299125889 | 2502016920 | HLAC0549 | |  |
| cHOG0711 | VNG1120 | RRNAC0776 | HVO_1712 | hbor_14960 | hmuk_3113 | huta_0588 | NP2750A | HQ2538A | GI-335338183 | GI-289531566 | GI-299124671 | 2502017064 | HLAC1518 | |  |
| cHOG0712 | VNG0777 | RRNAC1438 | HVO_1015 | hbor_21940 | hmuk_1909 | huta_0715 | NP3220A | HQ1579A | GI-335338095 | GI-289529856 | GI-299125202 | 2502017742 | HLAC2406 | |  |
| cHOG0713 | VNG1252 | RRNAC1137 | HVO_1336 | hbor_18990 | hmuk_2420 | huta_0546 | NP3964A | HQ2403A | GI-335338067 | GI-289532285 | GI-299124927 | 2502014191 | HLAC0625 | |  |
| cHOG0715 | VNG2537 | RRNAC2236 | HVO_1809 | hbor_04000 | hmuk_0202 | huta_0972 | NP1170A | HQ1060A | GI-335337162 | GI-289531414 | GI-299126006 | 2502015993 | HLAC2473 | |  |
| cHOG0716 | VNG1297 | RRNAC1233 | HVO_1649 | hbor_15700 | hmuk_2306 | huta_0513 | NP3100A | HQ2584A | GI-335338563 | GI-289532660 | GI-299124630 | 2502017289 | HLAC1394 | |  |
| cHOG0717 | VNG0546 | RRNAC3510 | HVO_0697 | hbor_25200 | hmuk_2882 | huta_2792 | NP1624A | HQ1257A | GI-335339293 | GI-289531713 | GI-299125461 | 2502016478 | HLAC1007 | |  |
| cHOG0718 | VNG0499 | RRNAC0628 | HVO_1594 | hbor_16210 | hmuk_2830 | huta_0341 | NP4072A | HQ2637A | GI-335339561 | GI-289529900 | GI-299125532 | 2502017658 | HLAC1485 | |  |
| cHOG0719 | VNG1381 | RRNAC2893 | HVO_0093 | hbor_01290 | hmuk_1071 | huta_2973 | NP0698A | HQ3439A | GI-335336921 | GI-289531246 | GI-299123300 | 2502015787 | HLAC0011 | |  |
| cHOG0720 | VNG1998 | RRNAC3200 | HVO_1960 | hbor_05550 | hmuk_1310 | huta_2600 | NP4482A | HQ3073A | GI-335337227 | GI-289530592 | GI-299123967 | 2502019006 | HLAC2009 | |  |
| cHOG0721 | VNG2515 | RRNAC2214 | HVO_1825 | hbor_03810 | hmuk_0227 | huta_0962 | NP1370A | HQ1068A | GI-335338879 | GI-289530549 | GI-299126021 | 2502019068 | HLAC2548 | |  |
| cHOG0722 | VNG2668 | RRNAC2432 | HVO_0346 | hbor_28250 | hmuk_0516 | huta_0785 | NP0108A | HQ3397A | GI-335337121 | GI-289531367 | GI-299125942 | 2502015944 | HLAC0108 | |  |
| cHOG0723 | VNG0245 | RRNAC1776 | HVO_0661 | hbor_25490 | hmuk_0021 | huta_2714 | NP5166A | HQ1278A | GI-335338231 | GI-289531622 | GI-299125631 | 2502017087 | HLAC0445 | |  |
| cHOG0724 | VNG0906 | RRNAC0469 | HVO_1037 | hbor_22170 | hmuk_2719 | huta_2813 | NP3104A | HQ1698A | GI-335339080 | GI-289532422 | GI-299125253 | 2502016676 | HLAC1033 | |  |
| cHOG0725 | VNG2295 | RRNAC2555 | HVO_2986 | hbor_02590 | hmuk_0863 | huta_2124 | NP2258A | HQ1026A | GI-335336569 | GI-289530953 | GI-299123112 | 2502015697 | HLAC2701 | |  |
| cHOG0726 | VNG2048 | RRNAC2488 | HVO_1896 | hbor_05010 | hmuk_1171 | huta_1956 | NP5074A | HQ1340A | GI-335337479 | GI-289530500 | GI-299123946 | 2502018700 | HLAC2393 | |  |
| cHOG0727 | VNG1220 | RRNAC0117 | HVO_2796 | hbor_12670 | hmuk_2634 | huta_0669 | NP4144A | HQ2973A | GI-335338525 | GI-289532529 | GI-299124811 | 2502017215 | HLAC1838 | |  |
| cHOG0728g | VNG1309 | RRNAC1092 | HVO_2810 | hbor_12890 | hmuk_2077 | huta_2834 | NP4268A | HQ2996A | GI-335337955 | GI-289532327 | GI-299124819 | 2502016545 | HLAC1988 | |  |
| cHOG0729 | VNG0757 | RRNAC0861 | HVO_1174 | hbor_20670 | hmuk_2330 | huta_0285 | NP4106A | HQ2491A | GI-335337927 | GI-289532358 | GI-299125307 | 2502016577 | HLAC1708 | |  |
| cHOG0730 | VNG0429 | RRNAC0664 | HVO_1388 | hbor_18470 | hmuk_3067 | huta_0873 | NP0370A | HQ2393A | GI-335339041 | GI-289531960 | GI-299125822 | 2502017000 | HLAC0836 | |  |
| cHOG0732g | VNG0227 | RRNAC1691 | HVO_0645 | hbor_25650 | hmuk_0026 | huta_2427 | NP0628A | HQ1291A | GI-335336094 | GI-289529714 | GI-299125607 | 2502018127 | HLAC0257 | |  |
| cHOG0733 | VNG1244 | RRNAC0752 | HVO_1322 | hbor_19090 | hmuk_2349 | huta_3024 | NP3980A | HQ2763A | GI-335338363 | GI-289532596 | GI-299124919 | 2502014131 | HLAC1875 | |  |
| cHOG0734 | VNG0890 | RRNAC0451 | HVO_1577 | hbor_16490 | hmuk_2734 | huta_2771 | NP3506A | HQ2649A | GI-335336319 | GI-289529544 | GI-299125258 | 2502017900 | HLAC0936 | |  |
| cHOG0736 | VNG0375 | RRNAC0700 | HVO_0718 | hbor_24990 | hmuk_2937 | huta_0894 | NP0812A | HQ1242A | GI-335339481 | GI-289530373 | GI-299125570 | 2502018281 | HLAC0900 | |  |
| cHOG0737 | VNG0099 | RRNAC2357 | HVO_0484 | hbor_27050 | hmuk_0653 | huta_2544 | NP0416A | HQ1391A | GI-335337030 | GI-289531355 | GI-299125959 | 2502015878 | HLAC0057 | |  |
| cHOG0738 | VNG0982 | RRNAC0743 | HVO_1691 | hbor_15200 | hmuk_2388 | huta_0396 | NP3706A | HQ2582A | GI-335338588 | GI-289532673 | GI-299124947 | 2502017259 | HLAC1399 | |  |
| cHOG0739g | VNG2508 | RRNAC2971 | HVO_2941 | hbor_03160 | hmuk_0660 | huta_1680 | NP0424A | HQ1054A | GI-335336549 | GI-289530925 | GI-299123068 | 2502019277 | HLAC0021 | |  |
| cHOG0740 | VNG1700 | RRNAC1603 | HVO_2555 | hbor_10320 | hmuk_1837 | huta_2301 | NP4872A | HQ2833A | GI-335336420 | GI-289529460 | GI-299124067 | 2502017857 | HLAC2440 | |  |
| cHOG0741 | VNG0860 | RRNAC1396 | HVO_1042 | hbor_22240 | hmuk_1961 | huta_0627 | NP4162A | HQ1696A | GI-335338682 | GI-289532776 | GI-299125022 | 2502017413 | HLAC0620 | |  |
| cHOG0742 | VNG2644 | RRNAC2085 | HVO_0392 | hbor_28000 | hmuk_0618 | huta_0144 | NP0342A | HQ3071A | GI-335337355 | GI-289530704 | GI-299126065 | 2502018685 | HLAC0118 | |  |
| cHOG0743 | VNG2469 | RRNAC3112 | HVO_0115 | hbor_01080 | hmuk_1234 | huta_1000 | NP1012A | HQ3424A | GI-335336688 | GI-289531043 | GI-299123335 | 2502015823 | HLAC0828 | |  |
| cHOG0744 | VNG1777 | RRNAC1713 | HVO_2314 | hbor_07300 | hmuk_1551 | huta_2826 | NP4554A | HQ3333A | GI-335337508 | GI-289530454 | GI-299124277 | 2502018895 | HLAC2001 | |  |
| cHOG0745 | VNG1108 | RRNAC1414 | HVO_2758 | hbor_12260 | hmuk_2187 | huta_0252 | NP4454A | HQ2922A | GI-335338618 | GI-289532709 | GI-299124753 | 2502017131 | HLAC1983 | |  |
| cHOG0746 | VNG1403 | RRNAC1065 | HVO_2850 | hbor_13610 | hmuk_2230 | huta_0413 | NP3442A | HQ2999A | GI-335338331 | GI-289532565 | GI-299124831 | 2502017371 | HLAC1478 | |  |
| cHOG0747 | VNG2641 | RRNAC2078 | HVO_0398 | hbor_27930 | hmuk_0613 | huta_0147 | NP0434A | HQ3111A | GI-335338853 | GI-289530727 | GI-299125754 | 2502019116 | HLAC0333 | |  |
| cHOG0748 | VNG1367 | RRNAC0041 | HVO_1109 | hbor_22970 | hmuk_2790 | huta_2505 | NP3812A | HQ1503A | GI-335338718 | GI-289532803 | GI-299124688 | 2502017449 | HLAC0717 | |  |
| cHOG0749 | VNG0179 | RRNAC2036 | HVO_0569 | hbor_26420 | hmuk_0196 | huta_2401 | NP0248A | HQ1420A | GI-335337648 | GI-289530199 | GI-299125759 | 2502018443 | HLAC0533 | |  |
| cHOG0750 | VNG0550 | RRNAC3513 | HVO_0700 | hbor_25170 | hmuk_2879 | huta_2789 | NP1616A | HQ1254A | GI-335339296 | GI-289531710 | GI-299125458 | 2502016481 | HLAC1010 | |  |
| cHOG0751 | VNG1809 | RRNAC3357 | HVO_2359 | hbor_07860 | hmuk_1634 | huta_0066 | NP4510A | HQ3312A | GI-335339227 | GI-289531773 | GI-299124300 | 2502018944 | HLAC1717 | |  |
| cHOG0752 | VNG1138 | RRNAC0065 | HVO_2778 | hbor_12510 | hmuk_2594 | huta_2519 | NP2838A | HQ2939A | GI-335336139 | GI-289529662 | GI-299124792 | 2502018186 | HLAC1821 | |  |
| cHOG0753 | VNG1695 | RRNAC1606 | HVO_2559 | hbor_10280 | hmuk_1833 | huta_2297 | NP4864A | HQ2837A | GI-335336424 | GI-289529456 | GI-299124063 | 2502017853 | HLAC2444 | |  |
| cHOG0754 | VNG0599 | RRNAC1349 | HVO_1167 | hbor_20730 | hmuk_1998 | huta_0089 | NP4480A | HQ2501A | GI-335338267 | GI-289532399 | GI-299125294 | 2502016657 | HLAC0943 | |  |
| cHOG0755 | VNG2284 | RRNAC2524 | HVO_2992 | hbor_02530 | hmuk_0854 | huta_1781 | NP5154A | HQ1022A | GI-335336603 | GI-289531115 | GI-299123102 | 2502015669 | HLAC2182 | |  |
| cHOG0756 | VNG0677 | RRNAC0767 | HVO_1142 | hbor_20990 | hmuk_2905 | huta_2970 | NP1676A | HQ2355A | GI-335338013 | GI-289531515 | GI-299125340 | 2502016794 | HLAC1177 | |  |
| cHOG0757 | VNG1691 | RRNAC1609 | HVO_2562 | hbor_10250 | hmuk_1830 | huta_2294 | NP4858A | HQ2840A | GI-335336427 | GI-289529453 | GI-299124060 | 2502017850 | HLAC2447 | |  |
| cHOG0758 | VNG1668 | RRNAC1511 | HVO_2373 | hbor_08040 | hmuk_1797 | huta_0917 | NP4798A | HQ3309A | GI-335339662 | GI-289529734 | GI-299123897 | 2502018109 | HLAC1967 | |  |
| cHOG0759 | VNG0769 | RRNAC0811 | HVO_1187 | hbor_20550 | hmuk_2340 | huta_0002 | NP0376A | HQ2349A | GI-335338049 | GI-289531543 | GI-299125321 | 2502017351 | HLAC0690 | |  |
| cHOG0760 | VNG0790 | RRNAC1426 | HVO_1148 | hbor_20930 | hmuk_2134 | huta_0725 | NP3204A | HQ2361A | GI-335338223 | GI-289531612 | GI-299125212 | 2502017017 | HLAC0615 | |  |
| cHOG0761g | VNG1384 | RRNAC0432 | HVO_1535 | hbor_16950 | hmuk_2751 | huta_2812 | NP2692A | HQ2677A | GI-335338616 | GI-289532707 | GI-299125186 | 2502017129 | HLAC0911 | |  |
| cHOG0762 | VNG0896 | RRNAC0446 | HVO_1581 | hbor_16430 | hmuk_2739 | huta_1386 | NP3512A | HQ2646A | GI-335338148 | GI-289529747 | GI-299125255 | 2502018095 | HLAC0939 | |  |
| cHOG0763 | VNG2274 | RRNAC2569 | HVO_0198 | hbor_00510 | hmuk_1139 | huta_2257 | NP0776A | HQ3675A | GI-335336842 | GI-289531172 | GI-299123277 | 2502019405 | HLAC2636 | |  |
| cHOG0764 | VNG2234 | RRNAC2474 | HVO_0136 | hbor_00870 | hmuk_0702 | huta_1606 | NP1258A | HQ3399A | GI-335336635 | GI-289531095 | GI-299125250 | 2502015647 | HLAC2368 | |  |
| cHOG0765g | VNG1640 | RRNAC1628 | HVO_2518 | hbor_08970 | hmuk_1760 | huta_1293 | NP3352A | HQ2783A | GI-335336489 | GI-289529303 | GI-299123808 | 2502017759 | HLAC1936 | |  |
| cHOG0766 | VNG1995 | RRNAC3205 | HVO_1964 | hbor_05600 | hmuk_1314 | huta_2681 | NP4984A | HQ3075A | GI-335338997 | GI-289531919 | GI-299123969 | 2502016364 | HLAC2061 | |  |
| cHOG0767 | VNG1768 | RRNAC1929 | HVO_2300 | hbor_07140 | hmuk_1604 | huta_2581 | NP4752A | HQ3348A | GI-335337526 | GI-289530438 | GI-299124262 | 2502018749 | HLAC1578 | |  |
| cHOG0768 | VNG2296 | RRNAC2554 | HVO_2984 | hbor_02610 | hmuk_0864 | huta_2125 | NP1404A | HQ1027A | GI-335336571 | GI-289530951 | GI-299123113 | 2502015699 | HLAC2625 | |  |
| cHOG0769 | VNG0127 | RRNAC2306 | HVO_0510 | hbor_27270 | hmuk_0577 | huta_1827 | NP0408A | HQ1136A | GI-335337193 | GI-289531460 | GI-299125975 | 2502016034 | HLAC0062 | |  |
| cHOG0770 | VNG1433 | RRNAC0055 | HVO_2475 | hbor_11080 | hmuk_2585 | huta_0766 | NP1992A | HQ3016A | GI-335336502 | GI-289529282 | GI-299124455 | 2502017513 | HLAC2338 | |  |
| cHOG0771 | VNG2658 | RRNAC2424 | HVO_0353 | hbor_28170 | hmuk_0510 | huta_2383 | NP0122A | HQ3391A | GI-335337127 | GI-289531375 | GI-299125935 | 2502015952 | HLAC0101 | |  |
| cHOG0772 | VNG1701 | RRNAC1602 | HVO_2554 | hbor_10330 | hmuk_1838 | huta_2302 | NP4874A | HQ2832A | GI-335336419 | GI-289529461 | GI-299124068 | 2502017858 | HLAC2439 | |  |
| cHOG0773 | VNG0237 | RRNAC1668 | HVO_0653 | hbor_25560 | hmuk_0013 | huta_2673 | NP5234A | HQ1284A | GI-335339060 | GI-289531661 | GI-299125614 | 2502017099 | HLAC0565 | |  |
| cHOG0774 | VNG1715 | RRNAC1592 | HVO_2544 | hbor_10430 | hmuk_1848 | huta_2312 | NP4894A | HQ2822A | GI-335336409 | GI-289529471 | GI-299124078 | 2502017868 | HLAC2429 | |  |
| cHOG0775 | VNG0635 | RRNAC1447 | HVO_0978 | hbor_21470 | hmuk_1917 | huta_0706 | NP2292A | HQ1637A | GI-335339202 | GI-289532454 | GI-299125383 | 2502016731 | HLAC0704 | |  |
| cHOG0776 | VNG2610 | RRNAC2105 | HVO_0429 | hbor_27660 | hmuk_0307 | huta_0329 | NP0774A | HQ1374A | GI-335338810 | GI-289530777 | GI-299125724 | 2502019148 | HLAC0334 | |  |
| cHOG0777 | VNG0641 | RRNAC1452 | HVO_0983 | hbor_21420 | hmuk_1922 | huta_0697 | NP2304A | HQ1643A | GI-335339207 | GI-289532449 | GI-299125378 | 2502016726 | HLAC0709 | |  |
| cHOG0778 | VNG1401 | RRNAC1064 | HVO_2851 | hbor_13620 | hmuk_2227 | huta_0414 | NP3406A | HQ3004A | GI-335338330 | GI-289532564 | GI-299124832 | 2502017372 | HLAC2319 | |  |
| cHOG0779 | VNG1141 | RRNAC0068 | HVO_2775 | hbor_12480 | hmuk_2597 | huta_2516 | NP2844A | HQ2936A | GI-335336136 | GI-289529665 | GI-299124789 | 2502018183 | HLAC1824 | |  |
| cHOG0780 | VNG0508 | RRNAC0928 | HVO_1611 | hbor_16100 | hmuk_2811 | huta_1485 | NP1672A | HQ2626A | GI-335336086 | GI-289529717 | GI-299125489 | 2502018121 | HLAC1786 | |  |
| cHOG0911 | VNG1002 | RRNAC0749 | HVO_1274 | hbor_19530 | hmuk_2359 | huta_2783 | NP3380A | HQ2384A | GI-335339641 | GI-289529886 | GI-299124941 | 2502017572 | HLAC1210 | |  |
| cHOG0912 | VNG1001 | RRNAC0748 | HVO_1273 | hbor_19570 | hmuk_2358 | huta_2784 | NP3384A | HQ2382A | GI-335339645 | GI-289529887 | GI-299124942 | 2502017571 | HLAC1209 | |  |
| cHOG0913 | VNG1862 | RRNAC3425 | HVO_2602 | hbor_09740 | hmuk_1513 | huta_0076 | NP3196A | HQ3206A | GI-335337137 | GI-289531400 | GI-299124355 | 2502015968 | HLAC1199 | |  |
| cHOG0914 | VNG0424 | RRNAC0214 | HVO_1382 | hbor_18540 | hmuk_3183 | huta_0227 | NP0354A | HQ2399A | GI-335339035 | GI-289531955 | GI-299125811 | 2502016409 | HLAC1390 | |  |
| cHOG0919 | VNG2604 | RRNAC1782 | HVO_0665 | hbor_25460 | hmuk_0002 | huta_2718 | NP5174A | HQ1276A | GI-335338240 | GI-289531629 | GI-299125636 | 2502017079 | HLAC2980 | |  |
| cHOG0923 | VNG2470 | RRNAC3107 | HVO_0111 | hbor_01130 | hmuk_1241 | huta_1004 | NP0996A | HQ3426A | GI-335336683 | GI-289531050 | GI-299123331 | 2502015815 | HLAC2689 | |  |
| cHOG0925 | VNG1003 | RRNAC0750 | HVO_1276 | hbor_19520 | hmuk_2360 | huta_2782 | NP3378A | HQ2385A | GI-335336279 | GI-289529973 | GI-299124940 | 2502017692 | HLAC1211 | |  |
| cHOG0927 | VNG1046 | RRNAC0328 | HVO_1512 | hbor_17270 | hmuk_2650 | huta_0660 | NP4202A | HQ2699A | GI-335337769 | GI-289530142 | GI-299125157 | 2502018413 | HLAC1776 | |  |
| cHOG0930 | VNG0534 | RRNAC1822 | HVO_0867 | hbor_23530 | hmuk_3081 | huta_0344 | NP1636A | HQ1712A | GI-335339047 | GI-289531679 | GI-299125468 | 2502016512 | HLAC1197 | |  |
| cHOG0932 | VNG1302 | RRNAC1112 | HVO_1655 | hbor_15610 | hmuk_2311 | huta_0508 | NP3114A | HQ2557A | GI-335338478 | GI-289532194 | GI-299124625 | 2502017295 | HLAC1359 | |  |
| cHOG0933 | VNG1746 | RRNAC0485 | HVO_0959 | hbor_21740 | hmuk_2704 | huta_0115 | NP2788A | HQ1500A | GI-335337482 | GI-289531323 | GI-299123423 | 2502019252 | HLAC1200 | |  |
| cHOG0934 | VNG0601 | RRNAC1348 | HVO_1166 | hbor_20740 | hmuk_1999 | huta_0090 | NP4478A | HQ2502A | GI-335338268 | GI-289532398 | GI-299125293 | 2502016656 | HLAC1188 | |  |
| cHOG0977 | VNG0194 | RRNAC2002 | HVO_0582 | hbor_26240 | hmuk_0168 | huta_2407 | NP5326A | HQ1414A | GI-335337628 | GI-289530214 | GI-299125591 | 2502018466 | HLAC0524 | |  |
| cHOG1011 | VNG0548 | RRNAC3511 | HVO_0698 | hbor_25190 | hmuk_2881 | huta_2791 | NP1620A | HQ1256A | GI-335339294 | GI-289531712 | GI-299125460 | 2502016479 | HLAC1008 | |  |
