# Supplementary material for: Amino Acid Substitutions in Cold-Adapted Proteins from Halorubrum lacusprofundi, an Extremely Halophilic Microbe from Antarctica
Source: PLoS One. 2013 Mar 11;8(3):e58587. doi: 10.1371/journal.pone.0058587 (PMC3594186; doi:10.1371/journal.pone.0058587)
Supplement: Table S2 — Genome-wide tally and percent of amino acid substitutions between invariant mesophilic haloarchaeal proteins (vertical) and H. lacusprofundi (horizontal) for 604 selected cHOGs. (DOC) [file pone.0058587.s002.doc]

Table S2. Genome-wide tally and percent of amino acid substitutions between invariant mesophilic haloarchaeal proteins (vertical) and *H. lacusprofundi* (horizontal) for 604 selected cHOGs.

|  | Total | AA | AA | AA | AA | AA | AA | AA | AA | AA | AA | AA | AA | AA | AA | AA | AA | AA | AA | AA | AA | Substituted |
| --- | --- | --- | --- | --- | --- | --- | --- | --- | --- | --- | --- | --- | --- | --- | --- | --- | --- | --- | --- | --- | --- | --- |
|  | Number | Tally | Tally | Tally | Tally | Tally | Tally | Tally | Tally | Tally | Tally | Tally | Tally | Tally | Tally | Tally | Tally | Tally | Tally | Tally | Tally | Tally |
|  | Of | % total | % total | % total | % total | % total | % total | % total | % total | % total | % total | % total | % total | % total | % total | % total | % total | % total | % total | % total | % total | % total |
|  | Residues | % AA | % AA | % AA | % AA | % AA | % AA | % AA | % AA | % AA | % AA | % AA | % AA | % AA | % AA | % AA | % AA | % AA | % AA | % AA | % AA | % AA |
|  | Total | D | E | N | G | A | S | T | H | V | R | P | Y | W | F | M | L | I | C | Q | K | Total |
| D | 4045 | 3940 | 39 | 18 | 17 | 12 | 9 | 5 | 3 | 1 | 1 | 0 | 0 | 0 | 0 | 0 | 0 | 0 | 0 | 0 | 0 | 105 |
|  |  | 5.58 | 0.70 | 0.32 | 0.31 | 0.22 | 0.16 | 0.09 | 0.05 | 0.02 | 0.02 | 0.00 | 0.00 | 0.00 | 0.00 | 0.00 | 0.00 | 0.00 | 0.00 | 0.00 | 0.00 | 1.89 |
|  |  | 97.40 | 0.96 | 0.44 | 0.42 | 0.30 | 0.22 | 0.12 | 0.07 | 0.02 | 0.02 | 0.00 | 0.00 | 0.00 | 0.00 | 0.00 | 0.00 | 0.00 | 0.00 | 0.00 | 0.00 | 2.60 |
|  |  | E | D | A | R | T | S | G | Q | P | V | K | N | H | L | F | I | Y | M | C | W | Total |
| E | 4946 | 4202 | 241 | 131 | 58 | 54 | 53 | 47 | 39 | 26 | 23 | 23 | 18 | 9 | 8 | 5 | 4 | 2 | 2 | 1 | 0 | 744 |
|  |  | 5.95 | 4.35 | 2.36 | 1.05 | 0.97 | 0.96 | 0.85 | 0.70 | 0.47 | 0.42 | 0.42 | 0.32 | 0.16 | 0.14 | 0.09 | 0.07 | 0.04 | 0.04 | 0.02 | 0.00 | 13.43 |
|  |  | 84.96 | 4.87 | 2.65 | 1.17 | 1.09 | 1.07 | 0.95 | 0.79 | 0.53 | 0.47 | 0.47 | 0.36 | 0.18 | 0.16 | 0.10 | 0.08 | 0.04 | 0.04 | 0.02 | 0.00 | 15.04 |
|  |  | K | R | Q | T | N | E | G | S | M | A | D | V | H | Y | L | P | W | F | I | C | Total |
| K | 1763 | 1674 | 27 | 12 | 8 | 8 | 6 | 5 | 5 | 4 | 4 | 4 | 3 | 1 | 1 | 1 | 0 | 0 | 0 | 0 | 0 | 89 |
|  |  | 2.37 | 0.49 | 0.22 | 0.14 | 0.14 | 0.11 | 0.09 | 0.09 | 0.07 | 0.07 | 0.07 | 0.05 | 0.02 | 0.02 | 0.02 | 0.00 | 0.00 | 0.00 | 0.00 | 0.00 | 1.61 |
|  |  | 94.95 | 1.53 | 0.68 | 0.45 | 0.45 | 0.34 | 0.28 | 0.28 | 0.23 | 0.23 | 0.23 | 0.17 | 0.06 | 0.06 | 0.06 | 0.00 | 0.00 | 0.00 | 0.00 | 0.00 | 5.05 |
|  |  | R | E | A | K | T | Q | D | L | G | V | H | S | N | Y | I | P | F | M | W | C | Total |
| R | 5463 | 5085 | 46 | 45 | 45 | 34 | 30 | 29 | 28 | 24 | 23 | 18 | 18 | 10 | 8 | 7 | 6 | 5 | 2 | 0 | 0 | 378 |
|  |  | 7.20 | 0.83 | 0.81 | 0.81 | 0.61 | 0.54 | 0.52 | 0.51 | 0.43 | 0.42 | 0.32 | 0.32 | 0.18 | 0.14 | 0.13 | 0.11 | 0.09 | 0.04 | 0.00 | 0.00 | 6.82 |
|  |  | 93.08 | 0.84 | 0.82 | 0.82 | 0.62 | 0.55 | 0.53 | 0.51 | 0.44 | 0.42 | 0.33 | 0.33 | 0.18 | 0.15 | 0.13 | 0.11 | 0.09 | 0.04 | 0.00 | 0.00 | 6.92 |
|  |  | H | R | Y | A | E | S | G | F | Q | N | T | L | D | V | P | M | K | W | I | C | Total |
| H | 1942 | 1807 | 23 | 15 | 15 | 12 | 11 | 10 | 9 | 9 | 8 | 6 | 5 | 4 | 3 | 2 | 2 | 1 | 0 | 0 | 0 | 135 |
|  |  | 2.56 | 0.42 | 0.27 | 0.27 | 0.22 | 0.20 | 0.18 | 0.16 | 0.16 | 0.14 | 0.11 | 0.09 | 0.07 | 0.05 | 0.04 | 0.04 | 0.02 | 0.00 | 0.00 | 0.00 | 2.44 |
|  |  | 93.05 | 1.18 | 0.77 | 0.77 | 0.62 | 0.57 | 0.51 | 0.46 | 0.46 | 0.41 | 0.31 | 0.26 | 0.21 | 0.15 | 0.10 | 0.10 | 0.05 | 0.00 | 0.00 | 0.00 | 6.95 |
|  |  | Q | R | E | A | G | D | H | K | T | S | L | N | M | V | Y | P | W | F | I | C | Total |
| Q | 1671 | 1491 | 37 | 33 | 19 | 16 | 14 | 10 | 10 | 8 | 8 | 7 | 7 | 4 | 4 | 2 | 1 | 0 | 0 | 0 | 0 | 180 |
|  |  | 2.11 | 0.67 | 0.60 | 0.34 | 0.29 | 0.25 | 0.18 | 0.18 | 0.14 | 0.14 | 0.13 | 0.13 | 0.07 | 0.07 | 0.04 | 0.02 | 0.00 | 0.00 | 0.00 | 0.00 | 3.25 |
|  |  | 89.23 | 2.21 | 1.97 | 1.14 | 0.96 | 0.84 | 0.60 | 0.60 | 0.48 | 0.48 | 0.42 | 0.42 | 0.24 | 0.24 | 0.12 | 0.06 | 0.00 | 0.00 | 0.00 | 0.00 | 10.77 |
|  |  | N | D | S | T | A | E | G | R | K | L | Q | P | V | H | Y | F | M | W | I | C | Total |
| N | 1758 | 1642 | 28 | 18 | 12 | 10 | 10 | 7 | 6 | 6 | 4 | 4 | 3 | 3 | 2 | 1 | 1 | 1 | 0 | 0 | 0 | 116 |
|  |  | 2.33 | 0.51 | 0.32 | 0.22 | 0.18 | 0.18 | 0.13 | 0.11 | 0.11 | 0.07 | 0.07 | 0.05 | 0.05 | 0.04 | 0.02 | 0.02 | 0.02 | 0.00 | 0.00 | 0.00 | 2.09 |
|  |  | 93.40 | 1.59 | 1.02 | 0.68 | 0.57 | 0.57 | 0.40 | 0.34 | 0.34 | 0.23 | 0.23 | 0.17 | 0.17 | 0.11 | 0.06 | 0.06 | 0.06 | 0.00 | 0.00 | 0.00 | 6.60 |
|  |  | C | A | S | V | I | L | P | G | Q | E | D | H | Y | W | F | M | T | N | R | K | Total |
| C | 682 | 656 | 7 | 5 | 4 | 3 | 2 | 1 | 1 | 1 | 1 | 1 | 0 | 0 | 0 | 0 | 0 | 0 | 0 | 0 | 0 | 26 |
|  |  | 0.93 | 0.13 | 0.09 | 0.07 | 0.05 | 0.04 | 0.02 | 0.02 | 0.02 | 0.02 | 0.02 | 0.00 | 0.00 | 0.00 | 0.00 | 0.00 | 0.00 | 0.00 | 0.00 | 0.00 | 0.47 |
|  |  | 96.19 | 1.03 | 0.73 | 0.59 | 0.44 | 0.29 | 0.15 | 0.15 | 0.15 | 0.15 | 0.15 | 0.00 | 0.00 | 0.00 | 0.00 | 0.00 | 0.00 | 0.00 | 0.00 | 0.00 | 3.81 |
|  |  | S | A | T | D | G | E | P | V | R | N | F | L | Q | H | Y | M | I | K | W | C | Total |
| S | 2728 | 2384 | 82 | 54 | 48 | 44 | 40 | 21 | 13 | 13 | 12 | 3 | 3 | 3 | 2 | 2 | 2 | 1 | 1 | 0 | 0 | 344 |
|  |  | 3.38 | 1.48 | 0.97 | 0.87 | 0.79 | 0.72 | 0.38 | 0.23 | 0.23 | 0.22 | 0.05 | 0.05 | 0.05 | 0.04 | 0.04 | 0.04 | 0.02 | 0.02 | 0.00 | 0.00 | 6.21 |
|  |  | 87.39 | 3.01 | 1.98 | 1.76 | 1.61 | 1.47 | 0.77 | 0.48 | 0.48 | 0.44 | 0.11 | 0.11 | 0.11 | 0.07 | 0.07 | 0.07 | 0.04 | 0.04 | 0.00 | 0.00 | 12.61 |
|  |  | T | S | A | V | R | D | E | G | N | I | P | L | Q | M | H | C | K | Y | F | W | Total |
| T | 3847 | 3384 | 97 | 82 | 65 | 37 | 34 | 29 | 28 | 18 | 15 | 14 | 13 | 10 | 7 | 6 | 4 | 2 | 1 | 1 | 0 | 463 |
|  |  | 4.79 | 1.75 | 1.48 | 1.17 | 0.67 | 0.61 | 0.52 | 0.51 | 0.32 | 0.27 | 0.25 | 0.23 | 0.18 | 0.13 | 0.11 | 0.07 | 0.04 | 0.02 | 0.02 | 0.00 | 8.36 |
|  |  | 87.96 | 2.52 | 2.13 | 1.69 | 0.96 | 0.88 | 0.75 | 0.73 | 0.47 | 0.39 | 0.36 | 0.34 | 0.26 | 0.18 | 0.16 | 0.10 | 0.05 | 0.03 | 0.03 | 0.00 | 12.04 |
|  |  | G | A | D | S | E | N | T | P | R | V | Q | K | H | F | M | L | I | W | C | Y | Total |
| G | 8282 | 7921 | 104 | 74 | 42 | 34 | 21 | 18 | 17 | 16 | 9 | 5 | 5 | 4 | 3 | 3 | 2 | 2 | 1 | 1 | 0 | 361 |
|  |  | 11.22 | 1.88 | 1.34 | 0.76 | 0.61 | 0.38 | 0.32 | 0.31 | 0.29 | 0.16 | 0.09 | 0.09 | 0.07 | 0.05 | 0.05 | 0.04 | 0.04 | 0.02 | 0.02 | 0.00 | 6.52 |
|  |  | 95.64 | 1.26 | 0.89 | 0.51 | 0.41 | 0.25 | 0.22 | 0.21 | 0.19 | 0.11 | 0.06 | 0.06 | 0.05 | 0.04 | 0.04 | 0.02 | 0.02 | 0.01 | 0.01 | 0.00 | 4.36 |
|  |  | A | V | S | G | T | E | D | R | P | L | I | C | Q | N | K | H | Y | F | M | W | Total |
| A | 6914 | 6338 | 114 | 84 | 81 | 73 | 53 | 46 | 33 | 28 | 15 | 10 | 8 | 8 | 5 | 5 | 4 | 4 | 4 | 1 | 0 | 576 |
|  |  | 8.98 | 2.06 | 1.52 | 1.46 | 1.32 | 0.96 | 0.83 | 0.60 | 0.51 | 0.27 | 0.18 | 0.14 | 0.14 | 0.09 | 0.09 | 0.07 | 0.07 | 0.07 | 0.02 | 0.00 | 10.40 |
|  |  | 91.67 | 1.65 | 1.21 | 1.17 | 1.06 | 0.77 | 0.67 | 0.48 | 0.40 | 0.22 | 0.14 | 0.12 | 0.12 | 0.07 | 0.07 | 0.06 | 0.06 | 0.06 | 0.01 | 0.00 | 8.33 |
|  |  | V | I | A | L | T | E | P | F | M | R | G | D | S | Y | H | N | W | C | Q | K | Total |
| V | 5405 | 4886 | 171 | 118 | 88 | 63 | 16 | 11 | 11 | 11 | 8 | 7 | 5 | 4 | 3 | 2 | 1 | 0 | 0 | 0 | 0 | 519 |
|  |  | 6.92 | 3.09 | 2.13 | 1.59 | 1.14 | 0.29 | 0.20 | 0.20 | 0.20 | 0.14 | 0.13 | 0.09 | 0.07 | 0.05 | 0.04 | 0.02 | 0.00 | 0.00 | 0.00 | 0.00 | 9.37 |
|  |  | 90.40 | 3.16 | 2.18 | 1.63 | 1.17 | 0.30 | 0.20 | 0.20 | 0.20 | 0.15 | 0.13 | 0.09 | 0.07 | 0.06 | 0.04 | 0.02 | 0.00 | 0.00 | 0.00 | 0.00 | 9.60 |
|  |  | I | V | L | M | A | T | F | Y | N | G | S | E | P | R | D | H | W | C | Q | K | Total |
| I | 2128 | 1864 | 144 | 71 | 12 | 8 | 8 | 6 | 3 | 3 | 2 | 2 | 2 | 1 | 1 | 1 | 0 | 0 | 0 | 0 | 0 | 264 |
|  |  | 2.64 | 2.60 | 1.28 | 0.22 | 0.14 | 0.14 | 0.11 | 0.05 | 0.05 | 0.04 | 0.04 | 0.04 | 0.02 | 0.02 | 0.02 | 0.00 | 0.00 | 0.00 | 0.00 | 0.00 | 4.76 |
|  |  | 87.59 | 6.77 | 3.34 | 0.56 | 0.38 | 0.38 | 0.28 | 0.14 | 0.14 | 0.09 | 0.09 | 0.09 | 0.05 | 0.05 | 0.05 | 0.00 | 0.00 | 0.00 | 0.00 | 0.00 | 12.41 |
|  |  | L | V | I | M | F | A | T | Y | R | C | S | P | G | D | Q | E | H | W | N | K | Total |
| L | 6830 | 6361 | 119 | 110 | 59 | 40 | 34 | 21 | 15 | 14 | 12 | 9 | 8 | 7 | 7 | 5 | 4 | 3 | 1 | 1 | 0 | 469 |
|  |  | 9.01 | 2.15 | 1.99 | 1.06 | 0.72 | 0.61 | 0.38 | 0.27 | 0.25 | 0.22 | 0.16 | 0.14 | 0.13 | 0.13 | 0.09 | 0.07 | 0.05 | 0.02 | 0.02 | 0.00 | 8.46 |
|  |  | 93.13 | 1.74 | 1.61 | 0.86 | 0.59 | 0.50 | 0.31 | 0.22 | 0.20 | 0.18 | 0.13 | 0.12 | 0.10 | 0.10 | 0.07 | 0.06 | 0.04 | 0.01 | 0.01 | 0.00 | 6.87 |
|  |  | P | A | D | S | E | V | T | G | L | R | H | I | N | K | F | Q | Y | M | C | W | Total |
| P | 4555 | 4303 | 60 | 36 | 29 | 25 | 23 | 22 | 16 | 9 | 8 | 6 | 5 | 3 | 3 | 2 | 2 | 1 | 1 | 1 | 0 | 252 |
|  |  | 6.10 | 1.08 | 0.65 | 0.52 | 0.45 | 0.42 | 0.40 | 0.29 | 0.16 | 0.14 | 0.11 | 0.09 | 0.05 | 0.05 | 0.04 | 0.04 | 0.02 | 0.02 | 0.02 | 0.00 | 4.55 |
|  |  | 94.47 | 1.32 | 0.79 | 0.64 | 0.55 | 0.50 | 0.48 | 0.35 | 0.20 | 0.18 | 0.13 | 0.11 | 0.07 | 0.07 | 0.04 | 0.04 | 0.02 | 0.02 | 0.02 | 0.00 | 5.53 |
|  |  | M | L | V | T | S | I | D | A | F | R | Q | K | H | P | Y | G | W | C | N | E | Total |
| M | 1458 | 1365 | 31 | 9 | 8 | 8 | 7 | 6 | 5 | 4 | 4 | 3 | 3 | 2 | 1 | 1 | 1 | 0 | 0 | 0 | 0 | 93 |
|  |  | 1.93 | 0.56 | 0.16 | 0.14 | 0.14 | 0.13 | 0.11 | 0.09 | 0.07 | 0.07 | 0.05 | 0.05 | 0.04 | 0.02 | 0.02 | 0.02 | 0.00 | 0.00 | 0.00 | 0.00 | 1.68 |
|  |  | 93.62 | 2.13 | 0.62 | 0.55 | 0.55 | 0.48 | 0.41 | 0.34 | 0.27 | 0.27 | 0.21 | 0.21 | 0.14 | 0.07 | 0.07 | 0.07 | 0.00 | 0.00 | 0.00 | 0.00 | 6.38 |
|  |  | F | Y | L | V | I | M | A | R | W | T | S | E | P | H | G | C | Q | N | K | D | Total |
| F | 2777 | 2638 | 38 | 36 | 15 | 8 | 7 | 7 | 6 | 4 | 3 | 3 | 3 | 2 | 2 | 2 | 2 | 1 | 0 | 0 | 0 | 139 |
|  |  | 3.74 | 0.69 | 0.65 | 0.27 | 0.14 | 0.13 | 0.13 | 0.11 | 0.07 | 0.05 | 0.05 | 0.05 | 0.04 | 0.04 | 0.04 | 0.04 | 0.02 | 0.00 | 0.00 | 0.00 | 2.51 |
|  |  | 94.99 | 1.37 | 1.30 | 0.54 | 0.29 | 0.25 | 0.25 | 0.22 | 0.14 | 0.11 | 0.11 | 0.11 | 0.07 | 0.07 | 0.07 | 0.07 | 0.04 | 0.00 | 0.00 | 0.00 | 5.01 |
|  |  | W | F | Y | A | H | E | P | L | R | I | G | T | C | D | M | V | S | N | Q | K | Total |
| W | 994 | 855 | 110 | 8 | 4 | 3 | 3 | 2 | 2 | 2 | 1 | 1 | 1 | 1 | 1 | 0 | 0 | 0 | 0 | 0 | 0 | 139 |
|  |  | 1.21 | 1.99 | 0.14 | 0.07 | 0.05 | 0.05 | 0.04 | 0.04 | 0.04 | 0.02 | 0.02 | 0.02 | 0.02 | 0.02 | 0.00 | 0.00 | 0.00 | 0.00 | 0.00 | 0.00 | 2.51 |
|  |  | 86.02 | 11.07 | 0.80 | 0.40 | 0.30 | 0.30 | 0.20 | 0.20 | 0.20 | 0.10 | 0.10 | 0.10 | 0.10 | 0.10 | 0.00 | 0.00 | 0.00 | 0.00 | 0.00 | 0.00 | 13.98 |
|  |  | Y | F | H | L | V | T | A | R | G | S | N | E | D | W | M | Q | P | I | C | K | Total |
| Y | 2401 | 2252 | 57 | 25 | 12 | 12 | 8 | 6 | 6 | 3 | 3 | 3 | 3 | 3 | 2 | 2 | 2 | 1 | 1 | 0 | 0 | 149 |
|  |  | 3.19 | 1.03 | 0.45 | 0.22 | 0.22 | 0.14 | 0.11 | 0.11 | 0.05 | 0.05 | 0.05 | 0.05 | 0.05 | 0.04 | 0.04 | 0.04 | 0.02 | 0.02 | 0.00 | 0.00 | 2.69 |
|  |  | 93.79 | 2.37 | 1.04 | 0.50 | 0.50 | 0.33 | 0.25 | 0.25 | 0.12 | 0.12 | 0.12 | 0.12 | 0.12 | 0.08 | 0.08 | 0.08 | 0.04 | 0.04 | 0.00 | 0.00 | 6.21 |

NOTE: The total number of aligned residues was 70,589, of which 5,541 residues were substituted and 65,048 residues were conserved in *H. lacusprofundi*.
